# *Fusobacterium nucleatum* promotes tumor progression in *KRAS* p.G12D-mutant colorectal cancer by binding to DHX15

**DOI:** 10.1038/s41467-024-45572-w

**Published:** 2024-02-24

**Authors:** Huiyuan Zhu, Man Li, Dexi Bi, Huiqiong Yang, Yaohui Gao, Feifei Song, Jiayi Zheng, Ruting Xie, Youhua Zhang, Hu Liu, Xuebing Yan, Cheng Kong, Yefei Zhu, Qian Xu, Qing Wei, Huanlong Qin

**Affiliations:** 1 Department of Pathology, Shanghai Tenth People’s Hospital, Tongji University School of Medicine, Shanghai, 200072 China; 2 Department of Oncology, Yangzhou University Medical College Affiliated Hospital, Yangzhou, 225000 China; 3 Department of Colorectal Surgery, Fudan University Shanghai Cancer Center, Shanghai, 200032 China; 4 Research Institute of Intestinal Diseases, Tongji University School of Medicine, Shanghai, 200072 China; 5 Department of Gastrointestinal Surgery, Shanghai Tenth People’s Hospital, Tongji University School of Medicine, Shanghai, 200072 China

**Keywords:** Cellular microbiology, Colorectal cancer, Microbiome

## Abstract

*Fusobacterium nucleatum* (*F. nucleatum*) promotes intestinal tumor growth and its relative abundance varies greatly among patients with CRC, suggesting the presence of unknown, individual-specific effectors in *F. nucleatum*-dependent carcinogenesis. Here, we identify that *F. nucleatum* is enriched preferentially in *KRAS* p.G12D mutant CRC tumor tissues and contributes to colorectal tumorigenesis in *Villin-Cre/Kras*^*G12D+/-*^ mice. Additionally, *Parabacteroides distasonis* (*P. distasonis*) competes with *F. nucleatum* in the G12D mouse model and human CRC tissues with the *KRAS* mutation. Orally gavaged *P. distasonis* in mice alleviates the *F. nucleatum*-dependent CRC progression. *F. nucleatum* invades intestinal epithelial cells and binds to DHX15, a protein of RNA helicase family expressed on CRC tumor cells, mechanistically involving ERK/STAT3 signaling. Knock out of *Dhx15* in *Villin-Cre/Kras*^*G12D+/-*^ mice attenuates the CRC phenotype. These findings reveal that the oncogenic effect of *F. nucleatum* depends on somatic genetics and gut microbial ecology and indicate that personalized modulation of the gut microbiota may provide a more targeted strategy for CRC treatment.

## Abstract (web summary)

Several studies have shown that *Fusobacterium nucleatum* aggravates colorectal cancer (CRC) development and chemoresistance. Here the authors show that *F. nucleatum* is enriched preferentially in patients with *KRAS* p.G12D mutant CRC and that it promotes colorectal tumorigenesis in preclinical models by binding DHX15 on tumor cells.

## Introduction

Colorectal cancer is the second most common malignancy worldwide and is characterized by specific somatic mutations including lesions in oncogenes, tumor suppressor genes, and DNA repair-related genes^1,2^. According to The Cancer Genome Atlas (TCGA) database, *APC*, *TP53*, *KRAS*, and *SMAD4* are the four most frequently mutated genes in patients with CRC, and their alterations often exhibit prognostic relevance^3^. For example, *KRAS* mutations are found in about half of the human CRC cases and recognized as critical determinants of therapeutic response^4^. In *KRAS*, the codon 12 of exon 2 is the most prevalent site of mutation in CRC (mainly p.G12D). As a clinical interest, there are currently no drugs that can effectively treat the *KRAS* p.G12D-expressing tissues^5^.

In addition to genetic mutations, other local factors, most notably gut microbiota, also influence CRC occurrence and progression^6^. Recent studies have shown that the genotoxic pathogenicity island in *Escherichia coli* causes a distinct mutational signature in clonal organoids^7^ and that a gut microbiota-generated metabolite, gallic acid, switches mutant p53 from tumor-suppressive to oncogenic^8^. These observations suggest that in the pathogenesis of CRC, there are cross-talks between gut microbiota and local somatic genotypes.

Cross-cohort meta-analyses have captured CRC-associated gut bacteria that were consistently identified in the cancer population spanning different continents^9,10^, and they include *Fusobacterium nucleatum*, *Parvimonas micra*, *Gemella morbillorum*, and *Peptostreptococcus stomatis*. Although the etiologic connection remains elusive for many of the convergent microbial signatures, considerable knowledge has been gained on the versatile roles of *F. nucleatum*. Studies have shown that *F. nucleatum* aggravates CRC development and chemoresistance via multiple routes including attachment to epithelial cells, modulation of immune microenvironment, promotion of cell cycle, activation of TLR4 signaling, and regulation of autophagy^11–14^. Whereas some gut microbes potentiate CRC tumor growth, others play a beneficial role. A recent mouse study involving a collection of 11 bacterial strains isolated from healthy human donors showed that the microbial intervention significantly improved the efficacy of anti-PD-1 treatment^15^. In addition, anti-CRC effect was also observed after introduction of single gut microbes, such as *Lactobacillus acidophilus*^16^ and *Parabacteroides distasonis* (*P. distasonis*)^17^. *P. distasonis* is defined as one of the 18 core members in the gut microbiota of human^18^ and thought to have important physiological functions in host^19^. Although these results provide the lead of substantial clinical interest, greater mechanistic insights are needed to facilitate the translation of gut microbiota-based findings.

Here, we attempt to elucidate the host and gut microbial factors crucial for *F. nucleatum*-dependent CRC progression. Our findings indicate that the oncogenic role of *F. nucleatum* is influenced by the genotype of somatic tissue, a putative host RNA helicase, and a *F. nucleatum*-competing gut bacterium. The results illustrate the intricacy of local effectors in modulating *F. nucleatum*-dependent CRC development, which may inform a more effective, personalized intervention for the disease.

## Results

### F. nucleatum is enriched preferentially in KRAS p.G12D-mutant CRC tissues

**Fig. 1:**
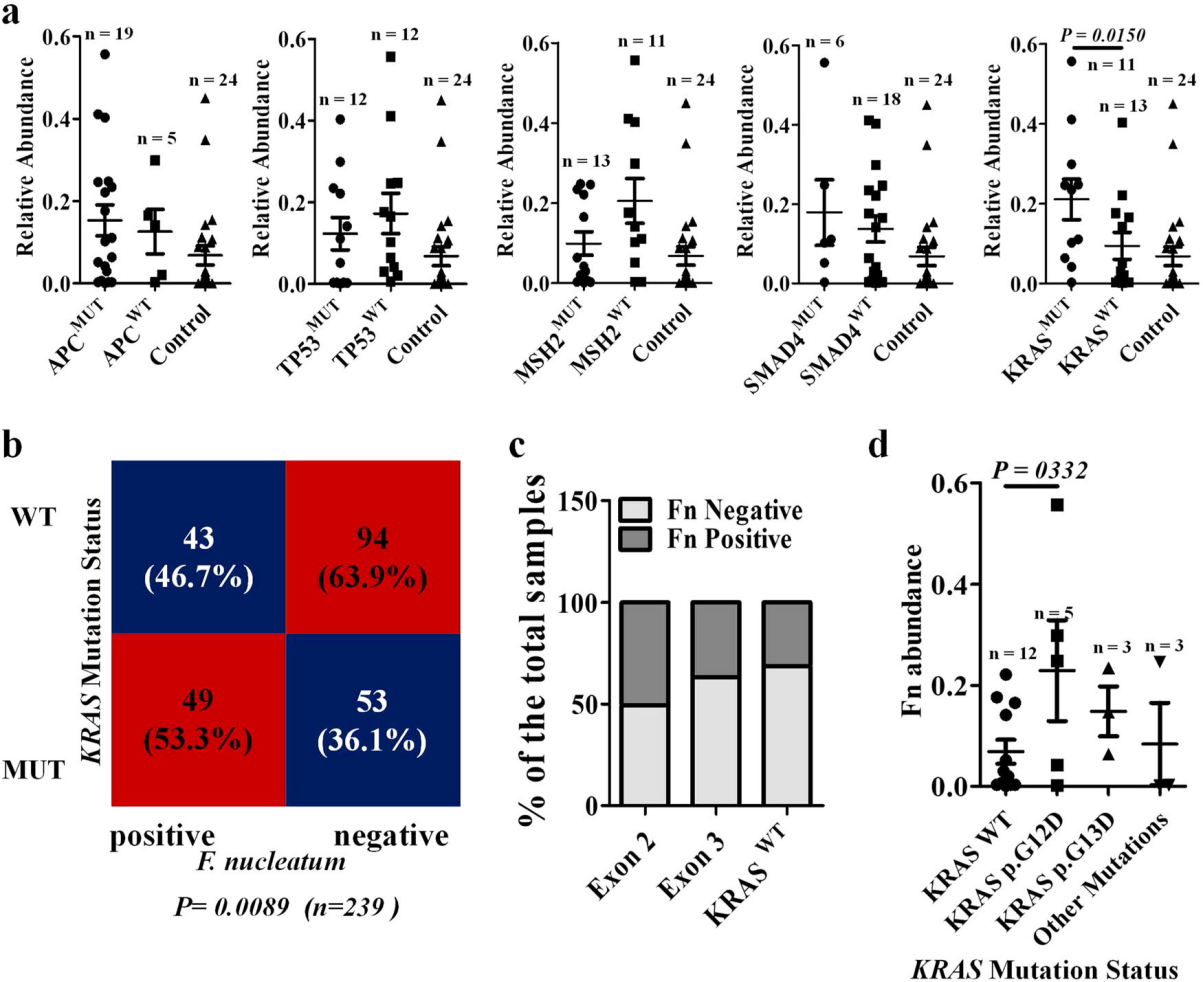
*F. nucleatum* is enriched preferentially in *KRAS* p.G12D-mutant CRC tumor tissues. **a** Exon sequencing and qPCR detection of the mutation status of CRC tissues and *F. nucleatum* abundance in the indicated group. The relative abundance of *F. nucleatum* was confirmed using abundance of *F. nucleatum* /quantity of pgt gene. Significant differences are indicated: one-way ANOVA with Sidak’s multiple comparison test, data are presented as the mean ± SEM. **b** Correlation of *KRAS* mutation status and *F. nucleatum* abundance in CRC tumor tissues. Significant differences are indicated: Chi-square test, two-sided, *n* = 239. **c** *F. nucleatum* (Fn) positive rates under different *KRAS* mutation types in tumor tissues of CRC (*n* = 239). **d** Relative abundance of *F. nucleatum* in the CRC patients of the indicated groups. Significant differences are indicated: one-way ANOVA with Bonferroni’s multiple comparison test, data are presented as the mean ± SEM. Source data are provided as a Source Data file.

To investigate the correlation between *F. nucleatum* abundance and CRC somatic mutations, we performed quantitative polymerase chain reaction (qPCR) and exon sequencing on 24 pairs of tumor and matched paratumor tissues. The tumor samples were divided into wild-type (WT) tissues and mutation-bearing tissues based on exon sequencing results. Subsequent analysis revealed that *F. nucleatum* abundance in the *KRAS* mutant tissues was higher than that in the *KRAS* WT tissues and paratumor tissues (Fig. 1a), which was validated in an independent cohort of 239 CRC patients (CRC patients information are shown in Supplementary Table 1) (Fig. 1b). In comparison, *F. nucleatum* abundance exhibited no association with mutation in four tested genes namely *APC*, *TP53*, *MSH2*, and *SMAD4*.

Among patients with CRC, mutations in *KRAS* concentrate at codons 12, 13, and 61 of exon 2 and vary greatly in prevalence^20^. To examine whether *F. nucleatum* level correlated with particular genetic variants, we analyzed the *KRAS* mutation profile and *F. nucleatum* abundance in cohort 2 by qPCR. We found that *F. nucleatum* was detected in 42/83 (50.6%) tumors with a *KRAS* mutation in exon 2, 7/19 (36.8%) tumors with a *KRAS* mutation in exon 3 and 43/137 (31.4%) in *KRAS* WT tumors (Fig. 1c). Interestingly, the tumors with the p.G12D mutation, but not those with the adjacent p.G13D mutation, accommodated significantly higher *F. nucleatum* abundance than WT tumors (Fig. 1d). Collectively, our data bridge a local connection between *F. nucleatum* abundance and *KRAS* genotype.

### F. nucleatum exacerbates colorectal tumorigenesis in Villin-Cre/Kras^G12D+/−^ mice

**Fig. 2:**
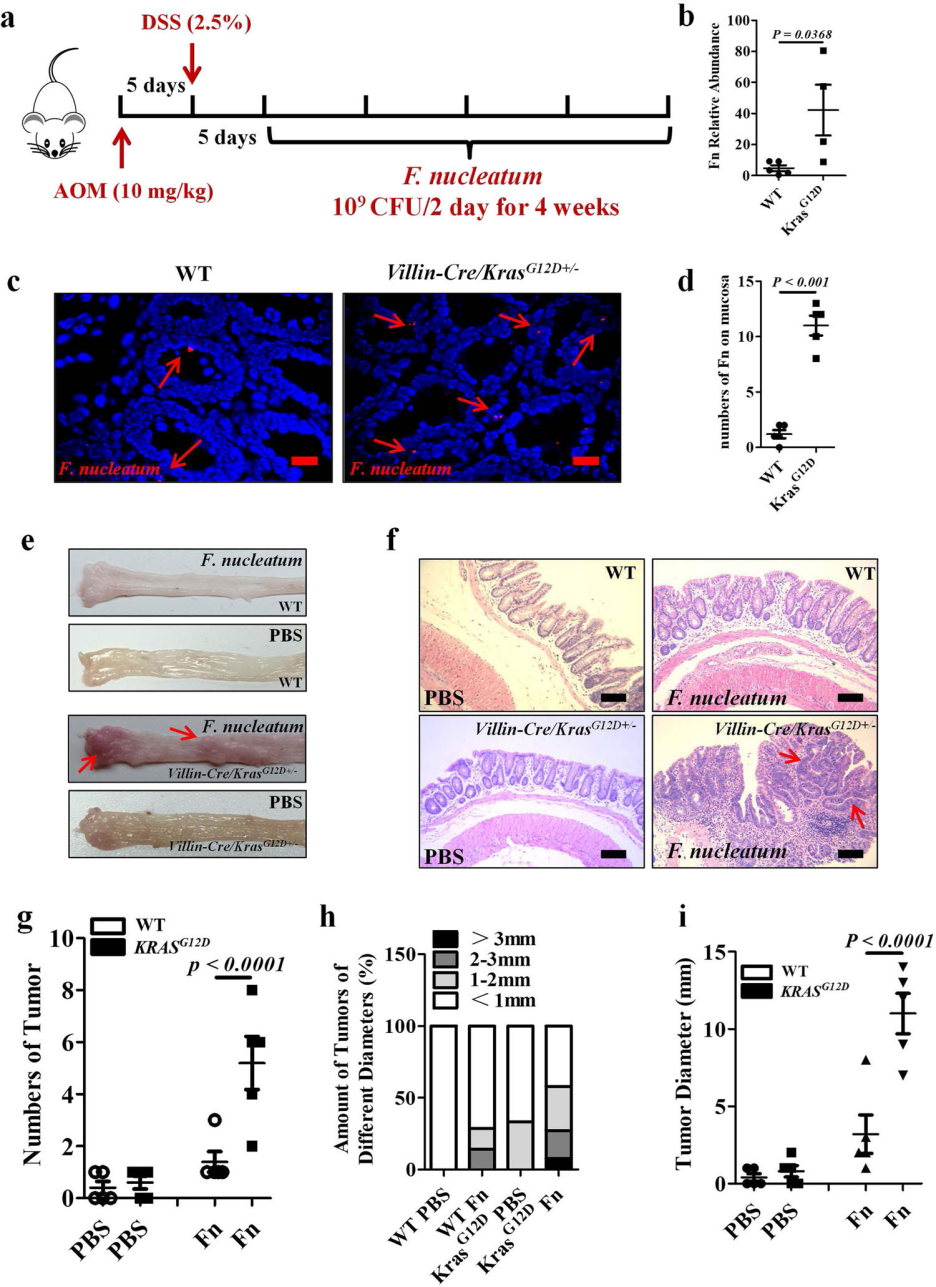
*F. nucleatum* exacerbates colorectal tumorigenesis in *Villin-Cre/Kras*^*G12D+/*^^−^ mice. **a** Schematic diagram of the experimental design and timeline of mouse models. **b** qPCR analysis of *F. nucleatum* abundance in colonic tissues derived from *Villin-Cre/Kras*^*G12D+/*^^−^ (*Kras*^G12D^) mice and WT littermates (WT) treated with AOM/DSS and *F. nucleatum*. Significant differences are indicated: two-tailed Student’s *t*-test, *n* = 5 (WT) and *n* = 4 (*Kras*^G12D^) respectively, data are presented as the mean ± SEM. **c** FISH detection of *F. nucleatum* in colonic tissues derived from *Villin-Cre/Kras*^*G12D+/*^^−^ mice and WT littermates using a Cy3-conjugated *F. nucleatum* specific probe, the red arrows indicate *F. nucleatum*, *n* = 5, scale bar: 50 μm. **d** Statistical analysis of the results in (**c**). Significant differences are indicated: two-tailed Student’s *t*-test, *n* = 5 per group, data are presented as the mean ± SEM. **e**, **f** Representative images and H&E stainings of the colons of WT littermates and *Villin-Cre/Kras*^*G12D+/*^^−^ mice treated with AOM/DSS and PBS or *F. nucleatum*, the red arrows indicate tumors, *n* = 5 per group, scale bar: 50 μm. **g**–**i** Tumor numbers, tumor loads, and size of *Villin-Cre/Kras*^*G12D+/*^^−^ mice and WT littermates treated with AOM/DSS and PBS or *F. nucleatum*. Significant differences are indicated: one-way ANOVA with Bonferroni’s multiple comparison test, *n* = 5 per group, data are presented as the mean ± SEM. Data are representative of three independent experiments. Source data are provided as a Source Data file.

To investigate the mechanistic underpinning of the observed *F. nucleatum*-*KRAS* (p.G12D) link, we successfully generated *Villin-Cre/Kras*^*G12D+/*^^−^ mice (Supplementary Fig. 1a, b). We hypothesized that *F. nucleatum* colonization would be increased in the colon of *Villin-Cre/Kras*^*G12D+/*^^−^ mice, thus aggravating tumorigenesis. To test this, we developed an azoxymethane/dextran sulfate sodium (AOM/DSS)-induced CRC mouse model (the mice were intraperitoneally injected with 10 mg/kg AOM, followed by 5-day oral administration of 2.5% DSS starting 5 days later) in both *Villin-Cre/Kras*^*G12D+/*^^−^ and *Villin-Cre/Kras*^*G12D*^^−^^*/*^^−^ (WT) backgrounds and gavaged the mice with *F. nucleatum* (1 × 10^9^ CFU) or phosphate-buffered saline (PBS) as shown in Fig. 2a. qPCR and fluorescence in situ hybridization (FISH) assays revealed that *F. nucleatum* was enriched in *Villin-Cre/Kras*^*G12D+/*^^−^ colonic tissues compared with that in the WT tissues (Fig. 2b–d) but undetectable in the tissues of PBS-treated rodents, regardless of genetic backgrounds (Supplementary Fig. 1c). Moreover, we showed that *F. nucleatum*-treated *Villin-Cre/Kras*^*G12D+/*^^−^ mice exhibited a pronounced augmentation in tumor formation compared with *F. nucleatum*-treated WT littermates and PBS-treated animals (Fig. 2e). Hematoxylin and eosin (H&E) staining revealed that *F. nucleatum*-treated *Villin-Cre/Kras*^*G12D+/*^^−^ mice exhibited higher grades of dysplasia (Fig. 2f), tumor multiplicities and tumor loads than *F. nucleatum*-treated WT littermates (Fig. 2g–i). Additionally, to evaluate the interaction of *F. nucleatum* with the immune system in *KRAS* mutant mice and patients, immunofluorescence (IF) and qPCR were performed to evaluate the infiltrated immune cells and inflammatory cytokine production in colonic tissues of PBS and *F. nucleatum*-treated *Villin-Cre/Kras*^*G12D+/*^^−^ mice. We found that *F. nucleatum* led to less CD3^+^ T cells infiltrating which is consisting with the study that *F. nucleatum* was inversely associated with tumor stromal CD3^+^ lymphocytes (Supplementary Fig. 1d, e)^21^. However, there were no significant differences in the number of infiltrated CD11c^+^ dendritic cells in colonic tissues between PBS and *F. nucleatum-treated* *Villin-Cre/Kras*^*G12D+/*^^−^ mice (Supplementary Fig. 1d, e). The expression of *Il-17a*, *Il-6* were also elevated after *F. nucleatum* treatment in *Villin-Cre/Kras*^*G12D+/*^^−^ mice (Supplementary Fig. 1f). And we confirmed the results of infiltrated immune cells in tumor tissues from *KRAS* G12D-mutant patients by IF (Supplementary Fig. 1g, h). Together, our results suggest that *F. nucleatum*-mediated-colorectal tumorigenesis is aggravated in mice harboring *KRAS* p.G12D mutant via its increased colonization.

### P. distasonis competes with F. nucleatum in a Villin-Cre/Kras^G12D+/−^ mouse model and human KRAS mutant CRC tissues

**Fig. 3:**
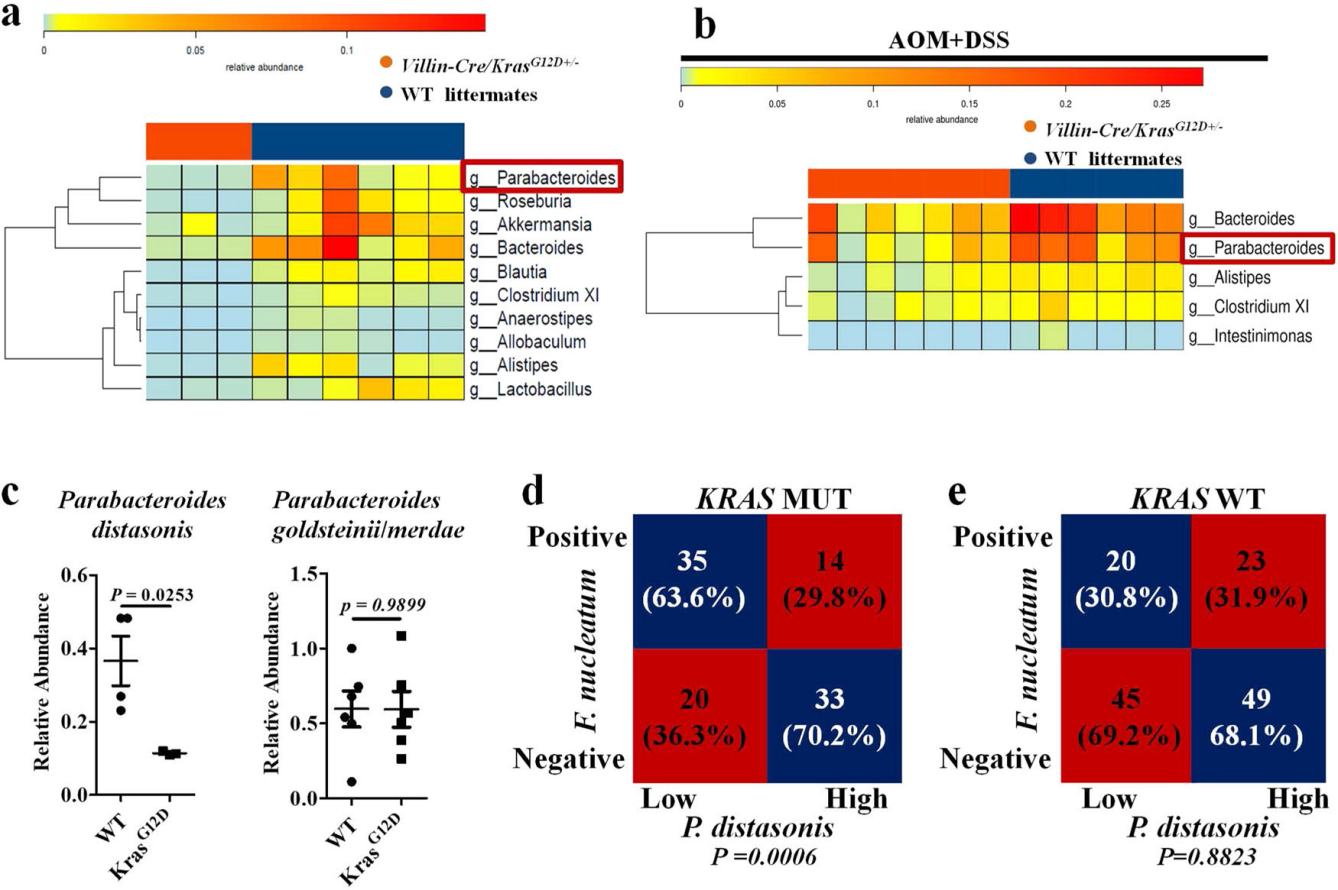
*P. distasonis* competes with *F. nucleatum* in *a Villin-Cre/Kras*^*G12D+/*^^−^ mouse model and *KRAS* mutant CRC tissues. **a** Heat map of differentially abundant genera between *Villin-Cre/Kras*^*G12D+/*^^−^ mice (*n* = 3) and WT littermates (*n* = 6) treated with *F. nucleatum* (1 × 10^9^ CFU) every two days for four weeks. **b** Heat map of top differentially abundant genera between *Villin-Cre/Kras*^*G12D+/*^^−^ (*n* = 7) mice and WT littermates (*n* = 6) which treated by AOM/DSS and *F. nucleatum*. **c** qPCR analysis of *P. distasonis* and *P. goldsteinii*/*merdae* abundance in colonic tissues derived from *Villin-Cre/Kras*^*G12D+/*^^−^ mice and WT littermates treated by AOM/DSS and *F. nucleatum*. Significant differences are indicated: two-tailed Student’s *t*-test, *n* = 4 (WT *P. distasonis*), *n* = 3 (*Kras*^G12D^ *P. distasonis*), and *n* = 6 (*P. goldsteinii*/*merdae*) respectively, data are presented as the mean ± SEM. **d**, **e** Correlation analysis of *F. nucleatum* and *P. distasonis* abundance in *KRAS* mutant (*n* = 102) and *KRAS* WT (*n* = 137) CRC patients. Significant differences are indicated: Chi-square test, two-sided. Source data are provided as a Source Data file.

Given that gut microbiota is a complex ecosystem, we were curious about whether the connection between *F. nucleatum* and *KRAS* (p.G12D) involved other bacteria. We first surveyed the microbiota compositions of the colon tissues from WT and *Villin-Cre/Kras*^*G12D+/*^^−^ mice and discovered that *Clostridium XIVa* and *Ralstonia* were depleted in the G12D animals (Supplementary Fig. 2a), although, no differences in the appearance, colon length and histological presentation of the colon were observed between the two groups (Supplementary Fig. 2b–d). These findings suggested that the p.G12D mutation affected the gut microbiota, which could have ramifications in *F. nucleatum*-related interactions. We therefore treated the mice with *F. nucleatum* (1 × 10^9^ CFU) every two days for four weeks (Supplementary Fig. 2e) and subsequently detected substantial compositional differences, all depleted in *Villin-Cre/Kras*^*G12D+/*^^−^ mice, between the two groups, comprising *Parabacteroides*, *Roseburia*, *Akkermansia*, *Bacteroides*, *Blautia*, *Clostridium XI*, *Anaerostipes*, *Allobaculum*, *Alistipes*, and *Lactobacilus* (Fig. 3a). Next, we used the AOM/DSS mouse model to induce CRC tumorigenesis in the two backgrounds, followed by *F. nucleatum* exposure (Supplementary Fig. 2f). We found that *Bacteroides*, *Parabacteroides*, *Alistipe*s, *Clostridium XI*, and *Intestinimonas* were augmented in WT littermates compared to *Villin-Cre/Kras*^*G12D+/*^^−^ mice (Fig. 3b). Among the four genera (*Parabecteroides*, *Bacteroides*, *Clostridium XI*, and *Alistipe*s) exhibiting concordant alterations in both mouse experiments, *Parabecteroides* manifested *F. nucleatum*-dependent p.G12D depletion with the highest statistical significance, suggesting prominent intermicrobial antagonism.

*Parabacteroides* species are diverse and *P. distasonis*, *Parabacteroides merdae* (*P. merdae*), and *Parabacteroides goldsteinii* (*P. goldsteinii*) have been recognized as the main probiotics conferring protection against CRC and metabolic disorders^17,19,22,23^. To identify which species interacts with *F. nucleatum* and correlates with *KRAS* mutation in CRC development, we examined the abundance of *P. distasonis*, *P. merdae* and *P. goldsteinii* in the colon of AOM/DSS-induced rodents gavaged with *F. nucleatum*. qPCR analysis showed that *P. distasonis*, but not the other two *Parabacteroides* species, registered greater enrichment in WT littermates when compared to *Villin-Cre/Kras*^*G12D+/*^^−^ mice (Fig. 3c). Next, we showed that the inverse association between *F. nucleatum* and *P. distasonis* was found in human tissues with *KRAS* mutations, but absent in *KRAS* WT clinical samples (Fig. 3d, e). By using a logistic regression model having *P. distasonis* as an outcome with *KRAS* and *F. nucleatum*, and their interaction term (*KRAS* x *F. nucleatum*) as exposures, we discovered that *F. nucleatum* could be regarded as a protective factor for *P. distasonis* (OR = 0.548), and the abundance of *P. distasonis* is negatively correlated with *F. nucleatum* (*p* < *0.05*). However, no significant difference was observed between the abundance of *P. distasonis* and *KRAS* mutation (Supplementary Table 2). Overall, our data indicate that *P. distasonis* may antagonize *F. nucleatum* during colorectal tumorigenesis in both mice and humans.

### P. distasonis alleviates F. nucleatum-mediated CRC progression

**Fig. 4:**
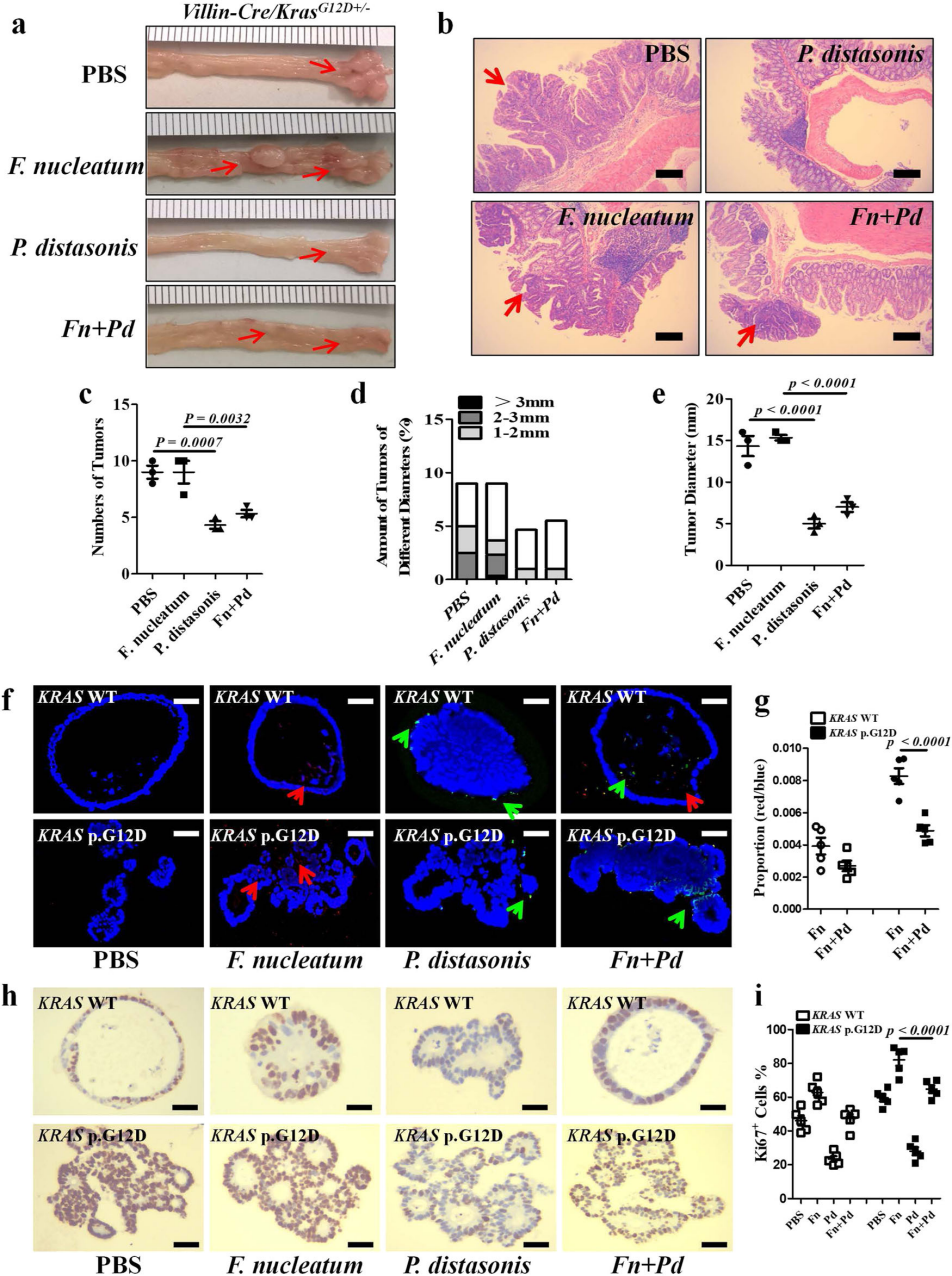
*P. distasonis* alleviates the *F. nucleatum*-mediated CRC progression. **a** Representative images of the colons of *Villin-Cre/Kras*^*G12D+/*^^−^ mice treated with AOM/DSS and *F. nucleatum* or/and *P. distasonis*, *n* = 3 per group. **b** Representative H&E stainings of the colons treated in (**a**), *n* = 3 per group, scale bar: 20 μm. **c**–**e** Tumor numbers, tumor loads, and size of *Villin-Cre/Kras*^*G12D+/*^^−^ mice treated in (**a**). Significant differences are indicated: one-way ANOVA with Bonferroni’s multiple comparison test, *n* = 3 per group, data are presented as the mean ± SEM. **f** FISH detection of *F. nucleatum* (red) and *P. distasonis* (green) in colonic organoids derived from *KRAS* WT and *KRAS* p.G12D CRC patients treated by *F. nucleatum* or/and *P. distasonis*, scale bar: 50 μm. **g** Statistical analysis of the results in (**f**). Significant differences are indicated: two-tailed Student’s *t*-test, *n* = 5 per group, data are presented as the mean ± SEM. **h** Representative immunostainings of Ki67 in colonic organoids derived from *KRAS* WT and *KRAS* p.G12D CRC patients treated by *F. nucleatum* or/and *P. distasonis*, scale bar: 50 μm. **i** Statistical analysis of the results in (**h**). Significant differences are indicated: one-way ANOVA with Bonferroni’s multiple comparison test, *n* = 5 per group, data are presented as the mean ± SEM. Data are representative of two independent experiments. Source data are provided as a Source Data file.

The above findings suggested a possibility that *P. distasonis* could compete with *F. nucleatum* to mitigate CRC development. To verify this hypothesis, we administered AOM/DSS-induced *Villin-Cre/Kras*^*G12D+/*^^−^ mice and WT littermates with *F. nucleatum* and/or *P. distasonis* (Supplementary Fig. 3a). To fully assess the treatment effect of *P. diastonis*, we administrated DSS for three cycles to ensure successful tumor development. Examination of the colon tissues showed that *P. distasonis* gavage alleviated *F. nucleatum*-promoted CRC progression in *Villin-Cre/Kras*^*G12D+/*^^−^ mice (Fig. 4a, b). *P. distasonis*-treated mice developed dramatically fewer and smaller tumors than the *F. nucleatum*- or PBS-treated counterparts; moreover, concurrent exposure to *F. nucleatum* and *P. distasonis* led to diminished size and number of tumors compared to that derived from *F. nucleatum-treated* counterparts (Fig. 4c–e). The biotherapeutic role of *P. distasonis* was corroborated when we treated the clinically derived *KRAS* p.G12D and *KRAS* WT colonic organoids with *F. nucleatum* and/or *P. distasonis* (Fig. 4f, g). In addition, expression of Ki67 in the organoids was significantly decreased in both *P. distasonis*-treated and *P. distasonis*/*F. nucleatum*-co-treated groups, indicating that excessive multiplication of colonic epithelial cells induced by *F. nucleatum* was suppressed in the presence of *P. distasonis* (Fig. 4h and i). Collectively, our data reveal that *P. distasonis* could alleviate *F. nucleatum*-mediated progression of CRC tumors bearing the p.G12D mutation.

### F. nucleatum invades tumor cells and binds to DHX15

**Fig. 5:**
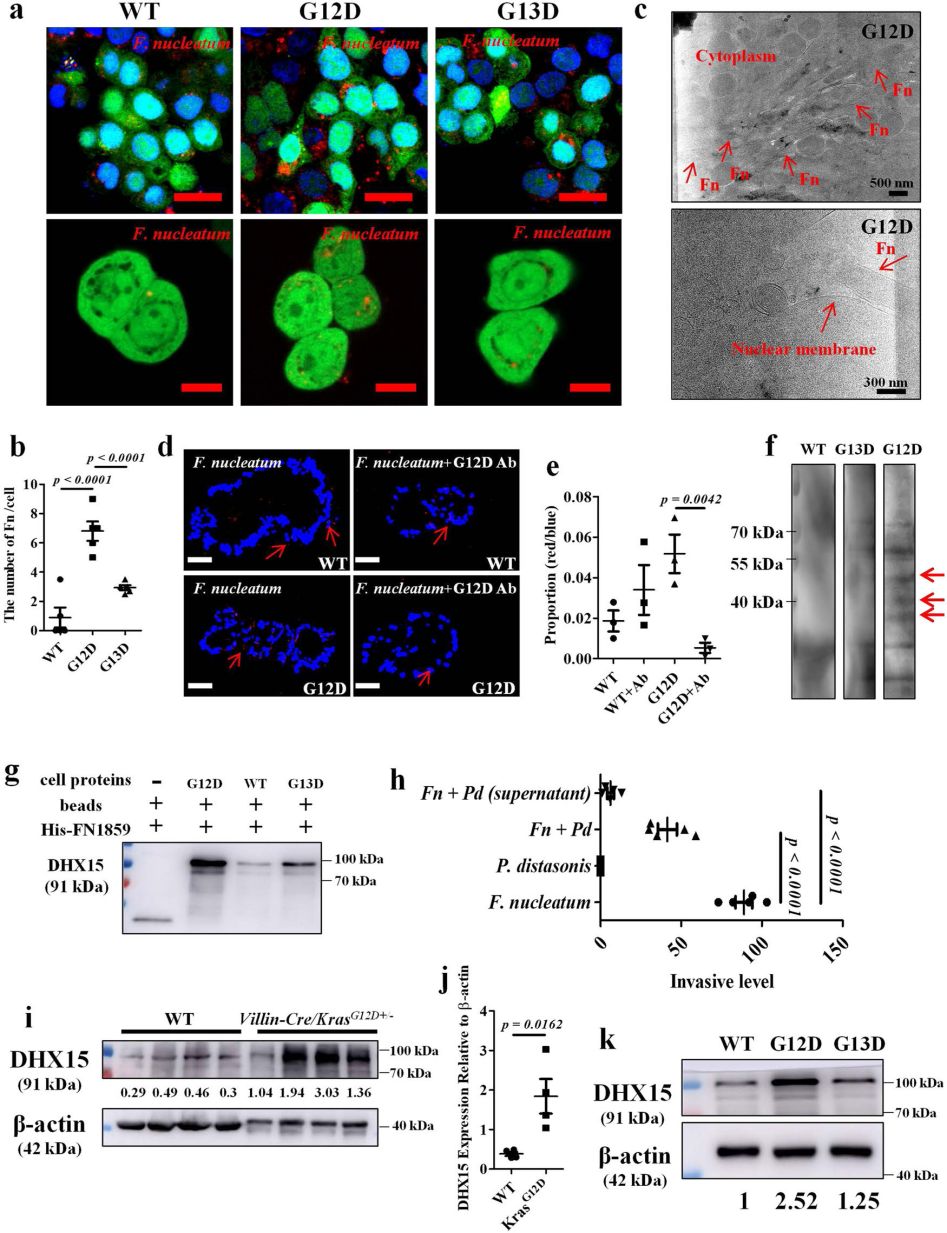
*F. nucleatum* invades tumor cells and binds to DHX15. **a** Representative confocal images of *F. nucleatum* (MOI = 100) invading to indicated tumor cells. The *F. nucleatum* expressed mcherry are in red and tumor cells expressed GFP are in green. Scale bar: 20 μm. **b** Statistical analysis of the results in (**a**). Significant differences are indicated: one-way ANOVA with Bonferroni’s multiple comparison test, *n* = 5 per group, data are presented as the mean ± SEM. **c** *F. nucleatum* was visible inside the G12D cells and contacted the nuclear membrane by cryo-focused ion beam milling and cryo-electron tomography, data are representative of three independent experiments. **d** FISH detection of *F. nucleatum* in colonic organoids derived from indicated patients treated by *F. nucleatum* or *KRAS* p.G12D antibody, scale bar: 50 μm. **e** Statistical analysis of the results in (**d**). Significant differences are indicated: one-way ANOVA with Sidak’s multiple comparison test, *n* = 3 per group, data are presented as the mean ± SEM. **f** Western blot analysis of *F. nucleatum* proteins incubated by biotinylated proteins from indicated cells and detected using avidin-conjugated horseradish peroxidase. The experiment was performed in two biological replicates. **g** Pull-down assays were performed and validation of the FN1859-DHX15 interaction in *KRAS* WT, *KRAS* p.G12D, and *KRAS* p.G13D cells by western blot. The experiment was performed in three biological replicates. **h** The invasive level of *F. nucleatum* when *KRAS* p.G12D cells were incubated with *F. nucleatum, F. nucleatum* + *P. distasonis*, *F. nucleatum* *+* supernatant of *P. distasonis*. Significant differences are indicated: one-way ANOVA with Bonferroni’s multiple comparison test, *n* = 5 per group, data are presented as the mean ± SEM. **i** Western blot analysis of DHX15 expression in *Villin-Cre/Kras*^*G12D+/*^^−^ mice and WT littermates. Lanes representative of separate mice. **j** Statistical analysis of the results in (**i**). Significant differences are indicated: two-tailed Student’s *t*-test, *n* = 4 per group, data are presented as the mean ± SEM. **k** Western blot analysis of DHX15 expression in *KRAS* WT, *KRAS* p.G12D, and *KRAS* p.G13D tumor cells. The experiment was performed in three biological replicates. Source data are provided as a Source Data file.

Given the extensive involvement of *F. nucleatum* in CRC development^13,14^, we assessed whether *F. nucleatum* can invade tumor cells. We first successfully constructed two mutant cell lines expressing *KRAS* p.G12D and p.G13D variants namely *KRAS* p.G12D and p.G13D cells. Western blot analysis confirmed that the *KRAS* p.G12D cells were successfully mutated (Supplementary Fig. 4a). Confocal fluorescence microscopy showed that *F. nucleatum* invaded more in the *KRAS* p.G12D cell line (on an average of 6.8/cell) than in *KRAS* p.G13D (on an average of 2.94/cell) or *KRAS* WT cell line (on an average of 0.9/cell) (Fig. 5a, b), suggesting that the observed *F. nucleatum*-p.G12D link involved a difference in bacterial capacity to penetrate the epithelial cells. Using cryo-focused ion beam milling and cryo-electron tomography, we visualized invasion of *F. nucleatum* inside the cytoplasm of infected G12D cells and confirmed that *F. nucleatum* contacted the nuclear membrane (Fig. 5c). Meanwhile, transmission electron microscope (TEM) experiments were performed on organoids derived from *KRAS* p.G12D-mutant CRC patients and found *F. nucleatum* invaded inside the cytoplasm of the infected organoids (Supplementary Fig. 4b).

The KRAS p.G12D peptide exhibits aberrant activation, which accompanies tumorigenesis^24^. To clarify whether the activation of *KRAS* p.G12D is linked to increased enrichment of *F. nucleatum*, clinically derived *KRAS* p.G12D or WT colonic organoids were infected with *F. nucleatum*, with or without incubation of *KRAS* p.G12D antibody. FISH assay revealed that *F. nucleatum* display elevated invasiveness in the *KRAS* p.G12D-mutant colonic organoids compared to that in the WT organoids, which was attenuated after *KRAS* p.G12D-specific antibody incubation (Fig. 5d, e). To investigate whether the surface proteins on *KRAS* p.G12D mutation that facilitated entry of *F. nucleatum*, we performed western blot to evaluate the expression of tight junction proteins. The reduction of tight junction proteins results in intestinal epithelial barrier dysfunction and bacterial translocation^25^. Results showed that *KRAS* p.G12D mutation cells expressed less ZO-1 and Claudin-1 proteins when compared with *KRAS* p.G13D mutation and *KRAS* WT cells (Supplementary Fig. 4c) which may facilitate the entry of *F. nucleatum*. These data suggested that the p.G12D mutation augments the bacterium-peptide affinity, leading to increased *F. nucleatum* invasion into the tumor cells.

Next, we aimed to identify the cellular component that *F. nucleatum* interacts with. We incubated *F. nucleatum* lysates with biotinylated proteins from *KRAS* p.G12D, *KRAS* p.G13D, and *KRAS* WT cells respectively. Subsequent western blot analysis showed that three candidate proteins from *F. nucleatum* might interact with *KRAS* p.G12D cells (Fig. 5f). The bands of interest were then submitted for mass spectrometry (MS) protein identification, which reported three proteins, FN0488 (glutamate dehydrogenase), FN1277 (aminoacyl-histidine dipeptidase), and FN1859. Among them, FN1859 was the only putative *F. nucleatum* surface protein according to the Uniprot database (https://www.uniprot.org).

FN1859 from *F. nucleatum* might interact with a cognate receptor on *KRAS* p.G12D cell. To capture the unknown effectors, we purified recombinant His-tagged FN1859 proteins from *Escherichia coli* and performed His-tag pull-down assays with WT, *KRAS* p.G13D, and *KRAS* p.G12D cell lysates. Mass spectrometry revealed the level of DHX15 was dramatically augmented in the *KRAS* p.G12D cell line compared with that in the WT and *KRAS* p.G13D cell lines (Supplementary Table 3). DHX15 is an important member of the DEAH-box RNA helicase family that is expressed in the nucleus and contributes to carcinogenesis^26^. To test the possible interaction between FN1859 and DHX15, pull-down assay was conducted in the three cell lines of G12D, G13D, and WT. Western blot analysis demonstrated that DHX15 exhibited specific coupling to FN1859 and that the protein-protein interaction was augmented in *KRAS* p.G12D cells than that in the other two cell lines (Fig. 5g). The similar results were observed in CRC samples from WT, *KRAS* p.G13D and *KRAS* p.G12D-mutant patients, shown in Supplementary Fig. 4e. To clarify whether *P. distasonis* involved in the interaction between *F. nucleatum* and DHX15, TEM was performed to assess the invasive ability of *P. distasonis* to tumor cells. We incubated *P. distasonis* with *KRAS* p.G12D-mutant cells and data showed that no *P. distasonis* was observed in the G12D cells (Supplementary Fig. 4e). Then we purified recombinant His-tagged DHX15 proteins from *Escherichia coli* and performed pull-down assays with *P. distasonis* lysates. There was no protein of *P. distasonis* was pulled by DHX15 (Supplementary Fig. 4f). Therefore, we supposed that *P. distasonis* couldn’t impact the interaction between *F. nucleatum* and DHX15. Furthermore, we investigated whether the invasive level of *F. nucleatum* could be inhibited by *P. distasonis*, we incubated G12D cells with *F. nucleatum, F. nucleatum* + *P. distasonis*, *F. nucleatum* *+* supernatant of *P. distasonis* and carried out the bacterial recovery assays. The results showed that the invasive level of *F. nucleatum* could be inhibited by *P. distasonis*, especially its supernatant implying that the metabolites of *P. distasonis* may involved in inhibiting *F. nucleatum* invasion (Fig. 5h). Finally, DHX15 expression was verified significantly increased in the colon tissues of *Villin-Cre/Kras*^*G12D+/*^^−^ mice compared to WT littermates (Fig. 5i, j), and the pattern was recapitulated in the three cell lines (Fig. 5k). Together, these data reveal that DHX15 is a receptor of *F. nucleatum* in tumor cells and is upregulated upon the p.G12D mutation.

### The ERK/STAT3 signaling mediates the expression of DHX15

**Fig. 6:**
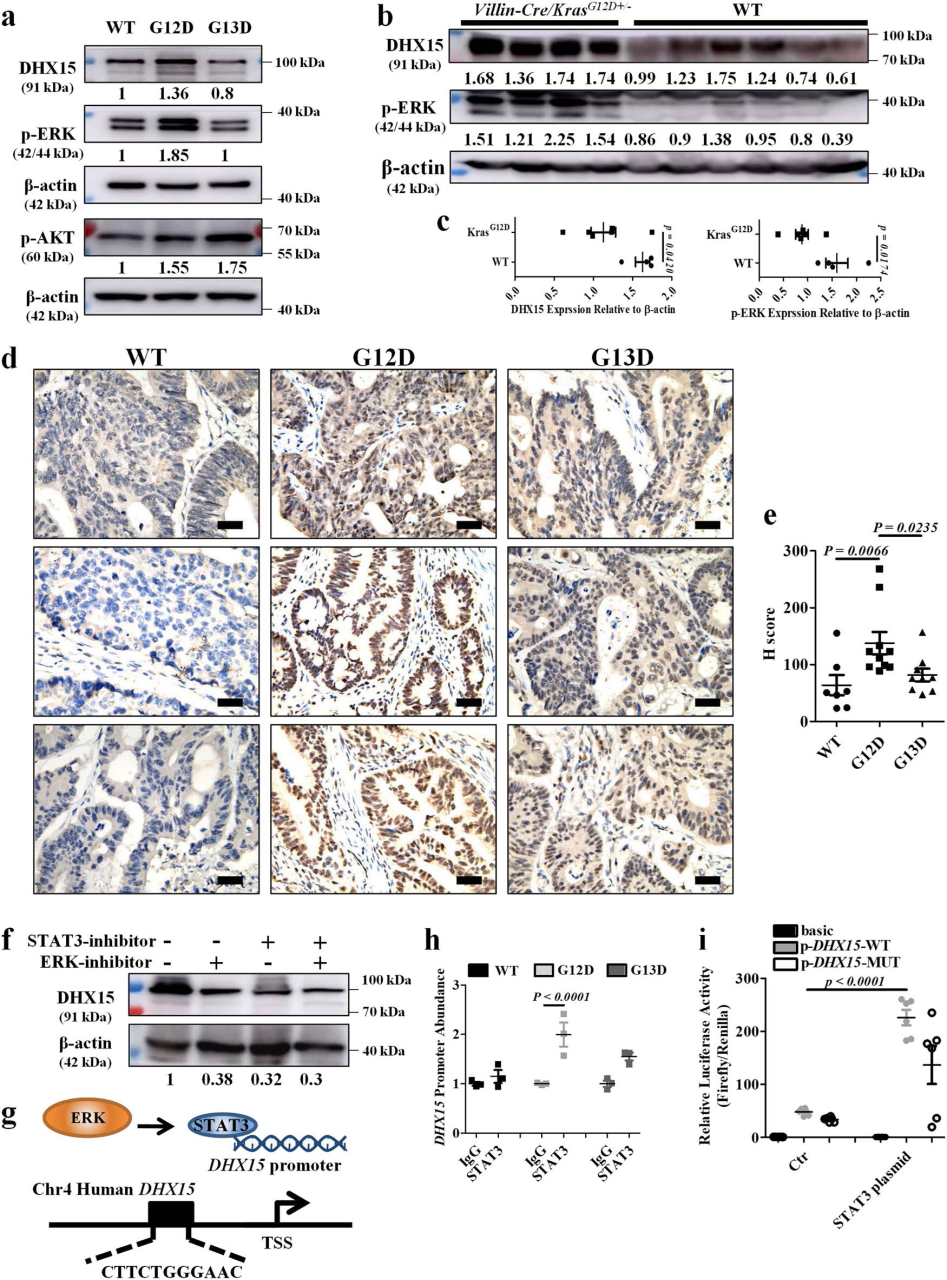
The ERK/STAT3 signaling mediates the expression of DHX15. **a** Western blot analysis of p-ERK, p-AKT, and DHX15 expression in *KRAS* WT, *KRAS* p.G12D, and *KRAS* p.G13D tumor cells. The experiment was performed in three biological replicates. **b** Western blot analysis of p-ERK and DHX15 expression in *Villin-Cre/Kras*^*G12D+/*^^−^ mice and WT littermates. Lanes representative of separate mice. **c** Statistical analysis of the results in (**b**). Significant differences are indicated: two-tailed Student’s *t*-test, *n* = 4 (*Kras*^G12D^) and *n* = 6 (WT) respectively, data are presented as the mean ± SEM. **d** Representative immunohistochemical detection of DHX15 in human CRC tissues of *KRAS* WT, *KRAS* p.G12D, and *KRAS* p.G13D groups, scale bar: 20 μm. **e** Statistical analysis of the results in (**d**). Significant differences are indicated: one-way ANOVA with Sidak’s multiple comparison test, *n* = 7 (WT), *n* = 10 (G12D), and *n* = 9 (G13D) respectively, data are presented as the mean ± SEM. **f** Western blotting analysis of DHX15 expression in *KRAS* p.G12D tumor cells after the specific inhibitor (SCH772987 for ERK and APTSTAT3-9R for STAT3) treatment. The experiment was performed in two biological replicates. **g** The schematic diagram shows one potential binding site of STAT3 in the putative promoter element of DHX15. **h** STAT3 was immunoprecipitated from indicated cells. Immunoprecipitates were assayed for the enrichment of DHX15 promoter. Significant differences are indicated: one-way ANOVA with Bonferroni’s multiple comparison test, *n* = 3 per group, data are presented as the mean ± SEM. **i** Luciferase activity in lysates of 293T cells transfected with luciferase reporter plasmids of pGL3-basic empty vector (basic), *DHX15* promoter (p-*DHX15*-WT) or *DHX15* promoter with mutation on predicted STAT3-binding site (p-*DHX15*-MUT), together with STAT3 plasmid or not. Results are presented as the ratio of firefly luciferase to renilla luciferase activity, relative to that of 293T cells transfected with pGL3-basic empty vector. Significant differences are indicated: one-way ANOVA with Bonferroni’s multiple comparison test, *n* = 6 per group, data are presented as the mean ± SEM. Source data are provided as a Source Data file.

The RAF/MEK/ERK and PI3K/AKT cascades are the major pathways downstream of *KRAS* activation and control the processes of cell growth and survival^27^. To better understand how DHX15 upregulation occurs in *KRAS* p.G12D tumors, we investigated whether these two pathways a re involved in regulating DHX15 expression upon *KRAS* activation. Western blot analysis showed that the level of phosphorylated ERK, but not that of AKT, was substantially upregulated in *KRAS* p.G12D cell line compared with the *KRAS* WT and *KRAS* p.G13D cell lines (Fig. 6a). This coincided with the increased expression of DHX15 in the cells harboring the major *KRAS* mutation (Fig. 6a) as well as augmented DHX15 level and p-ERK activation in the colon tissues of *Villin-Cre/Kras*^*G12D+/*^^−^ mice (Fig. 6b, c). To investigate whether *F. nucleatum* could drive ERK activation and increase DHX15 expression, we performed experiments by co-culture *F. nucleatum* with *KRAS* WT, p.G12D and p.G13D cells and found that the level of phosphorylated ERK and DHX15 in the *F. nucleatum*-treated cells was the same with untreated cells (Supplementary Fig. 5a, b). To analyze the level of DHX15 in patient samples, we performed IHC on tumor tissues of patients with CRC that harbor *KRAS* p.G12D, p.G13D mutations, and *KRAS* WT allele. We found that *KRAS* p.G12D-bearing CRC expressed a greater level of DHX15 proteins compared with *KRAS* p.G13D-bearing and *KRAS* WT-bearing patients (Fig. 6d, e), which collectively illustrated the functional connections of the putative RNA helicase within the p.G12D-bearing tumors.

ERK is located in the cytoplasm and upon activation is transferred to the nucleus to regulate the activity of various transcription factors such as FOS, ELK1, MYC, and STAT1/3^28^. To predict the putative downstream transcription factor of ERK that could directly initiate the transcription of DHX15, the bioinformatics tool JASPAR was used to identify STAT3. Correspondingly, the specific ERK inhibitor and STAT3 inhibitor were employed to examine the effect of this pathway on DHX15 expression and detected a significant downregulation at the protein level (Fig. 6f). These data indicate that the ERK-STAT3 signaling activation is required for DHX15 upregulation.

Curation of protein database identified a potential STAT3-binding site in the promoter of DHX15 (Fig. 6g). Chromatin immunoprecipitation (ChIP) assays showed that DHX15 promoter abundance was increased by 2.0-fold in *KRAS* p.G12D cells, 1.15-fold in *KRAS* WT cells, and 1.54-fold in *KRAS* p.G13D cells (Fig. 6h). Luciferase reporter assay verified that STAT3 bound to the promoter element of DHX15 and affected DHX15 expression (Fig. 6i). Taken together, our data demonstrate that the activation of ERK/STAT3 signaling in the downstream of KRAS is involved in DHX15 upregulation.

### Knock out of Dhx15 in Villin-Cre/Kras G12D^+/−^ mice attenuates the CRC phenotype

**Fig. 7:**
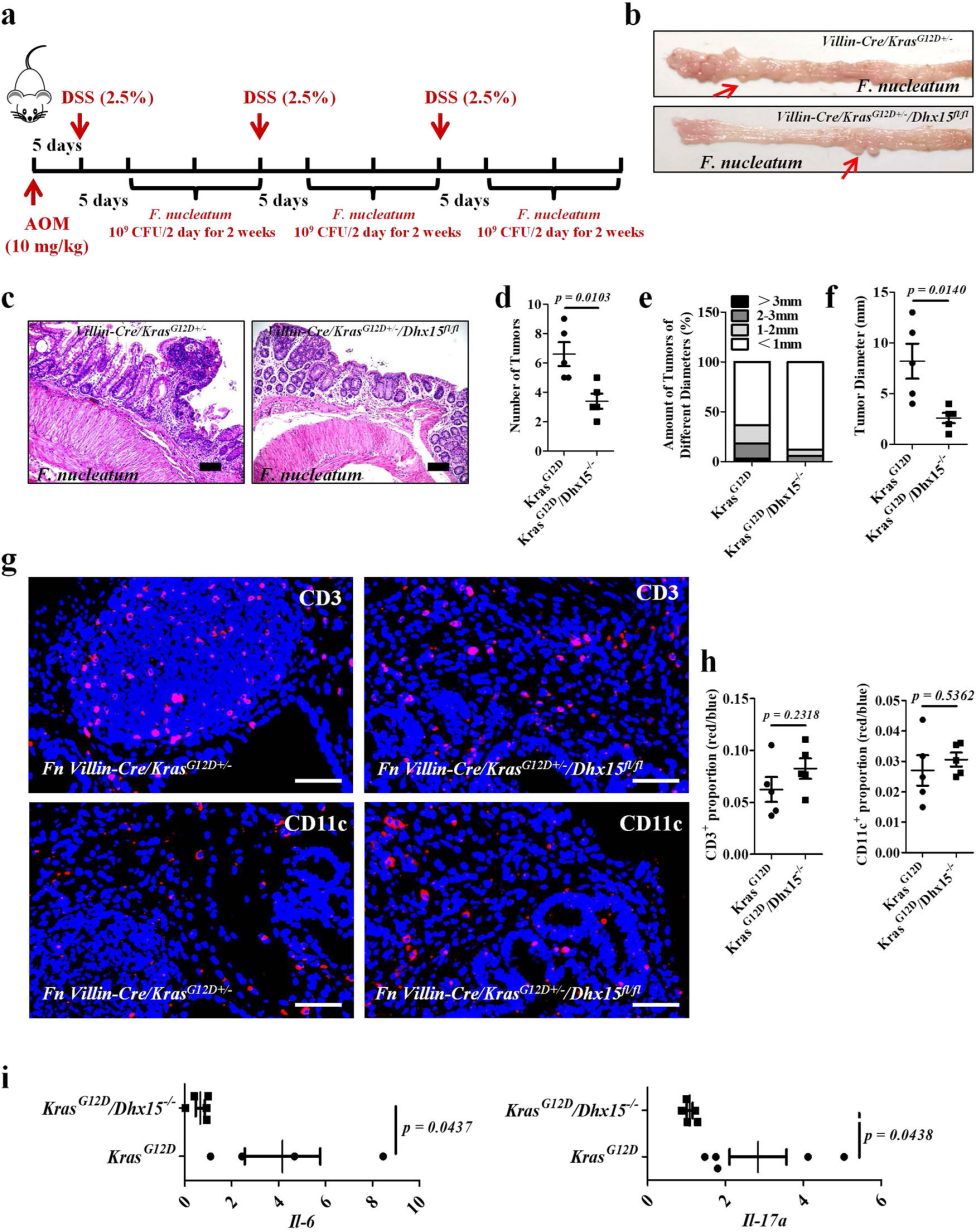
Knock out *Dhx15* in *Villin-Cre/Kras*^*G12D+/*^^−^ mice attenuated the CRC phenotype. **a** Schematic diagram of the experimental design and timeline of mouse models. **b**, **c** Representative images and H&E stainings of the colons of *Villin-Cre/Kras*^*G12D+/*^^−^ mice and *Villin-Cre/Kras*^*G12D+/*^^−^*/Dhx15*^*fl/fl*^ mice treated with AOM/DSS and *F. nucleatum*, the red arrows indicate tumors, *n* = 5 per group, scale bar: 50 μm. **d**–**f** Tumor numbers, tumor loads, and size of *Villin-Cre/Kras*^*G12D+/*^^−^ mice and *Villin-Cre/Kras*^*G12D+/*^^−^*/Dhx15*^*fl/fl*^ mice treated with AOM/DSS and *F. nucleatum*. Significant differences are indicated: two-tailed Student’s *t*-test, *n* = 5 per group,data are presented as the mean ± SEM. **g** Representative immunofluorescence detection of CD3 and CD11c positive cells in *Villin-Cre/Kras*^*G12D+/*^^−^ mice and *Villin-Cre/Kras*^*G12D+/*^^−^*/Dhx15*^*fl/fl*^ mice treated with AOM/DSS and *F. nucleatum*, scale bar: 50 μm. **h** Statistical analysis of the results in (**g**). Significant differences are indicated: two-tailed Student’s *t*-test, *n* = 5 per group, data are presented as the mean ± SEM. **i** qPCR analysis of *Il-17a*, *Il-6* mRNA expression *Villin-Cre/Kras*^*G12D+/*^^−^ mice and *Villin-Cre/Kras*^*G12D+/*^^−^*/Dhx15*^*fl/fl*^ mice treated with AOM/DSS and *F. nucleatum*. Significant differences are indicated: two-tailed Student’s *t*-test, *n* = 4 (*Kras*^*G12D*^) and *n* = 5 (*Kras*^G12D^/*Dhx15*^−^^/^^−^) respectively, data are presented as the mean ± SEM. Data are representative of two independent experiments. Source data are provided as a Source Data file.

To verify the regulatory effect of DHX15 in colorectal tumorigenesis, we knocked out DHX15 in *Villin-Cre/Kras*^*G12D+/*^^−^ *mice* by generating *Villin-Cre/Kras*^*G12D+/*^^−^*/Dhx15*^*fl/fl*^ mice (Supplementary Fig. 6a). Then we developed an AOM/DSS-induced CRC mouse model and gavaged *F. nucleatum* in both *Villin-Cre/Kras*^*G12D+/*^^−^ and *Villin-Cre/Kras*^*G12D+/*^^−^*/Dhx15*^*fl/fl*^ mice (Fig. 7a). We verified that *F. nucleatum*-treated *Villin-Cre/Kras*^*G12D+/*^^−^*/Dhx15*^*fl/fl*^ mice exhibited a relieve in tumor formation compared with *F. nucleatum*-treated *Villin-Cre/Kras*^*G12D+/*^^−^ mice (Fig. 7b). H&E staining confirmed that *F. nucleatum*-treated *Villin-Cre/Kras*^*G12D+/*^^−^*/Dhx15*^*fl/fl*^ mice showed lower grades of dysplasia (Fig. 7c), tumor multiplicities and tumor loads than *F. nucleatum*-treated *Villin-Cre/Kras*^*G12D+/*^^−^ mice (Fig. 7d–f). To investigate whether immune factors involved in the pro-tumorigenic activities of DHX15-*F. nucleatum* interaction, IF, and qPCR were performed to evaluate the infiltrated immune cells and inflammatory cytokine production in colonic tissues of *F. nucleatum*-treated *Villin-Cre/Kras*^*G12D+/*^^−^ and *Villin-Cre/Kras*^*G12D+/*^^−^*/Dhx15*^*fl/fl*^ mice. We found that there were no significant differences in the number of infiltrated CD3^+^ T cells and CD11c^+^ dendritic cells between the two groups (Fig. 7g and 7h). However, the expression of *Il-6*, *Il-17a* were elevated after *F. nucleatum* treatment in *Villin-Cre/Kras*^*G12D+/*^^−^ mice when compared to *Villin-Cre/Kras*^*G12D+/*^^−^*/Dhx15*^*fl/fl*^ mice (Fig. 7i) suggested that the inflammatory cytokines might contribute to the pro-tumorigenic activities of *F. nucleatum*. Overall, we verified that the *F. nucleatum*-mediated-colorectal tumorigenesis is attenuated in mice activating *Kras* p.G12D and lacking DHX15.

## Discussion

Colorectal cancer is characterized by gut microbiota abnormalities and specific somatic mutations. Recent studies revealed that high *F. nucleatum* abundance in the tumor tissues was significantly associated with *BRAF* mutations and CpG island methylator phenotype (CIMP)-positive CRC patients in univariate analyses and the amount of *F. nucleatum* was also associated with microsatellite instable (MSI)-high tumors independent of CIMP and *BRAF* mutation status, supporting the notion that gut microbiota has links to the intratumor genetics and epigenetics of CRC^29–31^. Our study presents a greater complexity of niche elements converged in the *F. nucleatum*-dependent CRC tumorigenesis, as the functional links comprise a somatic mutation, a putative host RNA helicase, a signaling pathway crucial for multiple malignancies, and a *F. nucleatum*-antagonizing bacterium. The findings indicate that a comprehensive approach is needed to disentangle the connection between the gut microbiota shift and CRC development.

Considerable effort has been devoted to understand and manage *KRAS* mutant-mediated therapeutic resistance. The biophysical differences between individual *KRAS* mutant alleles including the p.G12D, p.G12V and p.G13D variants are sufficient to generate a range of sensitivities to EGFR inhibitor^32^. Previous evidence has shown that in both early-onset and later-onset tumors, *KRAS* mutation prevalence was higher in the cecum compared with that in the other subsites^33^. Among CRCs in all colorectal sites, cecal cancers show the highest *KRAS* mutation frequency^34^. Therefore, cecal cancers appeared to represent a unique subtype, characterized by a high frequency of *KRAS* mutation^35^. Interestingly, *F. nucleatum* is most abundant in cecal cancers^36^ which support our findings in this study that patients harboring *KRAS* p.G12D mutation are correlated with an elevated *F. nucleatum* presence. In fact, the relationship between *F. nucleatumn* and *KRAS* mutations is not conclusive based on the previous studies. Kosuk Mima et al. have observed 1069 CRC samples and found that the amount of *F. nucleatum* was not associated with *KRAS* mutation^30^. Ivan Borozan et al. investigated 1,994 CRC cases and concluded that mutation status of *APC*, *PIK3CA*, *KRAS*, *BRAF*, *ERBB2*, and *SMAD4* were not associated with *F. nucleatum* prevalence^31^. In the two studies above, the genomic DNA of *F nucleatum* was extracted from FFPE tissue sections of CRC. However, in another research, *KRAS* mutation was found to be more frequently observed in CRC samples infected with *F. nucleatum* and the genomic DNA of *F nucleatum* was extracted from 43 fresh-frozen CRC tissue samples and the matched adjacent normal tissues^37^. Consistent with this, we evaluated 24 fresh-frozen CRC tissue samples and the matched adjacent normal tissues and 239 fresh-frozen CRC tumor tissues and verified that *F. nucleatum* abundance was associated with *KRAS* mutation. The potential differences in different researches maybe due to the tissue status (fresh frozen vs. FFPE). What’s more, here we identified that *KRAS* genotype affects the activation of downstream ERK signaling pathway and *KRAS* p.G12D mutation-induced activation of ERK signaling is stronger than *KRAS* p.G13D and *KRAS* WT which is similar with a previous report that *BRAF* V600E could induce stronger ERK activity than *KRAS* p.G12V mutation^38^.

The activation of ERK signaling leads to expression of DHX15 which is a member of the DEAH-box RNA helicase family and is required for virus-induced innate immune gene expression^39,40^. Studies have reported that DHX15 contributes to carcinogenesis in several malignancies including prostate cancer, non-small cell lung cancer, and hepatocellular carcinoma^26,41,42^. However, a functional tie with disease-related gut microbes remains poorly characterized, although it has recently been reported that DHX15 deficient mice are susceptible to infection by enteric bacteria *Citrobacter rodentium* and that the protein plays an important role in the antimicrobial response in colitis^43^. Here we show that the elevated expression of DHX15 is pertinent to *KRAS* p.G12D mutation and DHX15 as a specific receptor in the nuclei of tumor cells interact with *F. nucleatum* to mediated-colorectal tumorigenesis.

*P. distasonis* negatively correlates with several adverse health conditions including inflammatory bowel disease, multiple sclerosis, and obesity^19,44^, which coincides with its protective role against intestinal epithelial damage and colonic tumorigenesis in mice^45^. In this study, we show a competition between *P. distasonis* and *F. nucleatum* that is more pronounced in p.G12D-bearing mice and patients than their counterparts with the WT allele. These findings collectively raise the possibility of developing the bacterium as probiotics or auxiliary therapeutics for colorectal cancer, although more in-depth exploration is needed. Recently, combination of KRAS inhibitor AMG510 and anti-PD-1 monotherapy delayed tumor growth, with complete regression in nine of ten tumors and generated most attention^46^. Whether the combination of microbiota such as *P. distasonis* and anti-PD-1 monotherapy could represent a potentially trans-formative therapy for patients for whom effective treatments are lacking also deserves further investigation.

Taken together, we propose a pathogenic model of CRC dependent on somatic genotype. In *KRAS* p.G12D mutation CRC tissues, *F. nucleatum* invades more and the ERK signaling is activated and subsequently induces DHX15 expression. The interaction of FN1859-DHX15 potentiates colorectal tumorigenesis and could be alleviated by *P. distasonis*. Our findings may provide a basis for personalized therapy for the subset of treatment-recalcitrant CRC patients carrying the *KRAS* p.G12D mutation.

## Methods

### Mice

*Villin-Cre/Kras*^*G12D+/*^^−^ mice and *Villin-Cre/Kras*^*G12D*^^−^^*/*^^−^ littermates constructed in C57BL/6J background were purchased from The Shanghai Model Organisms Center. Mice were bred and maintained under specific pathogen-free (SPF) conditions. Age- and sex-matched mice at 6-8 weeks of age and mixed mice were randomly used for all experiments. According to the guidelines (GB/T 35892-2018), the ethics committee specified that the maximal tumor burden is no more than 10% of the body weight of animals and the average diameter is less than 20 mm. During the experiment, the tumor sizes of the mice complied with the regulations. All animal protocols were reviewed and approved by the Ethics Review Committee for Animal Experimentation at Shanghai Tenth People’s Hospital.

### Human subjects

The primary tumor tissues and the adjacent normal tissues were obtained from patients with colorectal adenocarcinoma who underwent a surgical resection. 254 fresh tumor tissues and 24 adjacent normal tissues were collected at the time of surgical resection and were immediately frozen in liquid nitrogen and stored at −80 °C. Additionally, formalin-fixed, paraffin-embedded specimens (one from each case) were collected from Department of Pathology of Shanghai Tenth People’s Hospital Affiliated to Tongji University. According to the TNM staging system, tumors were classified independently by two pathologists. The clinic pathological parameters included age, sex, tumor size, tumor location, pathological grade, and tumor size. All individuals provided informed consent and the ethics approval also covered the “Establishment of patient-derived organoids”. The study was performed in accordance with the Declaration of Helsinki Principles and approved by the Research Ethics Board of Shanghai Tenth People’s Hospital, Tongji University School of Medicine (SHDSYY-2019-2751).

### 16S rRNA sequencing analysis

Colon tissues from patients or mice were collected. The total DNA was then extracted with the QIAamp DNA Mini kit (QIAGEN). The 16S rRNA high-throughput sequencing was performed by Realbio Genomics Institute (Shanghai, China) using the Illumina MiSeq. Variable regions V3-V4 on 16S rRNA genes of bacteria were amplified with forward primer F341 5′-ACTCCTACGGGRSGCAGCAG-3′ and reverse primer R806 5′-GGACTACVVGGGTATCTAATC-3′. The raw data were then subjected to a quality control procedure using UPARSE. The qualified reads were clustered to generate operational taxonomic units (OTUs) at the 97% similarity level using Usearch. Principal components analysis (PCA), heatmap analysis, Bray-Curtis similarity cluster, and species abundance analysis were performed.

### Targeted gene sequencing

Five genes were screened for the detection of mutations in patients with CRC by sequencing using the Illumina NovaSeq 6000. To identify the mutations in these genes, we designed PCR primers using the primerXL pipeline. Three hundred and eighty oligonucleotide pairs were produced and encompassed all of the CDSs and most of the untranslated regions of the 5 genes. The amplification reactions were conducted using an AB 2720 Thermal Cycler (Life Technologies Corporation) with the following cycling conditions: 95 °C for 2 min; 11 cycles of 94 °C for 20 s, 63 °C per cycle for 40 s, 72 °C for 1 min; 24 cycles of 94 °C for 20 s, 65 °C for 30 s, 72 °C for 1 min and 72 °C for 2 min. The PCR products were used generate a library for further detection, and the DNA-adapter-ligated and -indexed fragments from ten libraries were then pooled and hybridized. After hybridization of the sequencing primer, base incorporation was performed using the Illumina NovaSeq 6000 in a single lane following the manufacturer’s standard cluster generation and sequencing protocols for 250 cycles of sequencing per read to generate paired-end reads, including 250 bp at each end and 8 bp of the index tag. The primer sequences are provided in Source Data file.

### Animal experiments

Following the single dose of AOM (10 mg/kg, intraperitoneal) injection, mice were treated 2.5% DSS (molecular weight 36,000–50,000, MP biomedicals) in drinking water for 5 successive days (To investigate whether *F. nucleatum* exacerbates colorectal tumorigenesis in *Villin-Cre/Kras*^*G12D+/*^^−^ mice in early time, mice were administrated DSS for one cycle. To assess the treatment effect of *P. diastonis* and the role of DHX15 respectively, mice were administrated DSS for three cycles to ensure successful tumor development.) and then gavaged with 1 × 10^9^ CFU of *F. nucleatum* and/or *P. distasonis* every 2 days for 4 weeks. The animals in the control group were gavaged by the same volume of PBS. Then mice were sacrificed to harvest colon tissues for analysis.

### Bacterial recovery assay

1 × 10^5^ cells were grown in a 24-well plate and co-cultured with bacteria for 1 h (MOI = 100) under anaerobic conditions. After co-culture, Ampicillin (200 mg/ml) and Gentamicin (200 mg/ml) were used to eliminate extracellular bacteria for 1 h, and medium was removed and cells were washed with PBS three times. To lyses the cells, 100 μl of H_2_O was added for 20 min, followed by the addition of 900 μl of Wilkins–Chalgren anaerobe broth to homogenize the cells. The invaded *F. nucleatum* colonies were recovered on Wilkins–Chalgren anaerobe agar plate under anaerobic conditions; the number of colonies was counted.

### FISH

Frozen colon tissues were fixed in Carnoy’s solution overnight and embedded in paraffin; 5 μm thick sections were hybridized in the hybridization buffer (0.9 M NaCl, 20 mM Tris/HCl, pH 7.3, 0.01% SDS). Stringency was used with the form amide concentration from 0 to 30% (v/v). Pre-warmed hybridization buffer (20 ml) was mixed with approximately 5 pmol of the oligonucleotide probe and carefully applied to the tissue sections. After incubation for 5 h in a dark humid chamber at 46 °C, each of the slides were rinsed with sterile double-distilled water, air-dried in the dark, and mounted with ProLong Gold Antifade Mountant with DAPI (Thermo Fisher Scientific). The probe sequences used to detect *F. nucleatum* are listed as followed: 5′-CGCAATACAGAGTTGAGCCCTGC-3′^47^.

### DNA extraction and qPCR

Mucosal tissues were digested in PBS containing an enzymatic cocktail of mutanolysin (250 U/ml) and lysozyme (1 mg/ml) (Sigma-Aldrich) at 37 °C for 1 h, total genomic DNA was extracted with the QIAamp DNA Mini Kit (QIAGEN) according to the manufacturer’s instructions. qPCR was performed to detect the *F. nucleatum* or *P. distasonis* level by using 40 ng genomic DNA in 20 μl universal SYBR Green PCR Master Mix (Roche) in a ViiA 7 Real-Time PCR System (Applied Biosystems). *F. nucleatum* or *P. distasonis* quantitation was measured relative to the pgt gene. The primers used are listed as followed: *F. nucleatum* forward: CAACCATTACTTTAACTCTACCATGTTCA, *F. nucleatum* reverse: GTTGACTTTACAGAAGGAGATTATGTAAAAATC, *P. distasonis* forward: CCACGCAGTAAACGATGA, *P. distasonis* reverse: 5′-CTTAACGCTTTCGCTGTG-3′, prostaglandin transporter (pgt) forward: 5′-ATCCCCAAAGCACCTGGTTT-3′, pgt reverse: 5′-AGAGGCCAAGATAGTCCTGGTAA-3′.

### Cell culture and stable cell lines construction

The human colon cancer cell line HT-29 was obtained from Stem Cell Bank, Chinese Academy of Sciences, and was maintained in Dulbecco’s modified Eagle’s medium (DMEM) (Hyclone, #SH30243.01) supplemented with 10% FBS (Gibco, #10270-106), 1% penicillin/streptomycin (Beyotime, #C0222). All cells were cultured at 37 °C supplied with 5% CO_2_. The cell lines with stable overexpression of *KRAS* p.G12D, p.G13D, or control cells were generated by infection HT-29 cells with *KRAS* G12D-sgRNA-Cas-EGFP, *KRAS* G13D-sgRNA-Cas-EGFP and negative scramble control-EGFP lentiviral plasmids which were purchased from GeneChem (Shanghai, China) according to the manufacturer’s instructions.

### Establishment of patient-derived organoids (PDOs)

Biopsies from CRC patients were collected in 5 ml PBS containing penicillin/streptomycin on ice. Following washing and mincing tissues into around 1–2 mm^3^, samples were digested with 10 ml of cell dissociation reagent (Stem Cell, #07174) on ice on a rocking platform for 30 min. Dissociated cells were passed through 100 μm cell strainer, and then pelleted and suspended in ice-cold PBS. Centrifuge cells for 300 g, 5 min, and then resuspend cells in growth factor reduced (GFR) matrigel (Corning, #356231), and seed cells on 48-well cell culture plate (Corning, #3548). Following solidified in 37 °C and 5% CO_2_ incubator for 30 min, 300 μl of human IntestiCult™ Organoid Growth Medium (Stem Cell, #06010) which were additionally added 10 μM of Y27632 (Stem Cell, #72304) for the primary culture and were overlaid in the well coated with matrigel. As for the passaging of PDOs, organoids were harvested with ice-cold PBS and pipetted with mechanical force through 1 ml pipette (160 times per well). Dissociated PDOs were pipetted and washed with ice-cold PBS. Resuspend the dissociated cells in GFR matrigel and re-seeded on 48-well flat bottom cell culture plate. PDOs were frozen in FBS containing 10% DMSO for biobanking. The information of the patients from whom the organoids derived is provided in Supplementary Table 1.

### Far-western assay

The far-western assay was performed as the reference^48^. To prepare the biotinylated cell proteins, cell proteins were extracted with PBS containing 1% Triton X-100 (Sigma-Aldrich) for 1 h at room temperature, then labeled with 1 mM EZ-Link Sulfo-NHS-LC-Biotin (Thermo Fisher Scientific) for 2 h at 4 °C. Whole *F. nucleatum* proteins were extracted with PBS containing 1% Triton X-100, separated by SDS–polyacrylamide gel electrophoresis (PAGE) and transferred onto a polyvinylidene difluoride (PVDF) membrane. The PVDF membrane was blocked with 5% BSA for 1 h and incubated with biotinylated cell proteins overnight at 4 °C. Biotin-labeled proteins were detected using avidin-conjugated horseradish peroxidase (HRP). The corresponding bands in SDS-PAGE were excised for identification by mass spectrometry.

### His pull-down assay

The recombinant His-FN1859 or His-DHX15 was produced in *Escherichia coli* strains. To perform the His pull-down assay, His-FN1859 or His-DHX15 was incubated with Ni-NTA Magnetic Beads, followed by adding cell or bacteria proteins indicated overnight at 4 °C. The beads were then washed and boiled with SDS-PAGE gel loading buffer. Eluted proteins were separated by SDS-PAGE and analyzed by mass spectrometry.

### Mass spectrometry

Scoop out the area where the protein is located in SDS-PAGE and cut it into small pieces about 1 mm^3^. Add double steaming water to soak the glue block, shake for 10 minutes, absorb the lotion. The glue block was immersed in 50% ACN/100 mM NH_4_HCO_3_ (pH 8.0) solution, shook for 10 min, and the lotion was absorbed. The process was repeated three times. Add 100% ACN-impregnated glue block, shake for 10 min, absorb the lotion, and then drain the glue block in a vacuum draining machine. 10 mM DTT/50 mM NH_4_HCO_3_ (pH 8.0) solution was added to the glue block, incubated at 56 °C for 1 h for reduction reaction, and then the leaching solution was removed. After that, 55 mM iodoacetamide/50 mM NH_4_HCO_3_ (pH 8.0) solution was added, and the solution was incubated at room temperature and dark place for 30 min. Add 100% ACN, shake for 20 min, absorb the infusion, drain the glue block. Appropriate amount of trypsin was added to the glue blocks, and 50 mM NH_4_HCO_3_ solution was added to completely cover the glue blocks, and the glue blocks were incubated at 37 °C overnight for enzyme digestion. Then 60% ACN/5% formic acid was added, ultrasonic shock was performed for 10 min, and the supernatant was absorbed into the new centrifuge tube after centrifugation. After the extraction process is repeated twice, the extracted liquid is combined and drained in the centrifugal concentrator. The peptide was desalted using C18 column and frozen at −20 °C for machine detection.

### Chromatin immunoprecipitation assays

Chromatin immunoprecipitation (ChIP) assays were performed using the SimpleCHIP enzymatic chromatin immunoprecipitation kit (Cell Signaling Technology, #9002) according to the manufacturer’s protocol with minor modifications. The genomic DNA recovered from the ChIP assays was amplified with primers specific to the STAT3-binding elements of the DHX15 promoter region. The specificity of the primer set was verified by analyzing the dissociation curve of each gene-specific PCR product and listed in Supplementary Table 4.

### Luciferase reporter assays

PGL3-basic vector (Promega, #E1751) was used to clone the promoter of DHX15. Site-specific mutant was generated by PCR. 293T cells were provided from the cell bank of Chinese Academy of Sciences and seeded in a 96-well plate with a density of 0.8 × 10^5^/well 1 day before transfection. Wells was transfected with a mixture of 100 ng pGL3 luciferase vector, DHX15 WT plasmid or DHX15 mutant plasmid or/and 100 ng STAT3 plasmid and 25 ng pRL-TK renilla vector using Lipofectamine 3000 Transfection Reagent (Invitrogen, #L3000-15). Twelve hours post-transfection, luciferase activity was measured on a microplate reader (Berthold, TriStar LB941) by using the Dual-Luciferase Reporter Assay System (Promega, #2920). The ratio of firefly luciferase to renilla luciferase was calculated for each well.

### RNA extraction, reverse transcription, and qPCR

Total RNA was extracted from colon tissues from mice using the TRIzol reagent (Invitrogen, #15596-026), and NanoDrop spectrophotometer (ND-1000) was used for RNA quality control. cDNA was synthesized using PrimeScript^TM^ RT Master Mix (TaKaRa, #RR036A). qPCR was carried out with the TB Green Premix Ex Taq^TM^ II (TaKaRa, #RR820A) in a ViiA 7 Real-Time PCR System (Applied Biosystems). The relative expression of target genes was confirmed using quantity of target gene/quantity of β-actin. Primer sequences are listed in Supplementary Table 4.

### Western blotting

Mouse colons or cultured cells were lysed in radio immunoprecipitation assay buffer supplemented with protease and phosphatase inhibitor cocktail (Thermo Scientific, #78440). Antibody used are listed in Supplementary Table 5. The signal was detected with ECL Western Blotting Substrate (Thermo Scientific, #34095) and Amersham Imager 600 (GE Healthcare). Images have been cropped for presentation.

### Immunohistochemistry and immunofluorescence

The human or mouse colons were fixed in formalin and embedded in paraffin. Sections (6 μm) were stained with hematoxylin and eosin (H&E). For immunohistochemistry, Ki67 or DHX15 expression was evaluated in colon sections using rabbit anti-Ki67 Ab (1:100 dilution, Cell Signaling Technology, #12202) or rabbit anti-DHX15 Ab (1:100 dilution, Proteintech, #12265-1-AP), following the manufacturer’s instructions. For immunofluorescence, CD3 or CD11c expression was evaluated in colon sections using rat anti-CD3 Ab (1:100 dilution, Abcam, #ab11089) or mouse anti-CD11c Ab (1:100 dilution, Abcam, #ab254183), following the manufacturer’s instructions. For observation *F. nucleatum* invasion, cells were seeded in 35 mm plates and exposed to *F. nucleatum* with a MOI of 100 under anaerobic conditions. Then the cells were washed for three times and imaged immediately or fixed in 4% formaldehyde containing 0.1% Triton X-100 at room temperature for 15 min. DAPI was used to visualize the nuclei. Images were obtained by Olympus BX51 microscope or Zeiss LSM900 confocal microscope.

### Bacterial strains and culture conditions

*F. nucleatum* (25586) was purchased from ATCC and *P. distasonis* was isolated from CRC patients and has a 99.72% identity with the 16S rRNA gene sequence of *Parabacteroides distasonis* strain ATCC 8503 (NCBI accession number NR_074376.1) (Supplementary Table 6). Identification of cultured *F. nucleatum* and *P. distosonis* by Nanopore Sequencing is shown in Supplementary Table 7. The strains were maintained in bottled Thioglycollate anaerobe broth (Sanyao, #15611) or Columbia agar in an anaerobic chamber (5% CO_2_, 2% H_2_, and 93% N_2_) at 37 °C.

### Transmission electron microscope

Cells were harvested after trypsin digestion, and fixed with 2.5% glutaraldehyde solution. PDOs were dissociated by TrypLETM Express (Gibco, 12604-013), and fixed in 2.5% glutaraldehyde solution. TEM (Hitachi) was operated at a voltage of 80 kV and equipped for morphological observation after sample preparation.

### Statistics and reproducibility

Each experiment was performed at least two biological replicates. The data were analyzed with Graphpad Prism 5 and Spss 22.0. Acquired data were presented as mean values, and error bars represent the SEM. Student’s *t*-test was used when two conditions were compared, and one-way ANOVA with Bonferroni post-test or Sidakʼs post-test was used for multiple comparisons. The correlations between *F. nucleatum*, *P. distasonis* abundance and *KRAS* mutation status were analyzed using Chi-square. The probability values of <0.05 were considered statistically significant. Exact *p-value* was provided.

### Reporting summary

Further information on research design is available in the Nature Portfolio Reporting Summary linked to this article.

### Supplementary information

Supplementary Information

Peer Review File

Reporting Summary

### Source data

Source Data

## Data Availability

The sequencing data in this study have been deposited in the GSA with accession number CRA013275, CRA013274, CRA013276, CRA013455, HRA006025. Dataset HRA006025 is available under restricted access for human genetics data privacy concerns; access can be obtained by the DAC (Data Access Committees) of the GSA-human database. The approximate response time for accession requests is about 3 days. Once access has been approved, the data will be available for 3 months. The user can also contact the corresponding author directly. The mass spectrometry proteomics data have been deposited to the ProteomeXchange Consortium via the PRIDE partner repository with the dataset identifier PXD048684, PXD048686. The remaining data are available within the Article, Supplementary Information or Source Data file. Source data are provided as a Source Data file. Source data are provided with this paper.
