# Supplementary material for: Fusobacterium nucleatum promotes tumor progression in KRAS p.G12D-mutant colorectal cancer by binding to DHX15
Source: Nat Commun. 2024 Feb 24;15:1688. doi: 10.1038/s41467-024-45572-w (PMC10894276; doi:10.1038/s41467-024-45572-w)
Supplement: Supplementary file 1 — Supplementary Information [file 41467_2024_45572_MOESM1_ESM.pdf]

Supplementary Figures

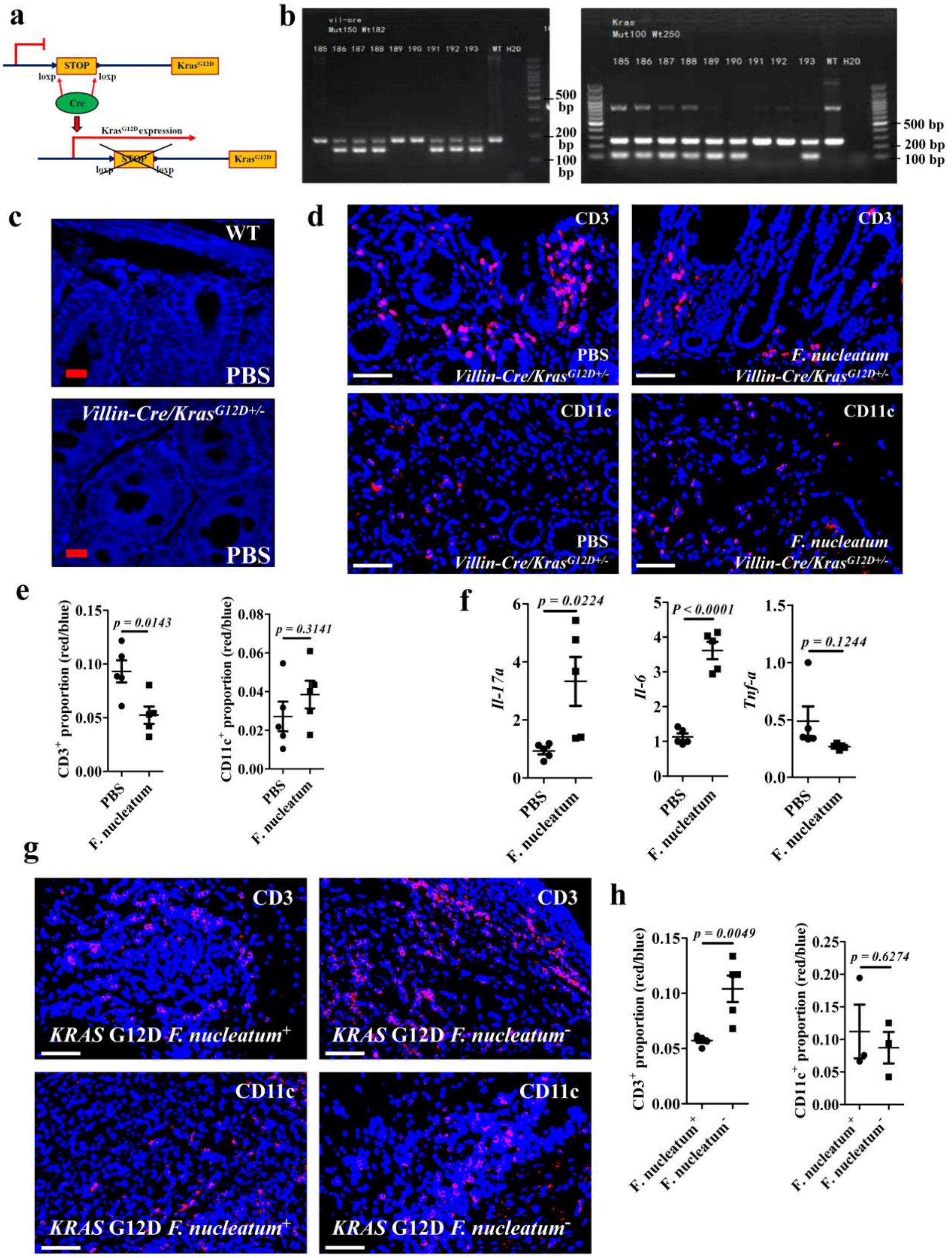

Supplementary Fig. 1 a Schematic diagram of *Villin-Cre/Kras*$^{G12D+/-}$ mouse model construction. **b** *Villin-Cre/Kras*$^{G12D+/-}$ genotyping using 150 bp band for *Cre* and 100 bp band for *Kras*^{G12D}. **c** FISH detection of *F. nucleatum* in colonic tissues derived from PBS treated-*Villin-Cre/Kras*$^{G12D+/-}$ mice and WT littermates using a

Cy3-conjugated *F.* nucleatum specific probe (red), n = 5, scale bar: 50 μm. **d** Representative immunofluorescence detection of CD3 and CD11c positive cells in colonic tissues of PBS and *F. nucleatum* treated *Villin-Cre/Kras*$^{G12D+/-}$ mice, scale bar: 50 μm. **e** Statistical analysis of the results in (**d**). Significant differences are indicated: two-tailed Student's t-test, n = 5 per group (mean ± SEM). **f** qPCR analysis of *Il-17a*, *Il-6*, *Tnf-α* mRNA expression in colonic tissues of PBS and *F. nucleatum* treated *Villin-Cre/Kras*$^{G12D+/-}$ mice. Significant differences are indicated: two-tailed Student's t-test, n = 5 per group (mean ± SEM). **g** Representative immunofluorescence detection of CD3 and CD11c positive cells in in *KRAS* p.G12D mutant patients with low *F. nucleatum* abundance (*F. nucleatum*$^{-}$) and high *F. nucleatum* abundance (*F. nucleatum*$^{+}$), scale bar: 50 μm. **h** Statistical analysis of the results in (**g**). Significant differences are indicated: two-tailed Student's t-test, n = 5 (left panel) and 3 (right panel) per group (mean ± SEM). Source data are provided as a Source Data file.

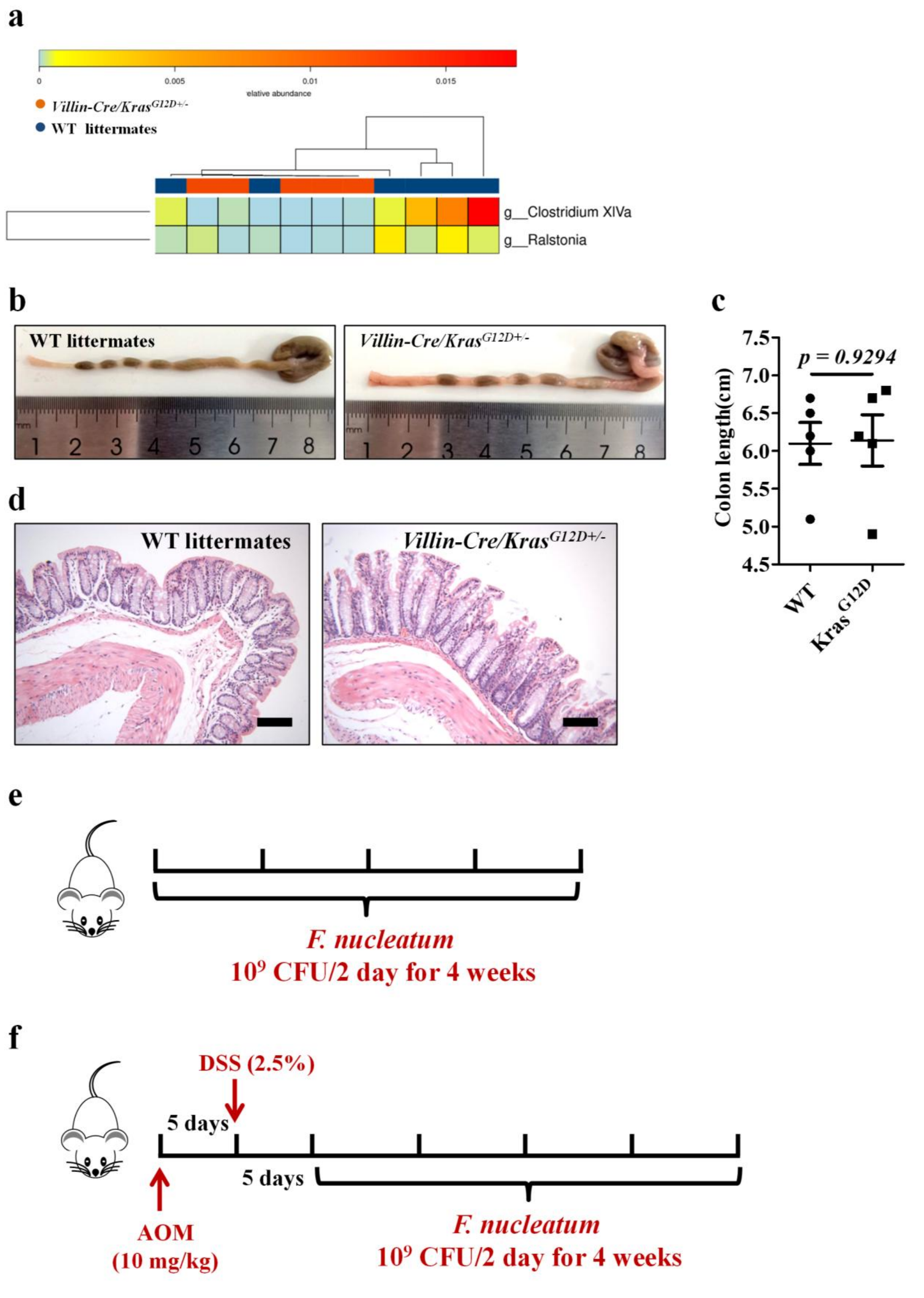

Supplementary Fig. 2 a Heat map of top differentially abundant genera between *Villin-Cre/Kras*$^{G12D+/-}$ mice (n = 5) and WT littermates (n = 6). **b** Representative images of the colons of *Villin-Cre/Kras*$^{G12D+/-}$ mice and WT littermates. **c** Statistical analysis of the colon length of *Villin-Cre/Kras*$^{G12D+/-}$ mice and WT littermates.

Significant differences are indicated: two-tailed Student's t-test, n = 5 per group (mean ± SEM). **d** Representative H&E staining of the colons of *Villin-Cre/Kras*$^{G12D+/-}$ mice and WT littermates, scale bar: 50 μm. **e** Schematic diagram of the experimental design and timeline of mouse models of Fig. 3a. **f** Schematic diagram of the experimental design and timeline of mouse models of Fig. 3b. Source data are provided as a Source Data file.

a

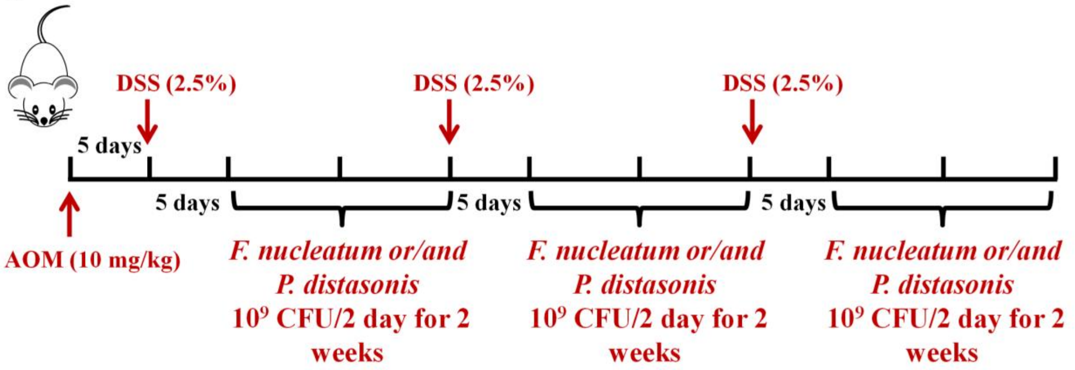

Supplementary Fig. 3 a Schematic diagram of the experimental design and timeline of mouse models of Fig. 4a.

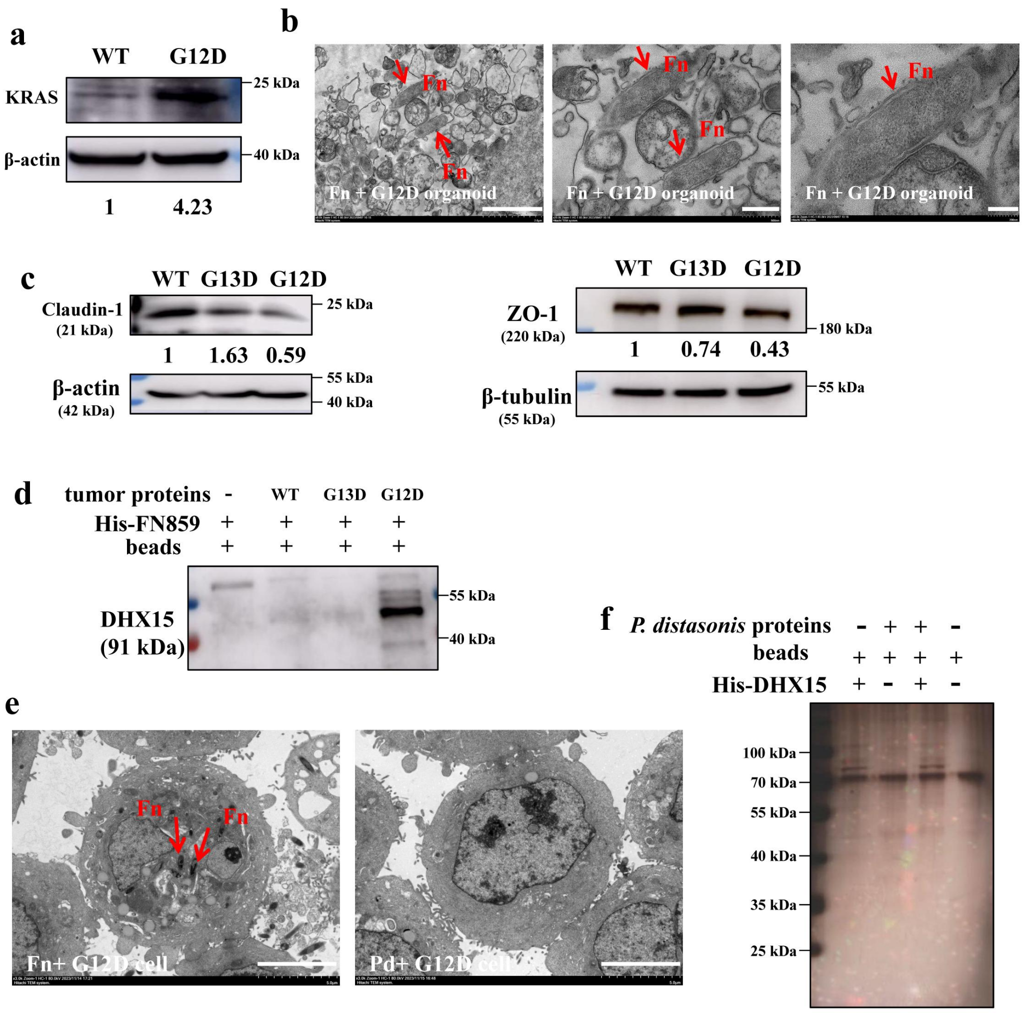

Supplementary Fig. 4 **a** HT-29 cells were transfected by *KRAS* G12D-sgRNA-Cas-EGFP and negative scramble control-EGFP lentiviral plasmids. Western blot analysis of KRAS expression in *KRAS* WT cells and *KRAS* p.G12D cells. **b** *F. nucleatum* was visible inside the *KRAS* p.G12D organoids by transmission electron microscope, scale bar: 20μm, 5μm, 2 μm respectively. **c** Western blot analysis of claudin-1 and ZO-1 expression in *KRAS* WT, *KRAS* p.G12D and *KRAS* p.G12D tumor cells. **d** Pull-down assays were performed and validation of the FN1859-DHX15 interaction in *KRAS* WT, *KRAS* p.G12D and *KRAS* p.G12D CRC patient samples by western blot. **e** TEM images of G12D cells incubated with *F. nucleatum* (left panel) and *P. distasonis* (right panel), scale bar: 50μm. **f** Pull-down assays were performed and validation of the *P. distasonis*-DHX15 interaction by silver staining. Data are representative of two independent experiments. Source data are provided as a Source Data file.

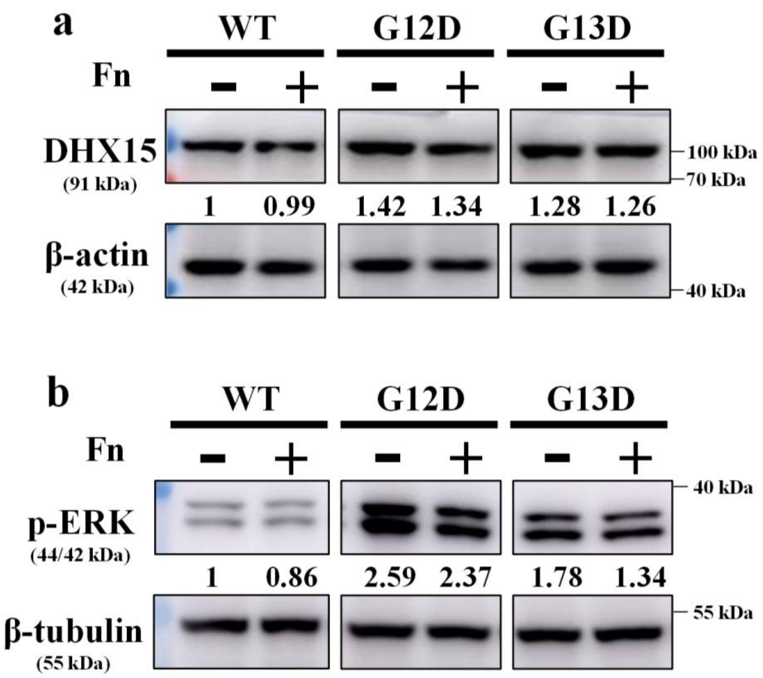

Supplementary Fig. 5 a Western blotting analysis of DHX15 expression in *KRAS* WT, *KRAS* p.G12D and *KRAS* p.G13D tumor cells after PBS or *F. nucleatum* treatment. **b** Western blotting analysis of p-ERK expression in *KRAS* WT, *KRAS* p.G12D and *KRAS* p.G13D tumor cells after PBS or *F. nucleatum* treatment. Data are representative of three independent experiments. Source data are provided as a Source Data file.

a

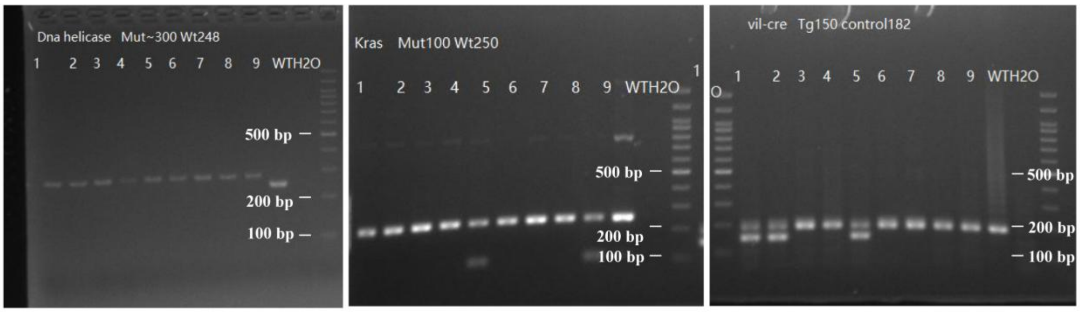

Supplementary Fig. 6 a. *Villin-Cre/Kras*$^{G12D+/-}$*/Dhx15*$^{fl/fl}$ genotyping using 150 bp band for *Cre*, 100 bp band for *Kras*$^{\mathrm{G12D}}$ and 300 bp band for *Dhx15*.

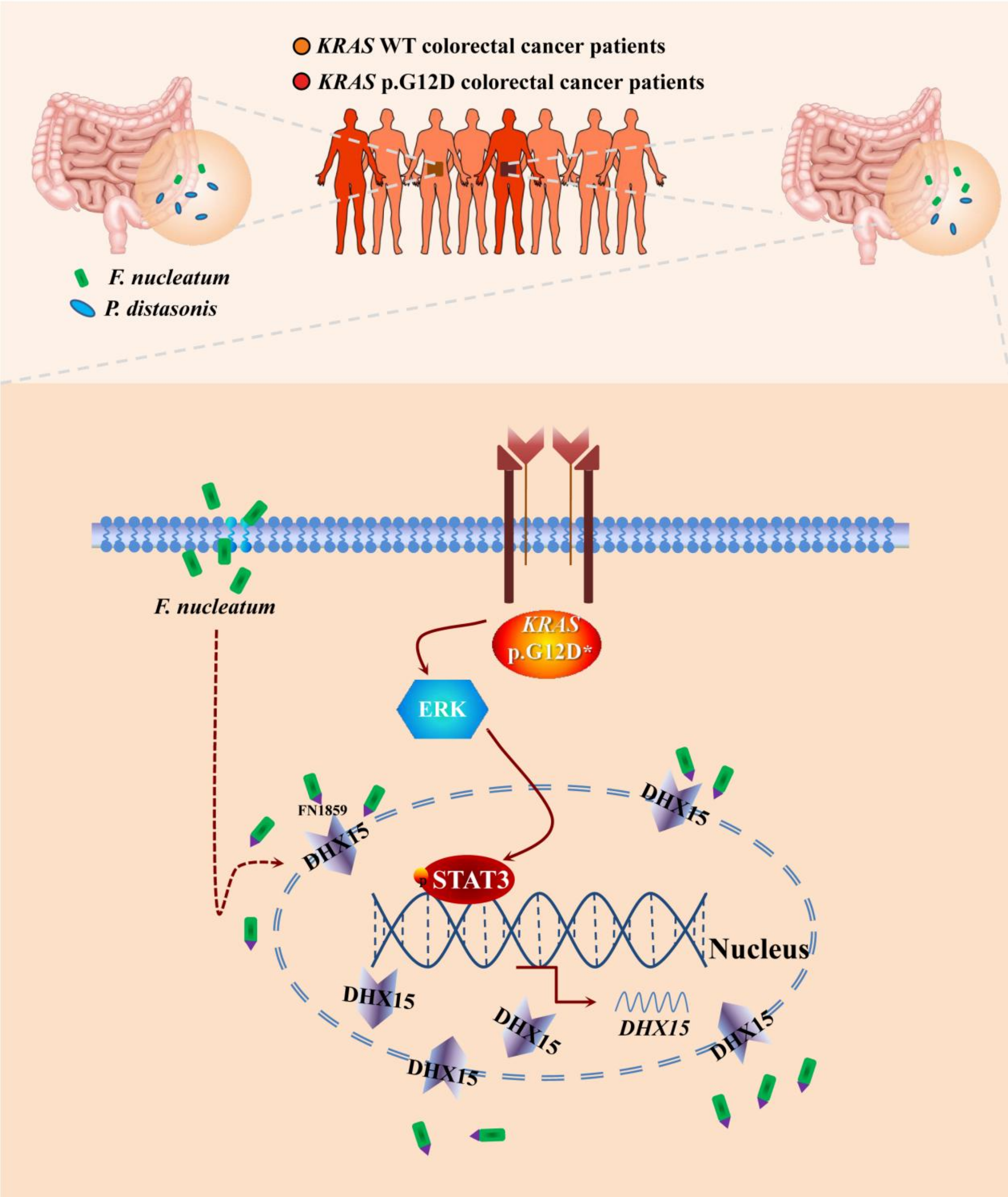

Supplementary Fig. 7 *KRAS* p.G12D mutant colorectal cancer cells induce more *F. nucleatum* enrichment and the invasion of *F. nucleatum* could be antagonized by *P. distasonis*. The mutation leads to ERK-STAT3 signaling pathway activation and DHX15 overexpression. *F. nucleatum* invaded into *KRAS* p.G12D mutant colorectal cancer cells and bind to DHX15 to potentiates colorectal tumorigenesis. The oncogenic effect of *F. nucleatum* depends on somatic genetics and gut microbial

ecology and personalized modulation of the gut microbiota may provide a more targeted strategy for CRC treatment.

Supplementary Tables

Supplementary Table 1. Information of CRC patients.

Patients' characteristics of CRC in Fig. 1b

Gender	Age	Tumor location	Tumor size	Tumor invasion	Lymph node metastasis	distant metastasis
**Female**	**≤65 years**	**Rectal**	**≤5cm**	**T_1-T_2**	**N0**	**Absent**
75	65	100	97	77	15	154
**Male**	**＞65years**	**Colon**	**＞5cm**	**T_3-T_4**	**N1**	**Present**
164	65	138	142	162	43	85
					**N2**	
					181	

Patients' characteristics of human colorectal tumor organoids

Gender	Age	Tumor location	Tumor size	Tumor invasion	Lymph node metastasis	distant metastasis
**Female**	**≤65 years**	**Rectal**	**≤5cm**	**T_1-T_2**	**N0**	**Absent**
4	6	11	8	5	7	9
**Male**	**＞65years**	**Colon**	**＞5cm**	**T_3-T_4**	**N1**	**Present**
11	9	4	7	10	6	6
					**N2**	
					2	

Supplementary Table 2. The data of logistic regression model (two-sided) that *P. distasonis* as an outcome with *KRAS*, *F. nucleatum*, and their interaction term (*KRAS* x *F. nucleatum*) as exposures.

			Confidence limit (CI)	
	p value	OR	Lower limits	Upper limits
F. nucleatum	0.028	0.548	0.321	0.936
KRAS	0.542	0.849	0.502	1.437

Supplementary Table 3. The differential proteins pulled by FN1859 in *KRAS* WT, *KRAS* p.G12D mutation and *KRAS* p.G13D mutation HT-29 cells.

Accession	Gene	Mw(kD)	G12D	G13D	NC	log2(G13D/G12D)	log2(MeanSP)	Diff Sig	log2(NC/G12D)	log2(MeanSP)	Diff Sig
P02545	LMNA	74.139	1	1	30	0	0		4.9069	3.9542	++
P23246	SFPQ	76.149	1	1	8	0	0		3	2.1699	++
O43143	DHX15	90.933	5	1	1	-2.3219	1.585	--	-2.3219	1.585	--
Q15233	NONO	54.232	1	1	5	0	0		2.3219	1.585	++

Supplementary Table 4. Primers for PCR.

Primers for PCR			
**Genes**	**Species**	**Forward primer**	**Reverse primer**
DHX15	Human	GGTCCACAGATCTTTTCTTGTGC	CCTAGCCATAAGGGCTGTACC
Il-17A	Mouse	TTTAACTCCCTTGGCGCAAAA	CTTTCCCTCCGCATTGACAC
Il-6	Mouse	TAGTCCTTCCTACCCCAATTTCC	TTGGTCCTTAGCCACTCCTTC
Tnf-α	Mouse	GACGTGGAACTGGCAGAAGAG	TTGGTGGTTTGTGAGTGTGAG

Supplementary Table 5. Antibodies for WB.

Antibodies for WB			
Mouse anti-β-actin antibody	1:2000	Cell Signaling Technology	#3700S
Mouse anti-β-tubulin antibody	1:1000	SantaCruz	#SC-5274
Rabbit anti-DHX15 Ab	1:1000	Proteintech	#12265-1-AP
Rabbit anti-p-ERK1/2 Ab	1:2000	Cell Signaling Technology	#4370S
Rabbit anti-p-AKT Ab	1:1000	Cell Signaling Technology	#4060S
Rabbit anti-KRAS Ab	1:1000	Abcam	#ab180772
Rabbit anti-Claudin-1 Ab	1:1000	Proteintech	#13050-1-AP
Rabbit anti-ZO-1 Ab	1:1000	Proteintech	#21773-1-AP
HRP-labeled goat anti-mouse IgG(H+L)	1:2000	Beyotime	#A0216
HRP-labeled goat anti-rabbit IgG(H+L)	1:1000	Beyotime	#A0208

Supplementary Table 6. 16S sequencing data of *P. disatasonis*.

Description	Max Score	Total Score	Query Cover	E value	Per. Ident	Accession
Uncultured organism clone ELU0049-T299-S-NIPCRAMgANa_000121 small subunit ribosomal RNA gene, partial sequence	2663	2663	99%	0.0	99.86%	HQ761955.1
Parabacteroides sp. strain CT06 16S ribosomal RNA gene, partial sequence	2662	2662	99%	0.0	99.86%	KY703631.1
Uncultured bacterium clone SJTU_D_07_93 16S ribosomal RNA gene, partial sequence	2662	2662	100%	0.0	99.79%	EF401259.1
Parabacteroides distasonis ATCC 8503 isolate Parabacteroides distasonis 82G9 genome assembly, chromosome: 1	2656	18596	100%	0.0	99.72%	LR215978.1
Parabacteroides sp. CT06, complete genome	2656	18285	100%	0.0	99.72%	CP022754.1
Uncultured bacterium clone SJTU_D_03_79 16S ribosomal RNA gene, partial sequence	2656	2656	100%	0.0	99.72%	EF400947.1
Uncultured organism clone ELU0049-T299-S-NIPCRAMgANa_000452 small subunit ribosomal RNA gene, partial sequence	2652	2652	99%	0.0	99.72%	HQ762286.1
Uncultured organism clone ELU0133-T352-S-NI_000178 small subunit ribosomal RNA gene, partial sequence	2652	2652	99%	0.0	99.72%	HQ794006.1
Uncultured organism clone ELU0073-T501-S-NIPCRAMgANa_000631 small subunit ribosomal RNA gene, partial sequence	2652	2652	99%	0.0	99.79%	HQ774231.1
Uncultured organism clone ELU0008-T58-S-NI_000191 small subunit ribosomal RNA gene, partial sequence	2652	2652	99%	0.0	99.72%	HQ740098.1

S000152 1 1450bp

AGGATGAACGCTAGCGACAGGCTTAACACATGCAAGTCGAGGGGCAGCACAGGTAGCAATACCGGGT
GGCGACCGGCGCACGGGTGAGTAACGCGTATGCAACTTGCCTATCAGAGGGGGATAACCCGGCGAAA
GTCGGACTAATACCGCATGAAGCAGGGATCCCGCATGGGAATATTTGCTAAAGATTCATCGCTGATAGA
TAGGCATGCGTTCCATTAGGCAGTTGGCGGGGTAACGGCCCACCAAACCGACGATGGATAGGGGTTCT
GAGAGGAAGGTCCCCCACATTGGTACTGAGACACGGACCAAACTCCTACGGGAGGCAGCAGTGAGG
AATATTGGTCAATGGCCGAGAGGCTGAACCAGCCAAGTCGCGTGAGGGATGAAGGTTCTATGGATCGT
AAACCTCTTTTATAAGGGAATAAAGTGCGGGACGTGTCCCGTTTTGTATGTACCTTATGAATAAGGATC
GGCTAACTCCGTGCCAGCAGCCGCGGTAATACGGAGGATCCGAGCGTTATCCGGATTTATTGGGTTTAA
AGGGTGCGTAGGCGGCCTTTTAAGTCAGCGGTGAAAGTCTGTGGCTCAACCATAGAATTGCCGTTGAA
ACTGGGGGGCTTGAGTATGTTTGAGGCAGGCGGAATGCGTGGTGTAGCGGTGAAATGCATAGATATCA
CGCAGAACCCCGATTGCGAAGGCAGCCTGCCAAGCCATTACTGACGCTGATGCACGAAAGCGTGGGG
ATCAAACAGGATTAGATACCCTGGTAGTCCACGCAGTAAACGATGATCACTAGCTGTTTGCGATACATT
GTAAGCGGCACAGCGAAAGCGTTAAGTGATCCACCTGGGGAGTACGCCGGCAACGGTGAAACTCAA
AGGAATTGACGGGGGCCCGCACAAGCGGAGGAACATGTGGTTTAATTCGATGATACGCGAGGAACCT
TACCCGGGTTTGAACGCATTCGGACCGAGGTGGAAACACCTTTTCTAGCAATAGCCGTTTGCGAGGTG
CTGCATGGTTGTCGTCAGCTCGTGCCGTGAGGTGTCGGCTTAAGTGCCATAACGAGCGCAACCCTTGC

CACTAGTTACTAACAGGTAAAGCTGAGGACTCTGGTGGGACTGCCAGCGTAAGCTGCGAGGAAGGCG
GGGATGACGTCAAATCAGCACGGCCCTTACATCCGGGGCGACACACGTGTTACAATGGCGTGGACAA
AGGGAAGCCACCTGGCGACAGGGAGCGAATCCCCAAACCACGTCTCAGTTCGGATCGGAGTCTGCAA
CCCGACTCCGTGAAGCTGGATTCGCTAGTAATCGCGCATCAGCCATGGCGCGGTGAATACGTTCCCGG
GCCTTGTACACACCGCCCGTCAAGCCATGGGAGCCGGGGGTACCTGAAGTCCGTAACCGCGAGGATC
GGCCTAGGGTAAAACTGGTGACTGGGGCTA

The strain is identified as *Parabacteroides distasonis*.

Supplementary Table 7. Identification of cultured *F. nucleatum* and *P. distosonis* by Nanopore Sequencing.

SPECIES	SEQUENCE NUMBER	PROPORTION	NCBI ID	FAMILY	GENUS
Fusobacterium nucleatum	479265	97.58	851	*Bacteria*	*Fusobacterium*
Parabacteroides distasonis	2391	0.49	823	*Bacteria*	*Parabacteroides*
Escherichia coli	2224	0.45	562	*Bacteria*	*Escherichia*
unclassified	756	0.15	0	*unclassified*	*unclassified*
Fusobacterium sp. oral taxon 203	489	0.1	671211	*Bacteria*	*Fusobacterium*
Fusobacterium hwasookii	488	0.1	1583098	*Bacteria*	*Fusobacterium*
Fusobacterium pseudoperiodonticum	743	0.1	2663009	*Bacteria*	*Fusobacterium*
Parabacteroides sp. CT06	111	0.02	2025876	*Bacteria*	*Parabacteroides*
Luteimonas granuli	32	0.01	1176533	*Bacteria*	*Luteimonas*
Bacteroides fragilis	31	0.01	817	*Bacteria*	*Bacteroides*
**SPECIES**	**SEQUENCE NUMBER**	**PROPORTION**	**NCBI ID**	**FAMILY**	**GENUS**
Parabacteroides distasonis	99861	76.01	823	*Bacteria*	*Parabacteroides*
Parabacteroides sp. CT06	5210	3.97	2025876	*Bacteria*	*Parabacteroides*
unclassified	4319	3.29	0	*unclassified*	*unclassified*
Fusobacterium nucleatum	2266	1.72	851	*Bacteria*	*Fusobacterium*
Escherichia coli	1301	0.99	562	*Bacteria*	*Escherichia*
Bacteroides fragilis	1122	0.85	817	*Bacteria*	*Bacteroides*
Bacteroides uniformis	711	0.54	820	*Bacteria*	*Bacteroides*
Paraprevotella xylaniphila	495	0.38	454155	*Bacteria*	*Paraprevotella*
Bacteroides sp. HF- 5287	468	0.36	2650157	*Bacteria*	*Bacteroides*
Bacteroides xylanisolvens	276	0.21	371601	*Bacteria*	*Bacteroides*
