# Supplementary material for: Fusobacterium nucleatum promotes tumor progression in KRAS p.G12D-mutant colorectal cancer by binding to DHX15
Source: Nat Commun. 2024 Feb 24;15:1688. doi: 10.1038/s41467-024-45572-w (PMC10894276; doi:10.1038/s41467-024-45572-w)
Supplement: Supplementary file 3 — Reporting Summary [file 41467_2024_45572_MOESM3_ESM.pdf]

nature portfolio

Corresponding author(s): Huiyuan Zhu, Qing Wei and Huanlong Qin

Last updated by author(s): Jan. 26, 2024

Reporting Summary

Nature Portfolio wishes to improve the reproducibility of the work that we publish. This form provides structure for consistency and transparency in reporting. For further information on Nature Portfolio policies, see our Editorial Policies and the Editorial Policy Checklist.

Statistics

For all statistical analyses, confirm that the following items are present in the figure legend, table legend, main text, or Methods section.

n/a	Confirmed	
☐	☒	The exact sample size (*n*) for each experimental group/condition, given as a discrete number and unit of measurement
☐	☒	A statement on whether measurements were taken from distinct samples or whether the same sample was measured repeatedly
☐	☒	The statistical test(s) used AND whether they are one- or two-sided<br>*Only common tests should be described solely by name; describe more complex techniques in the Methods section.*
☒	☐	A description of all covariates tested
☐	☒	A description of any assumptions or corrections, such as tests of normality and adjustment for multiple comparisons
☐	☒	A full description of the statistical parameters including central tendency (e.g. means) or other basic estimates (e.g. regression coefficient) AND variation (e.g. standard deviation) or associated estimates of uncertainty (e.g. confidence intervals)
☐	☒	For null hypothesis testing, the test statistic (e.g. *F*, *t*, *r*) with confidence intervals, effect sizes, degrees of freedom and *P* value noted<br>*Give P values as exact values whenever suitable.*
☒	☐	For Bayesian analysis, information on the choice of priors and Markov chain Monte Carlo settings
☒	☐	For hierarchical and complex designs, identification of the appropriate level for tests and full reporting of outcomes
☒	☐	Estimates of effect sizes (e.g. Cohen's *d*, Pearson's *r*), indicating how they were calculated

Our web collection on statistics for biologists contains articles on many of the points above.

Software and code

Policy information about availability of computer code

Data collection	For 16S rRNA sequencing, the constructed libraries were sequenced with an Illumina MiSeq platform. For targeted gene sequencing, the constructed libraries were sequenced with the Illumina NovaSeq 6000. The real-time quantitative PCR data was collected in a Vii7 Real-Time PCR system. Western blotting signals were imaged with GE Amersham Imager 600. Histological and immunohistochemistry images were captured by an Olympus BX51 microscope. Immunofluorescent images were captured by a Zeiss LSM900 confocal microscopy system. Electron microscope images were captured by the Thermo Fisher Scientific Titan Krios G3 300kV cryogenic transmission electron microscopy and Aquilos2 double-beam scanning electron microscopy.
Data analysis	The data were analyzed using GraphPad Prism 5, SPSS 22.0 and Image J.

For manuscripts utilizing custom algorithms or software that are central to the research but not yet described in published literature, software must be made available to editors and reviewers. We strongly encourage code deposition in a community repository (e.g. GitHub). See the Nature Portfolio guidelines for submitting code & software for further information.

Data

Policy information about availability of data

All manuscripts must include a data availability statement. This statement should provide the following information, where applicable:

- Accession codes, unique identifiers, or web links for publicly available datasets
- A description of any restrictions on data availability
- For clinical datasets or third party data, please ensure that the statement adheres to our policy

The sequencing data in this study have been deposited in the GSA with accession number CRA013275 (https://ngdc.cncb.ac.cn/gsa/browse/CAR013275), CRA013274 (https://ngdc.cncb.ac.cn/gsa/browse/CAR013274), CRA013276 (https://ngdc.cncb.ac.cn/gsa/browse/CRA013276), CRA013455 (https://ngdc.cncb.ac.cn/gsa/browse/CRA013455), HRA006025 (https://ngdc.cncb.ac.cn/gsa-human/browse/HRA006025). Dataset HRA006025 is available under restricted access due to it is human genetics dataset; access can be obtained by the DAC (Data Access Committees) of the GSA-human database. The approximate response time for accession requests is about 3 days. Once access has been approved, the data will be available to 3 months. The user can also contact the corresponding author directly. The mass spectrometry proteomics data have been deposited to the ProteomeXchange Consortium via the PRIDE partner repository with the dataset identifier PXD048684 (https://www.ebi.ac.uk/pride/archive/projects/PXD048684), PXD048686 (https://www.ebi.ac.uk/pride/archive/projects/PXD048686). The other data supporting the findings of this study are available within the paper or supplementary materials. Source data are provided with this papper.

Research involving human participants, their data, or biological material

Policy information about studies with human participants or human data. See also policy information about sex, gender (identity/presentation), and sexual orientation and race, ethnicity and racism.

Reporting on sex and gender	Sex was not considered in study designed.
Reporting on race, ethnicity, or other socially relevant groupings	Race and ethnicity were not considered in study designed.
Population characteristics	The baseline characteristics of colorectal cancer patient are provided in the supplementary Table 1.
Recruitment	All colorectal cancer tumor tissues (n=254) and adjacent normal tissues (n=24) were recruited from patients with colorectal adenocarcinoma who underwent a surgical resection. All individuals provided informed consent.
Ethics oversight	The study was performed in accordance with the principles of the Declaration of Helsinki and approved by the Ethics Committee of Shanghai Tenth People's Hospital (SHDSYY-2019-2751)

Note that full information on the approval of the study protocol must also be provided in the manuscript.

Field-specific reporting

Please select the one below that is the best fit for your research. If you are not sure, read the appropriate sections before making your selection.

☒ Life sciences ☐ Behavioural & social sciences ☐ Ecological, evolutionary & environmental sciences

For a reference copy of the document with all sections, see nature.com/documents/nr-reporting-summary-flat.pdf

Life sciences study design

All studies must disclose on these points even when the disclosure is negative.

Sample size	In vitro and in vivo experiments were done at least two times, either in duplicate or triplicate. In general, at least three mice were used per group per experiment. Sample size was largedly based on Villin-Cre/Kras G12D+/- mice and age matched controls.
Data exclusions	No data were excluded from analyses.
Replication	All experiments were performed with independent replicates as described in the figure legends.
Randomization	Mice with comparable age, weight and indicated genotypes were randomly selected from housing cages and then assigned in groups with no bias for further treatment. Specimens for immunohistochemical staining and immunofluorescence were randomized chosen and assayed together.
Blinding	Investigators were blinded to group allocation during data collection and analysis.

Behavioural & social sciences study design

All studies must disclose on these points even when the disclosure is negative.

Study description

Research sample

Sampling strategy

Data collection

Timing

Data exclusions

Non-participation

Randomization

Ecological, evolutionary & environmental sciences study design

All studies must disclose on these points even when the disclosure is negative.

Study description

Research sample

Sampling strategy

Data collection

Timing and spatial scale

Data exclusions

Reproducibility

Randomization

Blinding

Did the study involve field work? ☐ Yes ☐ No

Field work, collection and transport

Field conditions

Location

Access & import/export

Disturbance

Reporting for specific materials, systems and methods

We require information from authors about some types of materials, experimental systems and methods used in many studies. Here, indicate whether each material, system or method listed is relevant to your study. If you are not sure if a list item applies to your research, read the appropriate section before selecting a response.

Materials & experimental systems

n/a	Involved in the study
☐	☒ Antibodies
☐	☒ Eukaryotic cell lines
☒	☐ Palaeontology and archaeology
☐	☒ Animals and other organisms
☒	☐ Clinical data
☒	☐ Dual use research of concern
☒	☐ Plants

Methods

n/a	Involved in the study
☒	☐ ChIP-seq
☒	☐ Flow cytometry
☒	☐ MRI-based neuroimaging

Antibodies

Antibodies used

Anti-β-actin antibody (Cell Signaling Technology, cat: 3700S, WB-1:2000)
Anti-β-tubulin antibody (SantaCruz, cat: SC-5274, WB-1:1000)
Anti-DHX15 antibody (Proteintech, cat: 12265-1-AP, WB-1:1000)
Anti-p-ERK1/2 antibody (Cell Signaling Technology, cat: 4370S, WB-1:2000)
Anti-p-AKT antibody (Cell Signaling Technology, cat: 4060S, WB-1:1000)
Anti-CD11c antibody (Abcam, cat: ab254183, IF-1:100)
Anti-CD3 antibody (Abcam, cat: ab11089, IF-1:100)
Anti-KRAS antibody (Abcam, cat: ab180772, WB-1:1000)
Anti-Claudin-1 antibody (Proteintech, cat: 13050-1-AP, WB-1:1000)
Anti-ZO-1 antibody (Proteintech, cat: 21773-1-AP, WB-1:1000)
HRP-labeled goat anti-mouse IgG(H+L) (Beyotime, cat: A0216, WB-1:2000)
HRP-labeled goat anti-rabbit IgG(H+L) (Beyotime, cat: A0208, WB-1:1000)

Validation

Anti-β-actin antibody (Cell Signaling Technology, cat: 3700s)
https://www.cellsignal.cn/products/primary-antibodies/b-actin-8h10d10-mouse-mab/3700?site-search-type=Products&N=4294956287&Ntt=3700s&fromPage=plp& requestid=1747042
Anti-β-tublin antibody (SantaCruz, cat: SC-5274)
http://www.scbt.com/p/beta-tubulin-antibody-d-10?requestFrom=search
Anti-DHX15 antibody (Proteintech, cat: 12265-1-AP)
http://ptgcn.com/products/DHX15-Antibody-12265-1-AP.htm
Anti-p-ERK1/2 antibody (Cell Signaling Technology, cat:4370S)
http://www.cellsignal.cn/products/primary-antibodies/phospho-p44-42-mapk-erk1-2-thr202-tyr204-d13-14-4e-xp-rabbit-mab/4370?site-search-type=Products&N=4294956287&Ntt=4370s&fromPage=plp& requestid=1748054
Anti-p-AKT antibody (Cell Signaling Technology, cat: 4060S)
http://www.cellsignal.cn/products/primary-antibodies/phospho-akt-ser473-d9e-xp-rabbit-mab/4060?site-search-type=Products&N=4294956287&Ntt=4060s&fromPage=plp& requestid=1748682
Anti-CD11c antibody (Abcam, cat: ab254183)
https://www.abcam.cn/products/primary-antibodies/cd11c-antibody-kb90-ab254183.html
Anti-CD3 antibody (Abcam, cat: ab11089)
https://www.abcam.cn/products/primary-antibodies/cd3-antibody-cd3-12-ab11089.html
Anti-KRAS antibody (Abcam, cat: ab180772)
https://www.abcam.cn/products/primary-antibodies/ras-antibody-ab180772.html
Anti-Claudin-1 antibody (Proteintech, cat: 13050-1-AP)
https://www.ptgcn.com/products/CLDN1-Antibody-13050-1-AP.htm
Anti-ZO-1 antibody (Proteintech, cat: 21773-1-AP)
https://www.ptgcn.com/products/ZO1-Antibody-21773-1-AP.htm
HRP-labeled goat anti-mouse IgG(H+L) (Beyotime, cat: A0216)
http://www.beyotime.com/product/A0216.htm
HRP-labeled goat anti-rabbit IgG(H+L) (Beyotime, cat: A0208)
http://www.beyotime.com/product/A0208.htm

Eukaryotic cell lines

Policy information about cell lines and Sex and Gender in Research

Cell line source(s)

Human HT-29 cell line (TCHu103, Cell Bank, Chinese Academy of Science) and human 293T (GNHu17, Cell Bank, Chinese Academy of Science) cell line was used.

Authentication

Authentication for human HT-29 was provided on website, https://www.cellbank.org.cn/search-detail.php?id=181; authentication for human 293T was provided on website, https://www.cellbank.org.cn/search-detail.php?id=24.
We authenticate each cell line by morphology.

Mycoplasma contamination

Cell lines tested negative for mycoplasma contamination.

Commonly misidentified lines (See ICLAC register)	No commonly misidentified cell lines were used in the study.

Palaeontology and Archaeology

Specimen provenance	
Specimen deposition	
Dating methods	

☐ Tick this box to confirm that the raw and calibrated dates are available in the paper or in Supplementary Information.

Ethics oversight	

Note that full information on the approval of the study protocol must also be provided in the manuscript.

Animals and other research organisms

Policy information about studies involving animals; ARRIVE guidelines recommended for reporting animal research, and Sex and Gender in Research

Laboratory animals	6-8 week old WT C57BL/6J, Villin-Cre/Kras G12D+/-, Villin-Cre/Kras G12D+/+ mice were purchased from Shanghai Model Organisms Center (Shanghai, China). Dhx15-floxed mice were provided by Prof. Shu Zhu (Division of Life Sciences and Medicine, University of Science and Technology of China, Hefei, China). All mice were housed in a specific pathogen-free environment in the Animal Laboratory Unit, Tongji University, China. Housing condition: 20 ± 2 ?, 50 ± 5% humidity, 12-12 light-dark cycles.
Wild animals	NO wild animals were used in this study.
Reporting on sex	Both female and male mice were used in this study.
Field-collected samples	No field-collected samples were used in this study.
Ethics oversight	All animal studies were approved by the Ethics Review Committee for Animal Experimentation at Shanghai Tenth People's Hospital.

Note that full information on the approval of the study protocol must also be provided in the manuscript.

Clinical data

Policy information about clinical studies

All manuscripts should comply with the ICMJE guidelines for publication of clinical research and a completed CONSORT checklist must be included with all submissions.

Clinical trial registration	
Study protocol	
Data collection	
Outcomes	

Dual use research of concern

Policy information about dual use research of concern

Hazards

Could the accidental, deliberate or reckless misuse of agents or technologies generated in the work, or the application of information presented in the manuscript, pose a threat to:

No | Yes

- [ ] [ ] Public health
- [ ] [ ] National security
- [ ] [ ] Crops and/or livestock
- [ ] [ ] Ecosystems
- [ ] [ ] Any other significant area

Experiments of concern

Does the work involve any of these experiments of concern:

No | Yes

- [ ] [ ] Demonstrate how to render a vaccine ineffective
- [ ] [ ] Confer resistance to therapeutically useful antibiotics or antiviral agents
- [ ] [ ] Enhance the virulence of a pathogen or render a nonpathogen virulent
- [ ] [ ] Increase transmissibility of a pathogen
- [ ] [ ] Alter the host range of a pathogen
- [ ] [ ] Enable evasion of diagnostic/detection modalities
- [ ] [ ] Enable the weaponization of a biological agent or toxin
- [ ] [ ] Any other potentially harmful combination of experiments and agents

Plants

Seed stocks

Novel plant genotypes

Authentication

ChIP-seq

Data deposition

- [ ] Confirm that both raw and final processed data have been deposited in a public database such as GEO.
- [ ] Confirm that you have deposited or provided access to graph files (e.g. BED files) for the called peaks.

Data access links
May remain private before publication.

Files in database submission

Genome browser session
(e.g. UCSC)

Methodology

Replicates

Sequencing depth

Antibodies

Peak calling parameters

Data quality

Software

Flow Cytometry

Plots

Confirm that:

- [ ] The axis labels state the marker and fluorochrome used (e.g. CD4-FITC).
- [ ] The axis scales are clearly visible. Include numbers along axes only for bottom left plot of group (a 'group' is an analysis of identical markers).
- [ ] All plots are contour plots with outliers or pseudocolor plots.
- [ ] A numerical value for number of cells or percentage (with statistics) is provided.

Methodology

Sample preparation

Instrument

Software

Cell population abundance

Gating strategy

- [ ] Tick this box to confirm that a figure exemplifying the gating strategy is provided in the Supplementary Information.

Magnetic resonance imaging

Experimental design

Design type

Design specifications

Behavioral performance measures

Acquisition

Imaging type(s)

Field strength

Sequence & imaging parameters

Area of acquisition

Diffusion MRI ☐ Used ☐ Not used

Preprocessing

Preprocessing software

Normalization

Normalization template

Noise and artifact removal

Volume censoring

Statistical modeling & inference

Model type and settings

Effect(s) tested

Specify type of analysis: ☐ Whole brain ☐ ROI-based ☐ Both

Statistic type for inference

(See Eklund et al. 2016)

Correction

Models & analysis

n/a	Involved in the study
☐	☐ Functional and/or effective connectivity
☐	☐ Graph analysis
☐	☐ Multivariate modeling or predictive analysis

Functional and/or effective connectivity

Graph analysis

Multivariate modeling and predictive analysis
