# Supplementary material for: Fusobacterium nucleatum promotes tumor progression in KRAS p.G12D-mutant colorectal cancer by binding to DHX15
Source: Nat Commun. 2024 Feb 24;15:1688. doi: 10.1038/s41467-024-45572-w (PMC10894276; doi:10.1038/s41467-024-45572-w)
Supplement: Supplementary file 4 — Source Data [file 41467_2024_45572_MOESM4_ESM.zip › Source Data Files/WB Source Data.pdf]

Figure 5f. Western blot analysis of *F. nucleatum* proteins incubated by biotinylated proteins from indicated cells and detected using avidin-conjugated horseradish peroxidase.

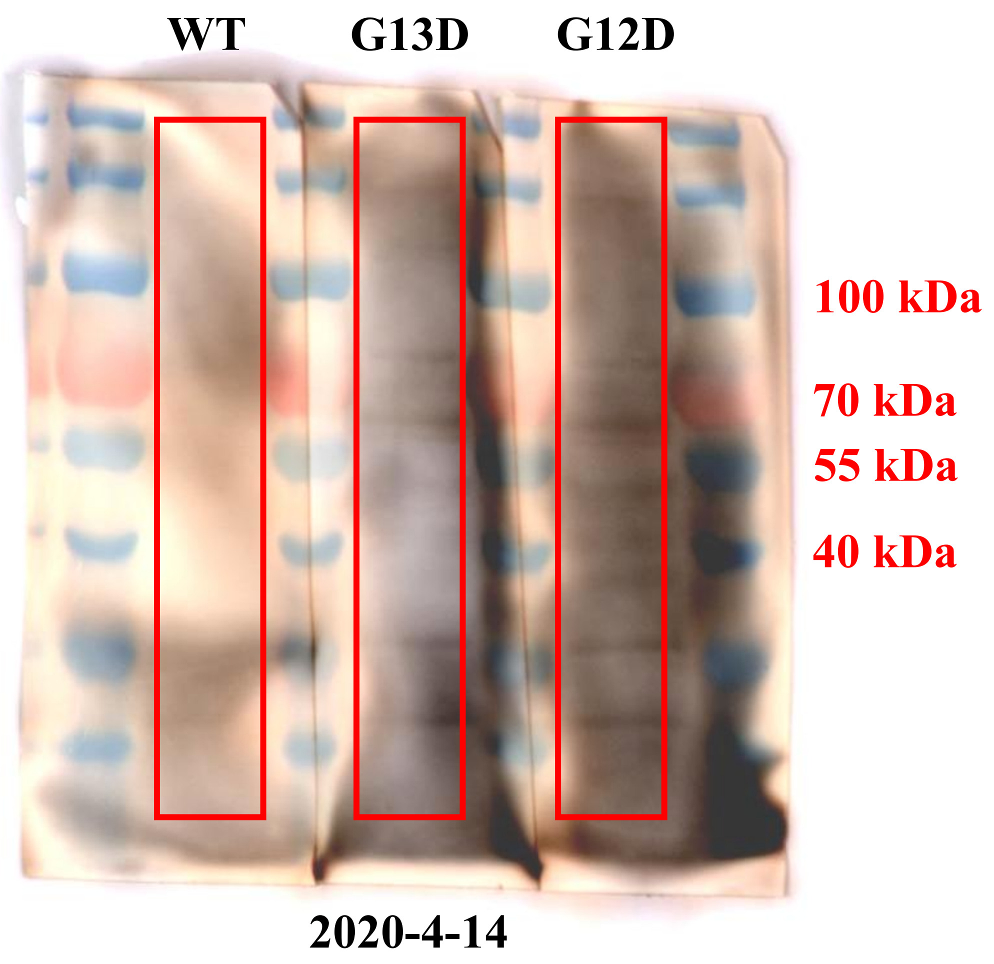

Figure 5g. Pull-down assays were performed and validation of the FN1859-DHX15 interaction in *KRAS* WT, *KRAS* p.G12D and *KRAS* p.G13D cells by western blot.

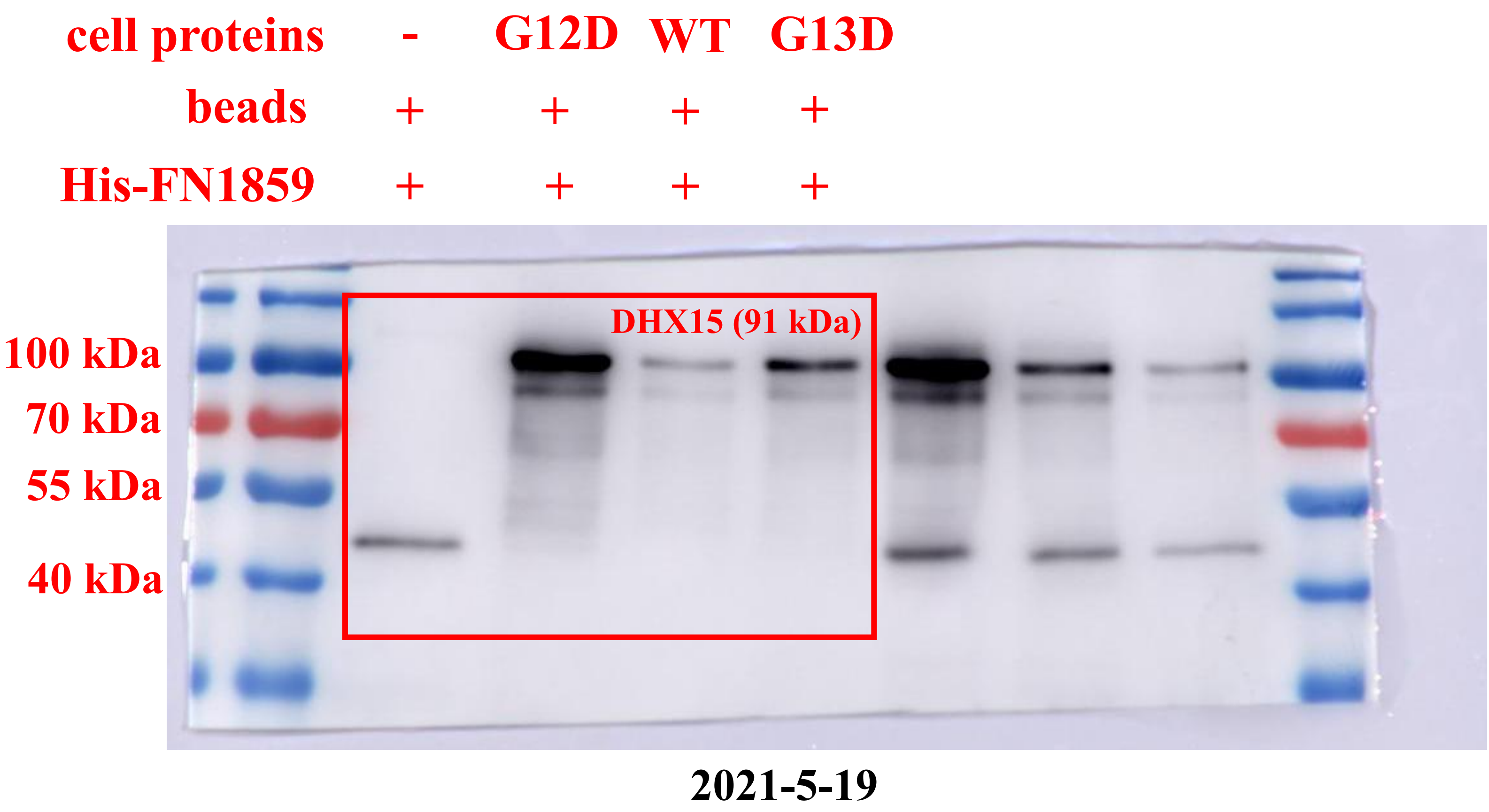

Figure 5i. Western blot analysis of DHX15 expression in *Villin-Cre/Kras*$^{G12D+/-}$ mice and WT littermates. Lanes representative of separate mice.

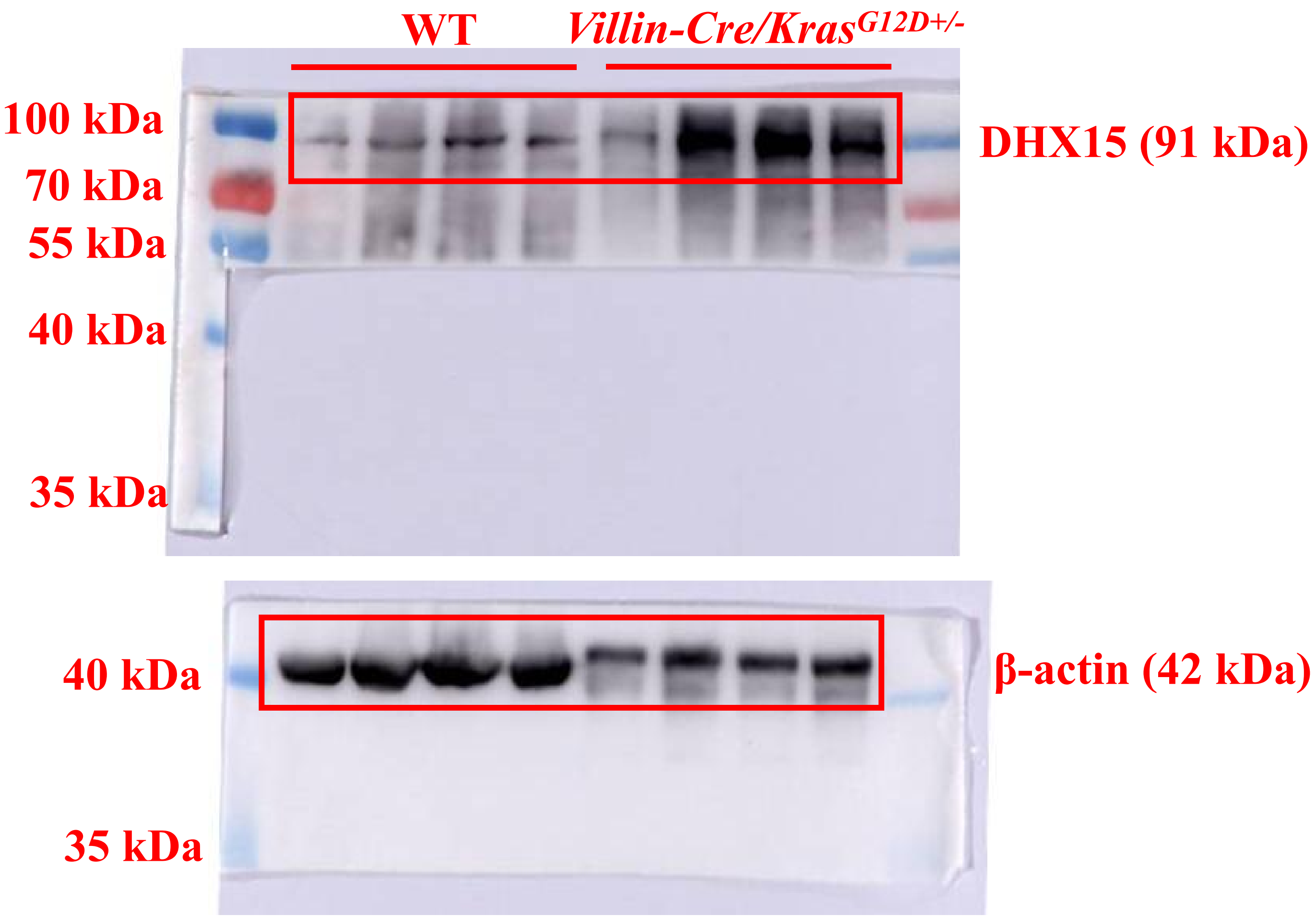

2020-12-29

Figure 5k. Western blot analysis of DHX15 expression in *KRAS* WT, *KRAS* p.G12D and *KRAS* p.G13D tumor cells.

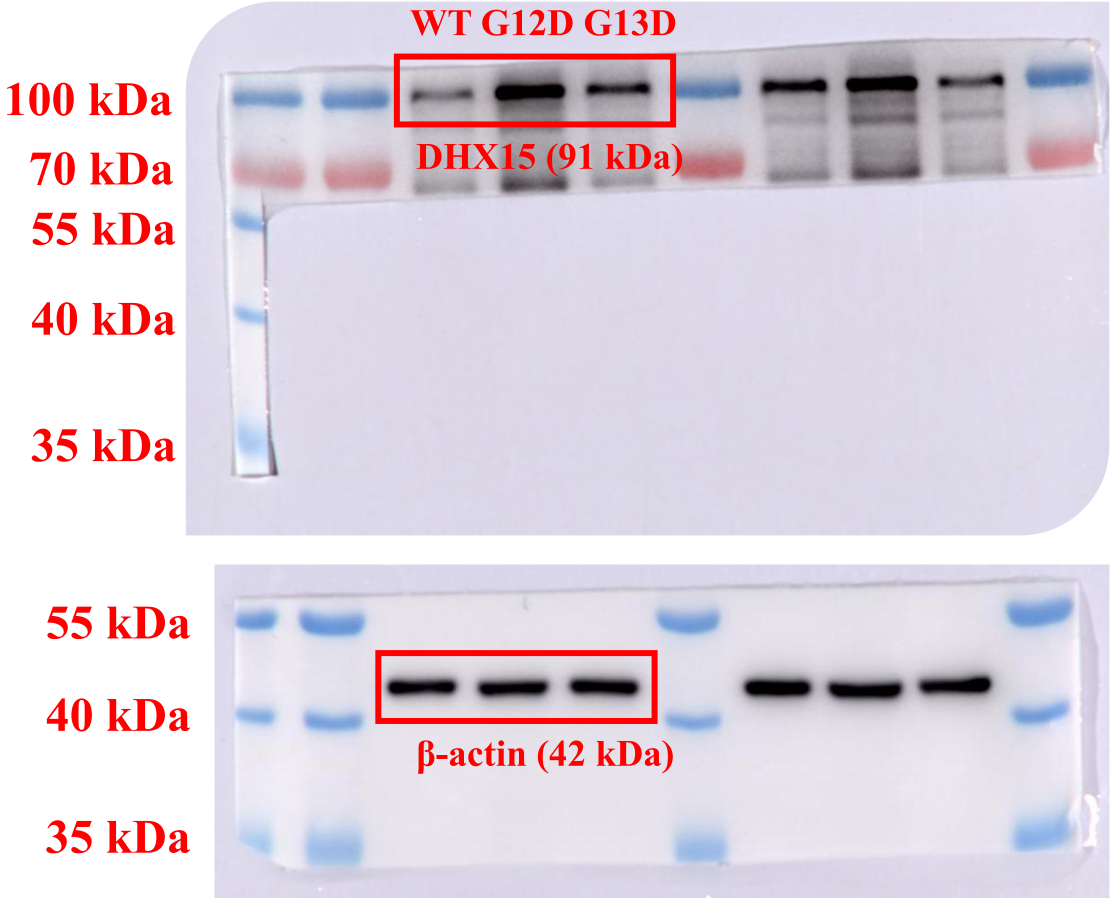

2020-12-03

Figure 6a. Western blot analysis of p-ERK, p-AKT and DHX15 expression in *KRAS* WT, *KRAS* p.G12D and *KRAS* p.G13D tumor cells.

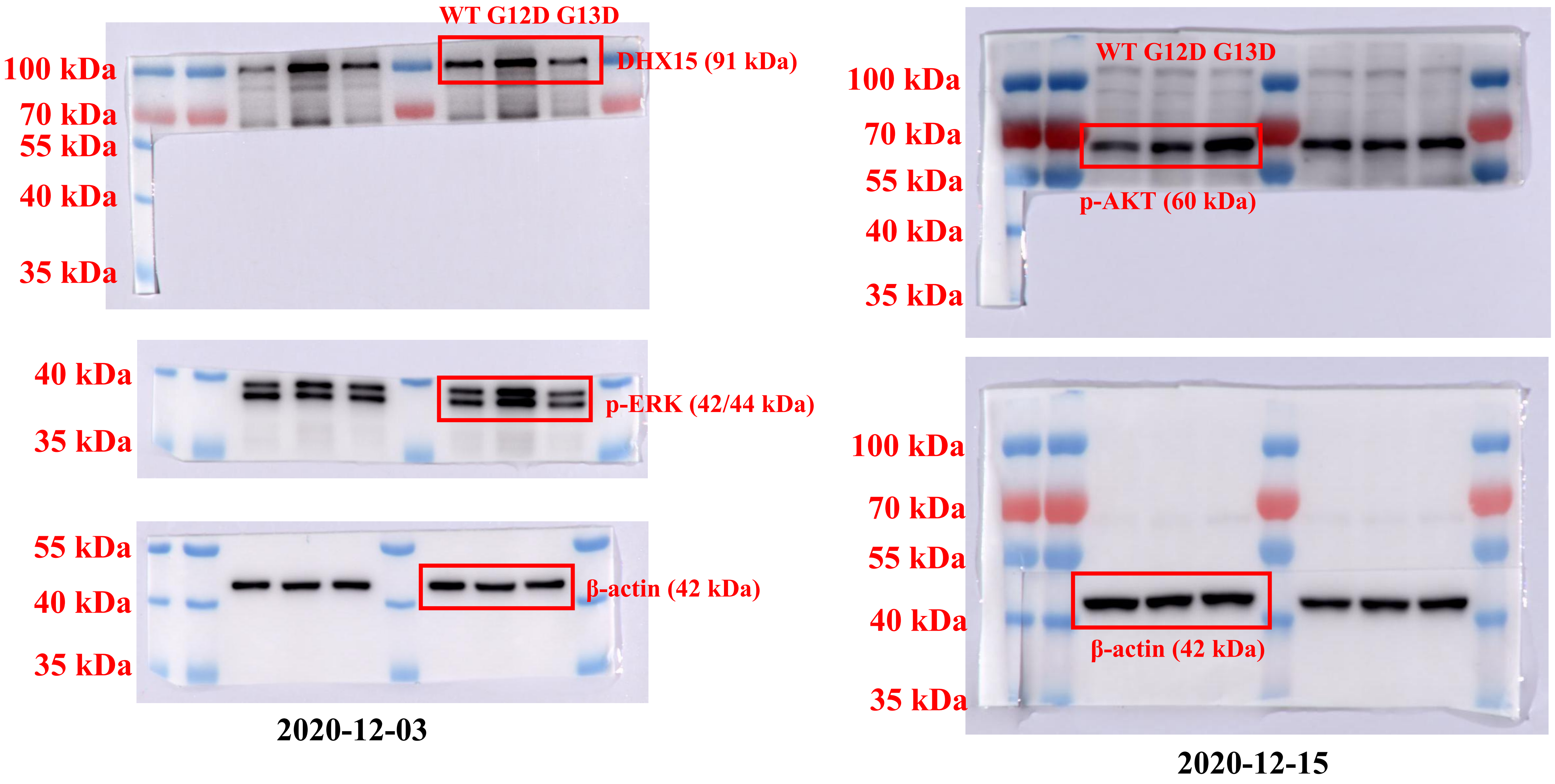

Figure 6b. Western blot analysis of p-ERK and DHX15 expression in *Villin-Cre/Kras*$^{G12D+/-}$ mice and WT littermates. Lanes representative of separate mice.

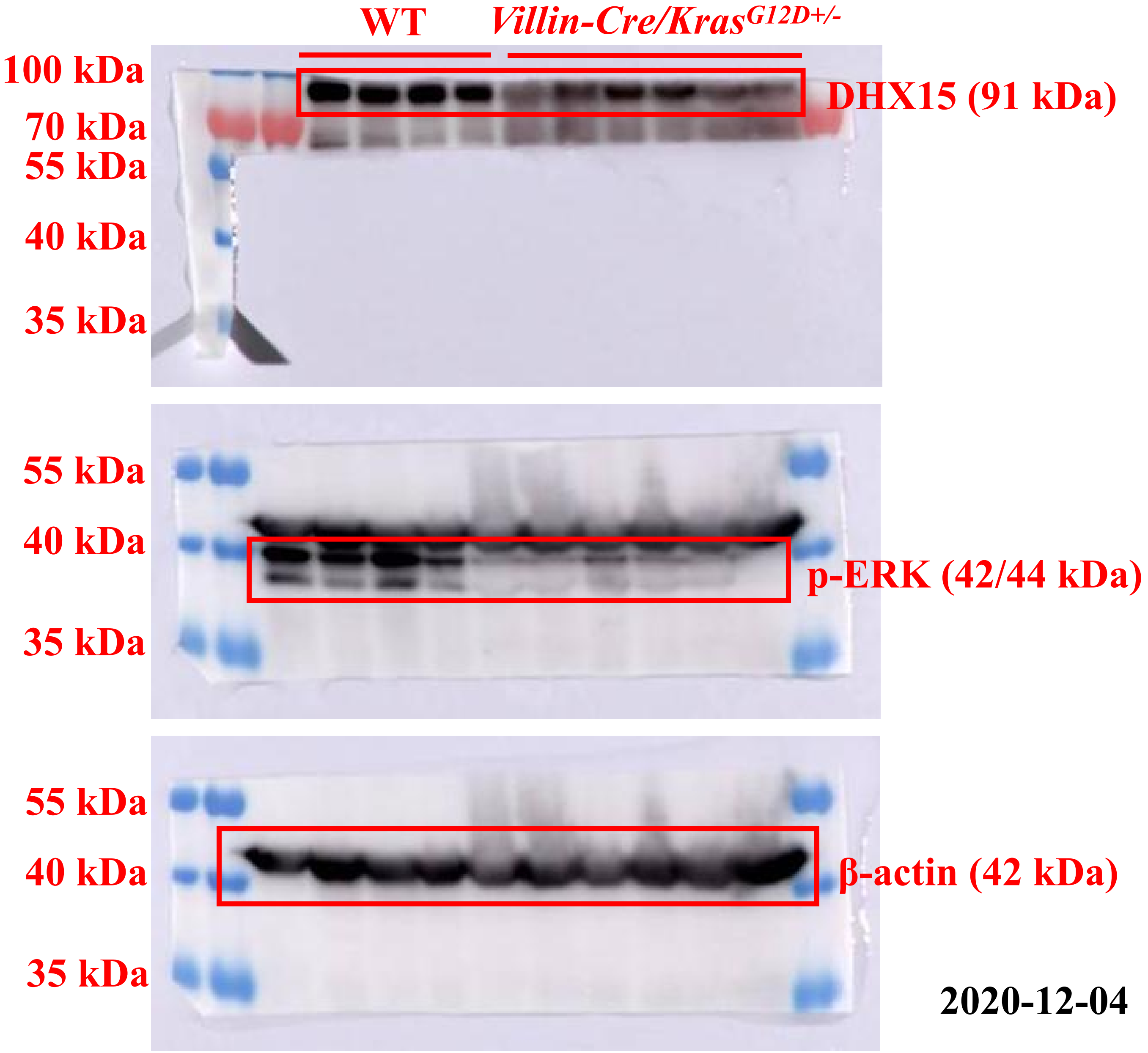

2020-12-04

Figure 6f. Western blotting analysis of DHX15 expression in *KRAS* p.G12D tumor cells after the specific inhibitor (SCH772987 for ERK and APTSTAT3-9R for STAT3) treatment.

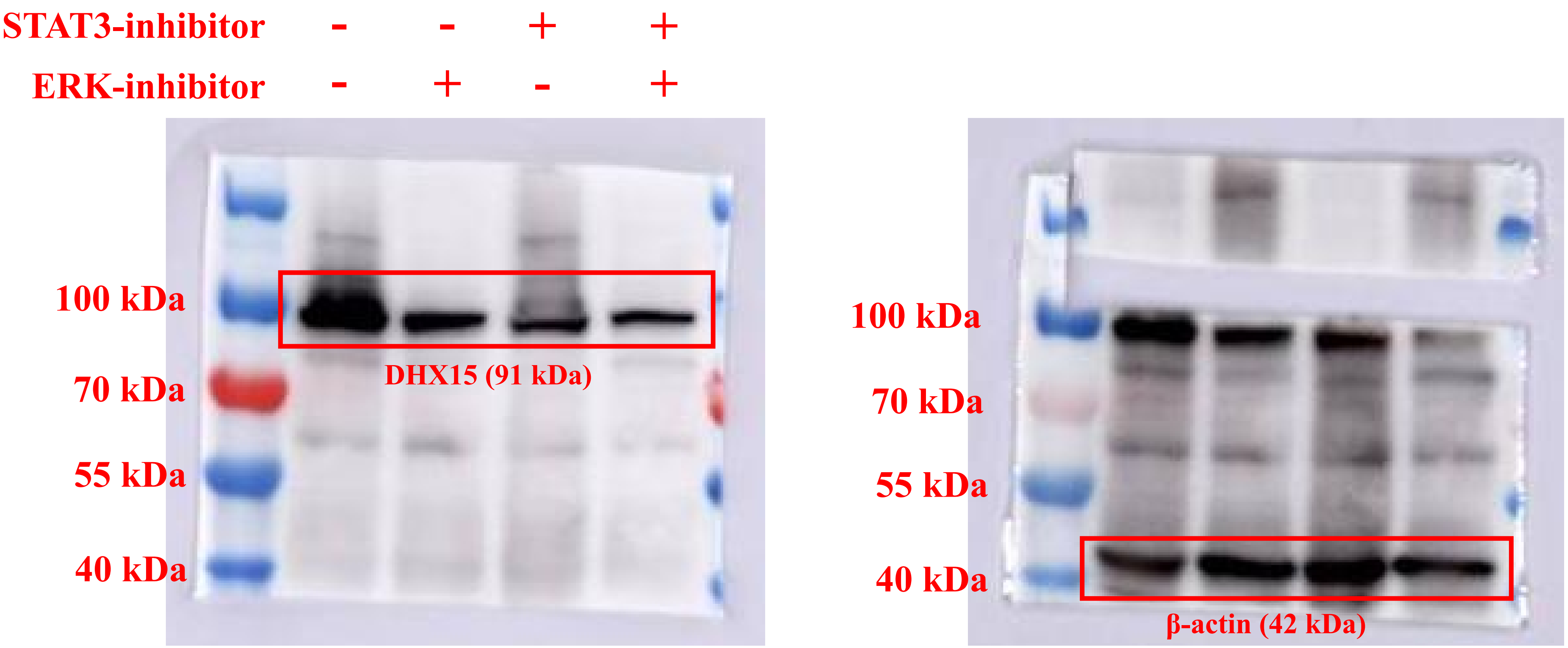

2021-4-16

Supplementary Figure 4a. HT-29 cells were transfected by *KRAS* G12D-sgRNA-Cas-EGFP and negative scramble control-EGFP lentiviral plasmids. Western blot analysis of KRAS expression in *KRAS* WT cells and *KRAS* p.G12D cells.

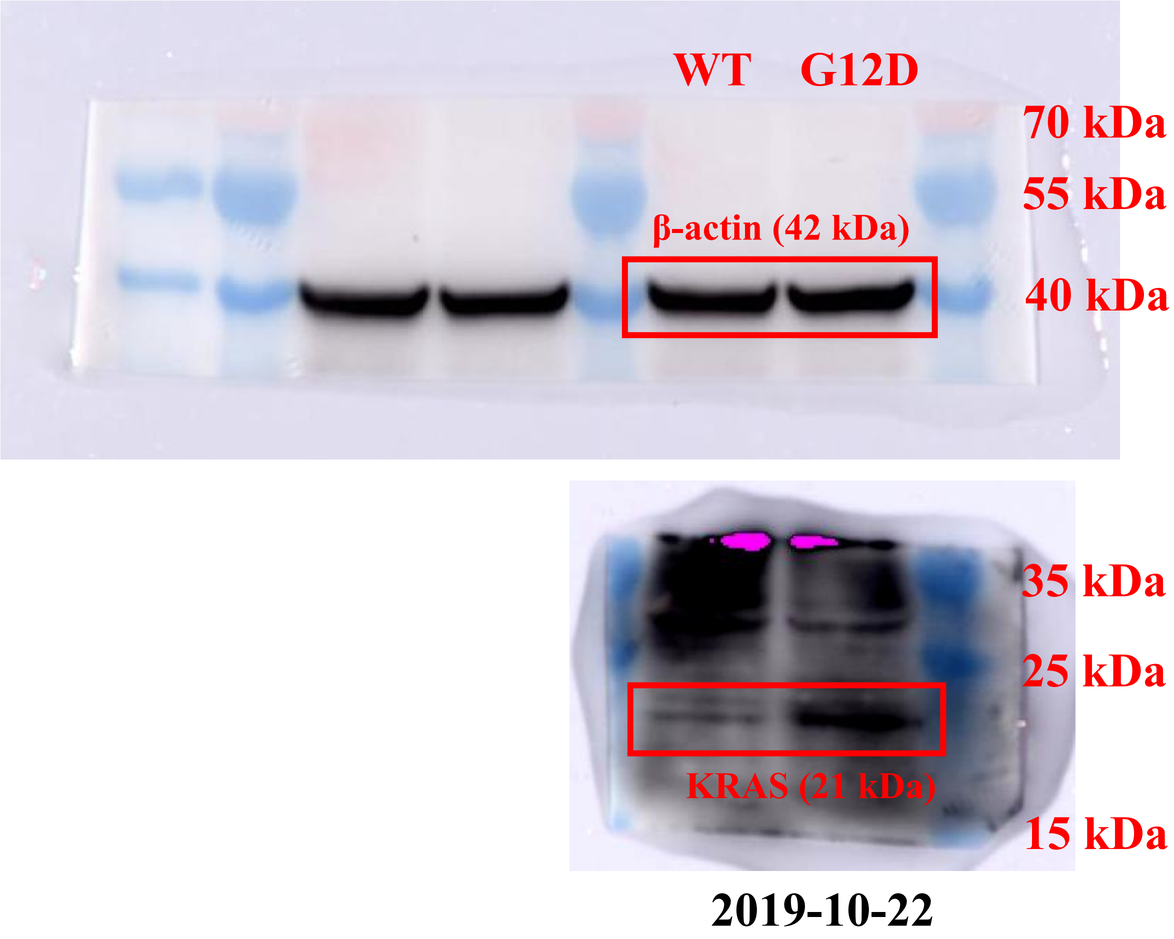

Supplementary Figure 4c. Western blot analysis of claudin-1 and ZO-1 expression in *KRAS* WT, *KRAS* p.G12D and *KRAS* p.G12D tumor cells.

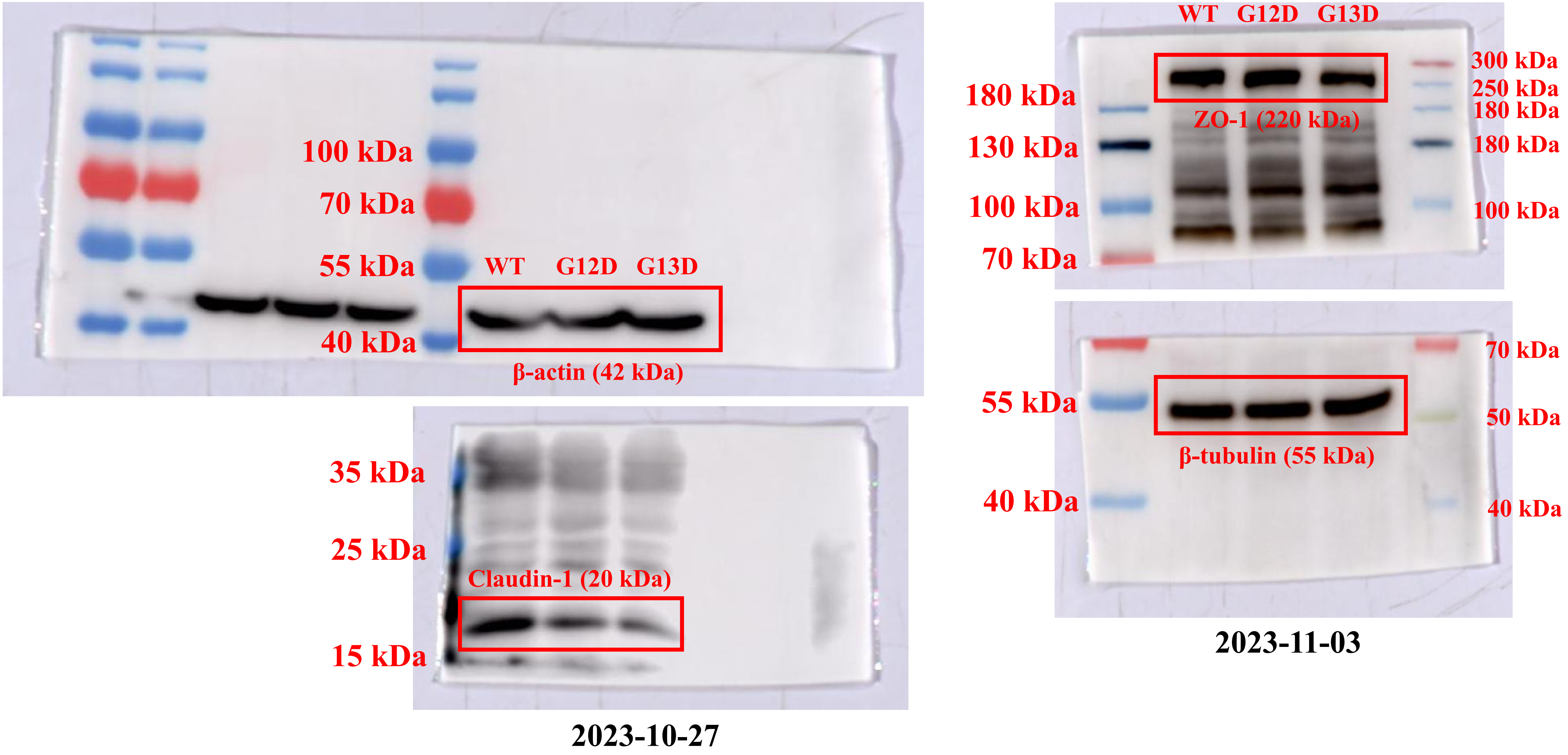

Supplementary Figure 4d. Pull-down assays were performed and validation of the FN1859-DHX15 interaction in *KRAS* WT, *KRAS* p.G12D and *KRAS* p.G12D CRC patient samples by western blot.

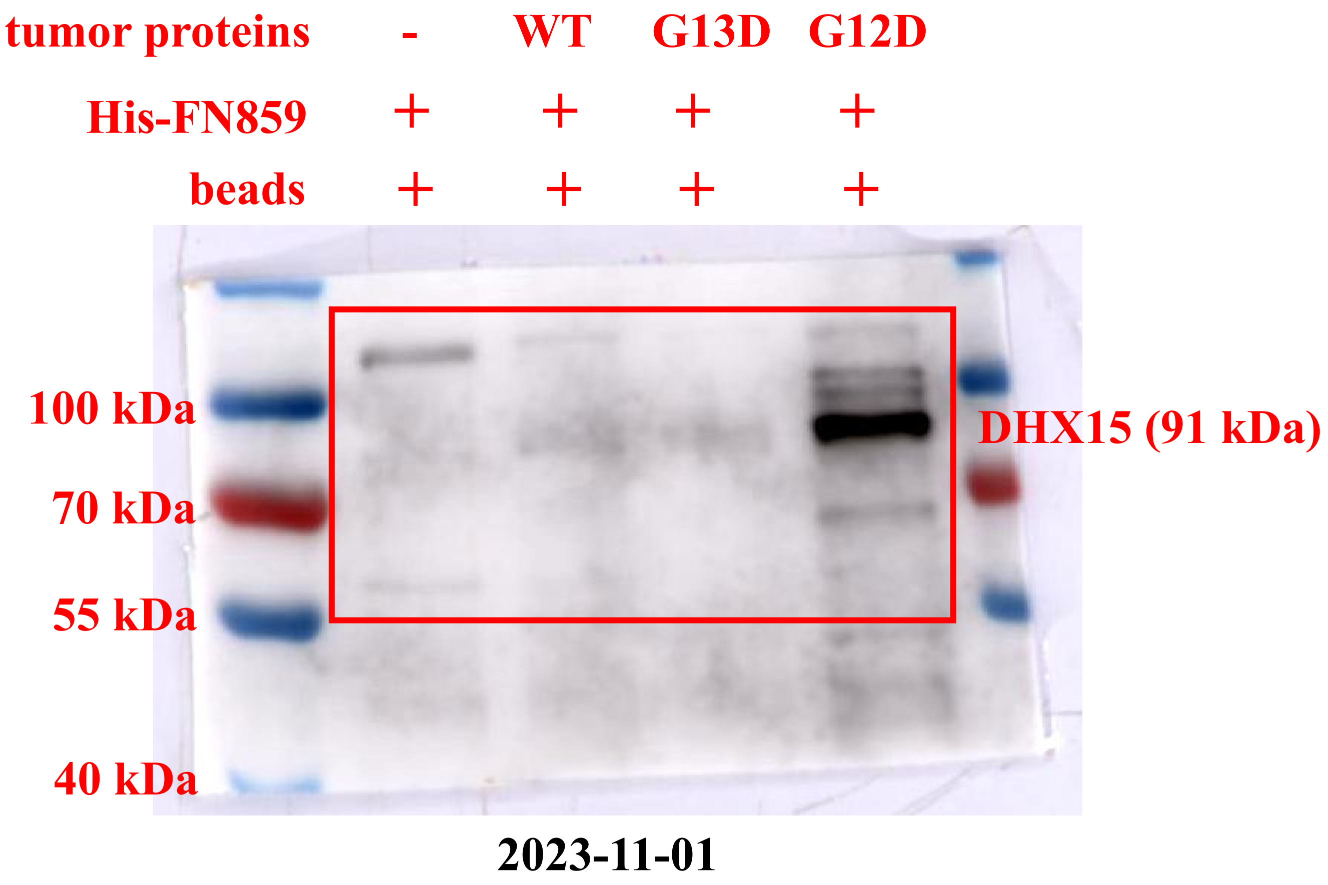

Supplementary Figure 4f. Pull-down assays were performed and validation of the *P. distasonis*-DHX15 interaction by silver staining.

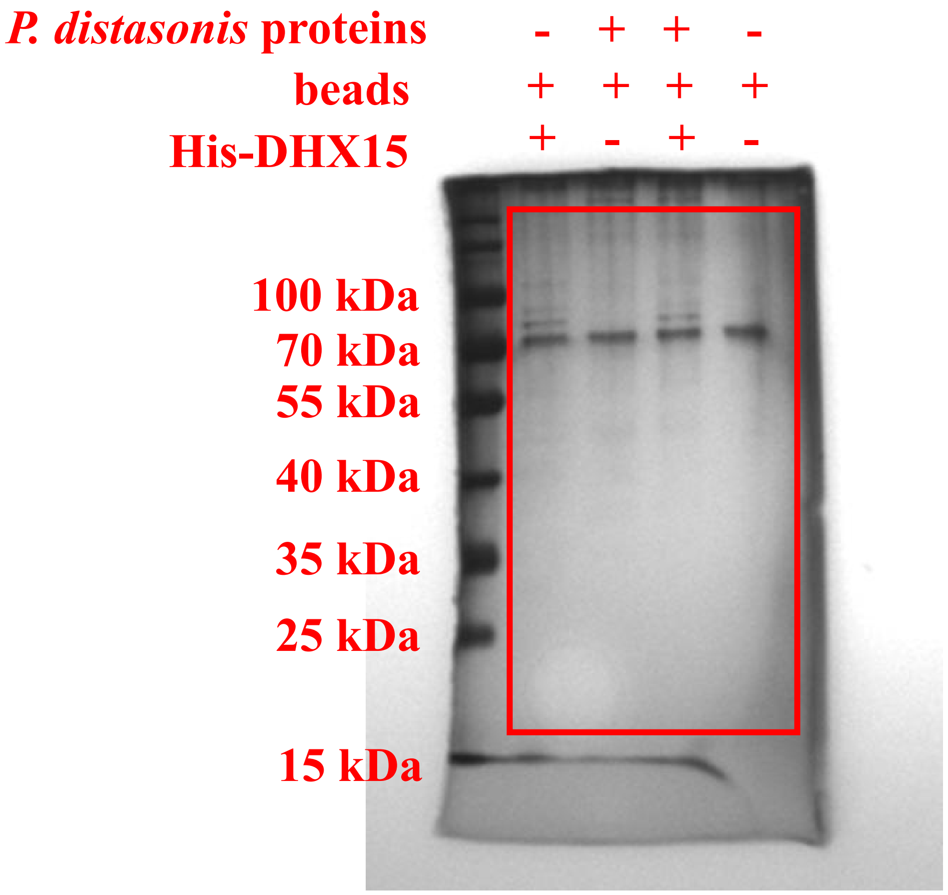

2023-11-01

Supplementary Figure 5a. Western blotting analysis of DHX15 expression in *KRAS* WT, *KRAS* p.G12D and *KRAS* p.G13D tumor cells after PBS or *F. nucleatum* treatment.

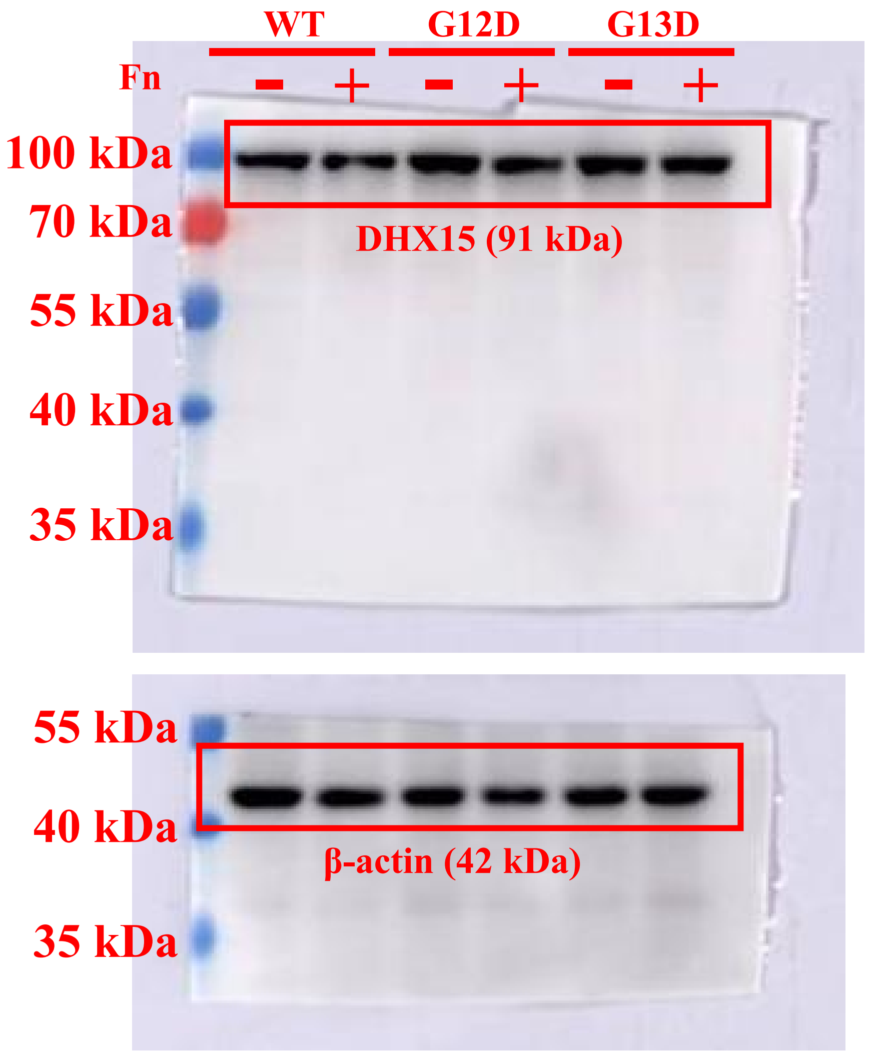

2022-07-07

Supplementary Figure 5b. Western blotting analysis of p-ERK expression in *KRAS* WT, *KRAS* p.G12D and *KRAS* p.G13D tumor cells after PBS or *F. nucleatum* treatment.

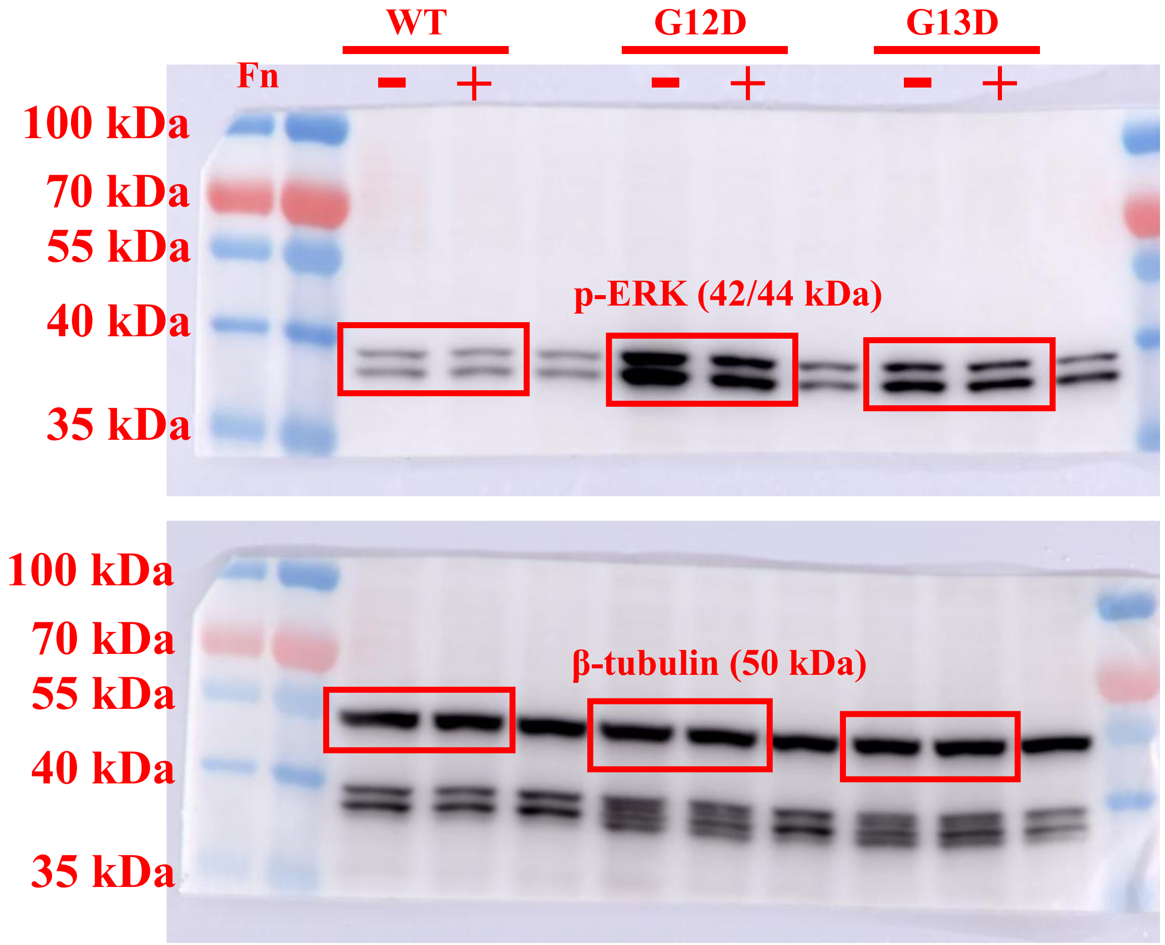

2020-09-13
