# Supplementary material for: Fusobacterium nucleatum promotes tumor progression in KRAS p.G12D-mutant colorectal cancer by binding to DHX15
Source: Nat Commun. 2024 Feb 24;15:1688. doi: 10.1038/s41467-024-45572-w (PMC10894276; doi:10.1038/s41467-024-45572-w)
Supplement: Supplementary file 2 — Peer Review File [file 41467_2024_45572_MOESM2_ESM.pdf]

nature portfolio

Peer Review File

Fusobacterium nucleatum Promotes Tumor Progression in KRAS p.G12D Mutant Colorectal Cancer by Binding to DHX15

Open Access This file is licensed under a Creative Commons Attribution 4.0 International License, which permits use, sharing, adaptation, distribution and reproduction in any medium or format, as long as you give appropriate credit to the original author(s) and the source, provide a link to the Creative Commons license, and indicate if changes were made. In the cases where the authors are anonymous, such as is the case for the reports of anonymous peer reviewers, author attribution should be to 'Anonymous Referee' followed by a clear attribution to the source work. The images or other third party material in this file are included in the article's Creative Commons license, unless indicated otherwise in a credit line to the material. If material is not included in the article's Creative Commons license and your intended use is not permitted by statutory regulation or exceeds the permitted use, you will need to obtain permission directly from the copyright holder. To view a copy of this license, visit http://creativecommons.org/licenses/by/4.0/.

REVIEWER COMMENTS

Reviewer #1 (Remarks to the Author): with expertise in CRC, Parabacteroides

Zhu et al are interested in the interaction between F.nucleatum and KRAS mutations in CRC and the modulation by Pdistasonis.

f.nucleatum more abundant when Kras mutations present. Fn gavage enhanced tumorigeneis in kras mice. Pdist depleted in Fn treated Kras mice. Also Pd reduced tumors compared to control which agrees with previous findings. Kras cells take up more Fn that WT cells. Target proteins were identified by incubating biotinylated KRAS cell proteins with Fn lysate. His tagged candidate protein FN1859 was incubated with cell lysates and protein dhx15 identified as the target. Dhx15 was elevated in kras human tissues. DHX15 k/o attenuated KRAS+Fn tumorigenesis.

Comments:

• line 115 use mutation not polymorphism

• review statistics throughout. Inappropriate use of Ttest in figure 1 – should be ANOVA. Also in other places where more than 2 groups are compared

• review and correct English grammar throughout

• fig 3a,b please include F.nucleatum abundance

• details of quality control for Pdist and Fn culture are required. How was their identity verified after culture to ensure absence of contamination?

• Please describe if/how Pdistasonis impacts the interaction between Fn and DHX15.

Overall this is a very interesting and robust studys. It has already been demonstrated that Pdist blocks CRC formation and the Fn promotes it, however the novel aspect here is that Pd is negatively correlated to Fn when Kras mutations present and that pd attenuates Fn driven CRC. Identifying the active component of Fn and its target in Kras cells is a major advance in our understanding of how Fn promotes CRC. Good mix of complementary human, organoid and mouse experiments. Well written and logical flow of experiments. Data on how Pdist might disrupt the FN1859 x DHX15 interaction would strengthen the manuscript further.

Reviewer #2 (Remarks to the Author): with expertise in CRC, Fusobacterium

In general, this is a very interesting provocative study on the potential tumorigenic mechanisms of Fusobacterium nucleatum (Fn). Findings have substantial implications if replicated and validated by independent studies. There are points for improvements.

Mutations should be described as KRAS p.G12D, KRAS p.G13D, KRAS p.G12V, etc. See Human Genome Variation Society (HGVS) mutation nomenclature guidelines. All clinical tumor molecular testing labs have been following these guidelines without exception.

The authors' findings possibly support previous evidence that Fn is most abundant in cecal cancers (Mima K et al. Clin Transl Gastroenterol 2016). Cecal cancers show the highest KRAS mutation frequency among CRCs in all colorectal sites (Yamauchi M et al. Gut 2012; Rosty C et al. Mod Pathol 2013; Ugai T et al. Am J Gastroenterol 2023). The authors should discuss these prior data supporting their study. The authors currently lack discussion on the link between Fn, cecal cancers, and KRAS mutations.

There are some differences from the two largest studies to date; Mima K et al. Gut 2016; Borozan I et al. Cancer Epidemiol Biomarkers Prev 2022. Both studies showed no relationship between Fn and KRAS mutations using >1000 and ~2000 CRC cases, respectively. Huangfu et al. (J Cancer 2021) conducted a meta-analysis and showed data based on 6 studies including Mima et al. study (not Borozan et al study), with OR 1.27 (1.00, 1.61), p =0.05. Given multiple testing and lack of multivariate analysis, the meta-analysis did not provide conclusive evidence. The authors should discuss potential differences due to sampled populations, tissue status (fresh frozen vs. FFPE), etc.

The initial mouse experiments compared only p.G12D vs. WT controls. The authors may need to test another mutation recombination – such as either one of Villin-Cre KRAS p.G13D, Villin-Cre KRAS p.G12V, Apc min/+, etc. model. We know KRAS p.G12D is a driver, so as Fusobacterium nucleatum. Thus, simply the authors showed driver effects of both. But they did not test how the effect (of KRAS p.G12D x Fn) differs from effect of another driver x Fn. (if this experiment is impossible to do in a short term, I would be fine without it).

Of note, BRAF p.V600E mutation and microsatellite instability (MSI) have been associated with Fusobacterium nucleatum tissue abundance in many prior studies. I don't know why the authors did not examine BRAF or MSI. As mentioned, BRAF mutation can activate MAPK signaling. MSI is an important immunogenic driver that is associated with Fn due to Fn's immunosuppressive effect.

Figure 3 D and E. The authors provided 2 p values, 1 each for D and E; but those p values did not test statistical significance of the difference between KRAS mut vs. WT. The authors should test statistical interaction using a logistic regression model having P. distasonis as an outcome with KRAS, F. nucleatum (Fn), and their interaction term (KRAS x Fn) as exposures. If needed, an expert statistician should be consulted.

Figure 3F. Arguments of the authors are not well supported. Statistical significance is heavily affected by statistical power. I don't see much difference in KRAS mut vs. WT.

Whether Fn can invade human colonic cells remains controversial and no conclusion has been made to my knowledge. The authors' experiment is interesting but we should keep in mind that it is cell line experiment. This is not a proof of cell invasion by Fn.

Human genes such as SLCO2A1 (not GPT or gpt), MYC (not c-myc), FOS (not c-fos), etc. need to be described by HUGO approved symbols – see www.genenames.org

Discussion states "… Recent studies revealed that high F. nucleatum abundance in the 332 tumor tissues was significantly associated with BRAF mutations and that the 333 prevalence of F. nucleatum was dramatically elevated in CpG island methylator 334 phenotype (CIMP)-positive CRC patients, supporting the notion that gut microbiota 335 has links to the intratumor genetics and epigenetics of CRC[27, 28]."

However, Ref 27 showed Fusobecteria species (not Fn) to be associated with CIMP. The authors should quote better references here such as those mentioned above, Eg, Mima et al. Gut 2016; Borozan et al. CEBP 2022.

Reviewer #3 (Remarks to the Author): with expertise in gut microbiota, intestinal neoplastic transformation

The work of Zhu et al tackles an interesting topic, ie the role of microbial entities, including Fusobacterium nucleatum, in the progression of KRAS mutants colorectal cancer patients. This is particularly relevant, since genetic mutations in CRC patients leads to complex relationships within the tumor microenvironment that, in the end, results in poor response to ICI. The overall goal of the authors is to identify or characterize the host and gut microbial components with which F. nucleatum interact in the process of CRC development. Authors analysed a cohort of 24 CRC patients and 239 CRC tissues by exon sequencing and qPCR, identifying a correlation between F. nucleatum abundance and KRAS specific mutations (G12D). By implementing a novel murine model (Villin-Cre/KrasG12D+/-) authors proceeded in the dissection of the functional interaction between F. nucleatum and the tumor microenvironment, taking advantage also of ex vivo human organoids.

Based on these analyses authors confirmed that F. nucleatum is enriched preferentially in KRAS G12D mutant CRC tumor tissues and contributes to colorectal tumorigenesis in Villin-Cre/KrasG12D+/- mice.

Authors then propose a role of P. distasonis in the competition of pro-tumorigenic functions with F. nucleatum in the G12D mouse model and human CRC tissues with the KRAS G12D mutation. Authors also suggest a potential mechanism by which F. nucleatum may contribute to tumorigenesis through the binding to DHX15, a protein of the RNA helicase family expressed on CRC tumor cells which is involved in the ERK/STAT3 signaling. Knock out DHX15 in Villin-Cre/KrasG12D+/- mice attenuated the CRC phenotype.

Data are of interest and can have impact on patients (more) personalized treatments. However, some technical and conceptual limitations reduce the strength of the manuscript. Here are some concerns:

Major:

1. Authors suggested a direct role of DHX15 in the pro-tumorigenic functions of F.nucleatum in G12D mutants. Although authors showed a direct interaction between F.nucleatum and DHX15, most of the events (before/after) remain unexplained. For instance:

_how authors reconcile their sentences: “FN1859 from F. nucleatum might interact with a cognate receptor on KRAS G12D cell surface line 251” And then “DHX15 is an outstanding

member of the DEAH-box RNA helicase family that is expressed in the nucleus line 257". Authors did not investigate how f. nucleatum enters in the cell before the interaction with DHX15 (Fap2?) and how entry and interaction with DHX15 occur temporally and influence CRC progression. Authors seem to imply that this interaction is required for f.nucleatum-mediated CRC progression, but it is not properly addressed . Authors also do not show any other surface protein on G12D mutants that may facilitate entry of F. nucleatum.
_authors do not show in mice how additional factors (immune system? Other microorganisms? fibroblasts? soluble factors?) contribute to the pro-tumorigenic activities of f.nucleatum in the presence or absence of DHX15.
_authors do not show directly any staining showing the interaction of DHX15 and f.nucleatum in primary samples form patients (or in organoids or cell lines)

2. The possible inhibitory role of P. diastonis is not addressed and seems highly overlooked in this system. Giving the data presented, I suggest to undertone the role of P. diastonis in this context (ie also from the title of the paper). It is not clear how the two microorganisms interact and , beside the data of the tumor burden reduction, it is not clear which is the mechanism. Are immune cells involved? Metabolites? Cross-competition for nutrients or for colonization niches?
3. Given the data on the role of f.nucleatum in the downregulation of the immune system (10.1126/scisignal.aba9157, 10.1016/j.mucimm.2023.03.006 just to name few involving neutrophils and T cells) it is important to further evaluate the interaction of f.nucleatum in Kras mutant patients and mice with the immune system (beside the two qPCR of cytokines which are really not enough to evaluate it)

Minor
_it is not clear how many replicates experiments have been performed in mice. It seems by the data presented they have been performed only once.
_it is not clear, and not openly explained in the M&M and in the text , why figure 2, figure 4 and figure 7 use different protocols of CRC induction (only one DSS administration versus more than one). This also largely influence the number of tumors (very low in figure 2, and higher in 4 and 7 both for PBS and F. nucleatum). In this context, p.diastonis in figure 4 induced a higher number of tumors than f. nucleatum in figure 2.

_relative abundance of f.nucleatum is very different in the different protocols (see fig 2 versus figure 7) and may be dependent on the type of protocol, but like this is quite misleading.

_f. nucleatum invasion of organoids is not really convincing, it seems that it is one scattered bacterium, and one may wonder how reproducible and consistent is the finding. Similar for diastonis.

REVIEWER COMMENTS

Reviewer #1 (Remarks to the Author): with expertise in CRC, *Parabacteroides*

Zhu *et al* are interested in the interaction between *F. nucleatum* and *KRAS* mutations in CRC and the modulation by *P. distasonis*.
F. nucleatum more abundant when *Kras* mutations present. *F. nucleatum* gavage enhanced tumorigeneis in *Kras* mice. *P. distasonis* depleted in *F. nucleatum* treated *Kras* mice. Also *P. distasonis* reduced tumors compared to control which agrees with previous findings. *Kras* cells take up more *F. nucleatum* that WT cells. Target proteins were identified by incubating biotinylated *KRAS* cell proteins with *F. nucleatum* lysate. His tagged candidate protein FN1859 was incubated with cell lysates and protein DHX15 identified as the target. DHX15 was elevated in *KRAS* human tissues. DHX15 K/O attenuated *KRAS*+*F. nucleatum* tumorigenesis.
Comments:

• Line 115 use mutation not polymorphism
Re: Thank you for pointing it out and we have corrected it in the revised manuscript (**Line 104**).

• Review statistics throughout. Inappropriate use of T test in Figure 1 – should be ANOVA. Also in other places where more than 2 groups are compared
Re: We really appreciate this constructive question raised by the reviewer. We agree with the reviewer's opinion, and we have corrected these throughout the revised manuscript including **Figure 1A**, **Figure 1D**, **Figure 4C**, **Figure 4E**, **Figure 4I**, **Figure 5B**, **Figure 5E**, **Figure 5H**, **Figure 6E** and **Figure 6I**.

• Review and correct English grammar throughout
Re: We thank this very helpful suggestion raised by the reviewer and correct the grammar throughout the manuscript as you suggested.

• Figure 3A, B please include *F. nucleatum* abundance
Re: Thank you for the kind suggestion. **Figure 3A** showed substantial compositional differences genera between WT litermates and *Villin-Cre/Kras*$^{G12D+/-}$ mice after *F. nucleatum* gavage. And **Figure 3B** showed substantial compositional differences genera between WT litermates and *Villin-Cre/Kras*$^{G12D+/-}$ mice after AOM/DSS treatment and *F. nucleatum* exposure. The findings suggested that the *Kras* p.G12D mutation affected the gut microbiota, which could have ramifications in *F. nucleatum*-related interactions. However, 16S rRNA sequencing data showed that comparable *Fusobacterium* genus abundance in different groups, so **Figure 3A** and **3B** did not include *Fusobacterium*.

• Details of quality control for *P. distasonis* and *F. nucleatum* culture are required. How was their identity verified after culture to ensure absence of contamination?

Re: Thank you for this helpful suggestion and we used Nanopore Sequencing for identification of *F. nucleatum* and *P. distosonis* recovered after co-culturing with cells and the results are shown in **Supplementary Table 6**.

Supplementary Table 6. Identification of cultured *F. nucleatum* and *P. distosonis* by Nanopore Sequencing.

SPECIES	SEQUENCE NUMBER	PROPORTION	NCBI ID	FAMILY	GENUS
Fusobacterium nucleatum	479265	97.58	851	*Bacteria*	*Fusobacterium*
Parabacteroides distasonis	2391	0.49	823	*Bacteria*	*Parabacteroides*
Escherichia coli	2224	0.45	562	*Bacteria*	*Escherichia*
unclassified	756	0.15	0	*unclassified*	*unclassified*
Fusobacterium sp. oral taxon 203	489	0.1	671211	*Bacteria*	*Fusobacterium*
Fusobacterium hwasookii	488	0.1	1583098	*Bacteria*	*Fusobacterium*
Fusobacterium pseudoperiodonticum	743	0.1	2663009	*Bacteria*	*Fusobacterium*
Parabacteroides sp. CT06	111	0.02	2025876	*Bacteria*	*Parabacteroides*
Luteimonas granuli	32	0.01	1176533	*Bacteria*	*Luteimonas*
Bacteroides fragilis	31	0.01	817	*Bacteria*	*Bacteroides*
**SPECIES**	**SEQUENCE NUMBER**	**PROPORTION**	**NCBI ID**	**FAMILY**	**GENUS**
Parabacteroides distasonis	99861	76.01	823	*Bacteria*	*Parabacteroides*
Parabacteroides sp. CT06	5210	3.97	2025876	*Bacteria*	*Parabacteroides*
unclassified	4319	3.29	0	*unclassified*	*unclassified*
Fusobacterium nucleatum	2266	1.72	851	*Bacteria*	*Fusobacterium*
Escherichia coli	1301	0.99	562	*Bacteria*	*Escherichia*
Bacteroides fragilis	1122	0.85	817	*Bacteria*	*Bacteroides*
Bacteroides uniformis	711	0.54	820	*Bacteria*	*Bacteroides*
Paraprevotella xylaniphila	495	0.38	454155	*Bacteria*	*Paraprevotella*
Bacteroides sp. HF- 5287	468	0.36	2650157	*Bacteria*	*Bacteroides*
Bacteroides xylanisolvens	276	0.21	371601	*Bacteria*	*Bacteroides*

• Please describe if/how *P. distasonis* impacts the interaction between *F. nucleatum* and DHX15.
Re: We thank this very helpful suggestion raised by the reviwer. To clarify whether *P. distasonis* involved in the interaction between *F. nucleatum* and DHX15, transmission electron microscope (TEM) were performed to assess the invasive ability of *P. distasonis* to tumor cells. We incubated *P. distasonis* with *KRAS* p.G12D mutant cells and data showed that there was no *P. distasonis* was observed in the G12D cells (**Supplementary Figure 4E**). Then we purified recombinant His-tagged DHX15 proteins from *Escherichia coli* and performed pull-down assays with *P. distasonis* lysates. There was no protein of *P. distasonis* was pulled by DHX15 (**Supplementary Figure 4F**). Therefore, we supposed that *P. distasonis* couldn't impact the interaction

between *F. nucleatum* and DHX15. However, bacterial recovery assays were performed to evaluate whether the invasive level of *F. nucleatum* was inhibited by *P. distasonis*. We incubated G12D cells with *F. nucleatum*, *F. nucleatum* + *P. distasonis* or *F. nucleatum* + supernatant of *P. distasonis* and the results showed that the invasive level of *F. nucleatum* could inhibited by *P. distasonis*, especially its supernatant implying that the metabolites of *P. distasonis* may involved in inhibiting *F. nucleatum* invasion **(Figure 5H).**

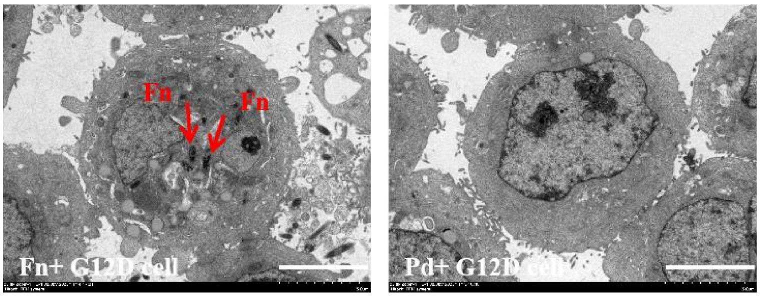

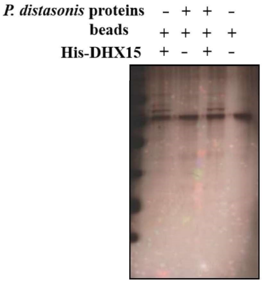

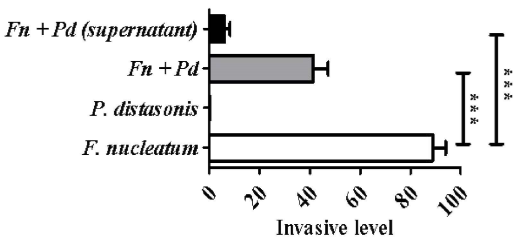

Overall this is a very interesting and robust study. It has already been demonstrated that *P. distasonis* blocks CRC formation and the *F. nucleatum* promotes it, however the novel aspect here is that *P. distasonis* is negatively correlated to *F. nucleatum* when *Kras* mutations present and that *P. distasonis* attenuates *F. nucleatum* driven CRC. Identifying the active component of *F. nucleatum* and its target in *Kras* cells is a major advance in our understanding of how *F. nucleatum* promotes CRC. Good mix of complementary human, organoid and mouse experiments. Well written and logical flow of experiments. Data on how *P. distasonis* might disrupt the FN1859 x DHX15 interaction would strengthen the manuscript further.

Reviewer #2 (Remarks to the Author): with expertise in CRC, *Fusobacterium*

In general, this is a very interesting provocative study on the potential tumorigenic mechanisms of *Fusobacterium nucleatum* (Fn). Findings have substantial implications if replicated and validated by independent studies. There are points for improvements.

Mutations should be described as *KRAS* p.G12D, *KRAS* p.G13D, *KRAS* p.G12V, etc. See Human Genome Variation Society (HGVS) mutation nomenclature guidelines. All clinical tumor molecular testing labs have been following these guidelines without exception.
Re: Thank you for the kind suggestion. We have followed the reviewer's suggestion and corrected the writings throughout the manuscript.

The authors' findings possibly support previous evidence that *F. nucleatum* is most

abundant in cecal cancers (Mima K *et al*. Clin Transl Gastroenterol 2016). Cecal cancers show the highest *KRAS* mutation frequency among CRCs in all colorectal sites (Yamauchi M *et al*. Gut 2012; Rosty C *et al*. Mod Pathol 2013; Ugai T *et al*. Am J Gastroenterol 2023). The authors should discuss these prior data supporting their study. The authors currently lack discussion on the link between *F. nucleatum*, cecal cancers, and *KRAS* mutations.

Re: We appreciate this very constructive suggestion raised by the reviewer. We have followed the reviewer's suggestion and discussed these in **Discussion (Line 386-392)**, and cited the references.

There are some differences from the two largest studies to date; Mima K *et al*. Gut 2016; Borozan I *et al*. Cancer Epidemiol Biomarkers Prev 2022. Both studies showed no relationship between *F. nucleatum* and *KRAS* mutations using >1000 and ~2000 CRC cases, respectively. Huangfu *et al*. (J Cancer 2021) conducted a meta-analysis and showed data based on 6 studies including Mima *et al*. study (not Borozan *et al* study), with OR 1.27 (1.00, 1.61), $p = 0.05$. Given multiple testing and lack of multivariate analysis, the meta-analysis did not provide conclusive evidence. The authors should discuss potential differences due to sampled populations, tissue status (fresh frozen vs. FFPE), etc.

Re: We thank for the very helpful suggestions raised by the reviewer. The reviewer is right that in different researches, the relationship between *F. nucleatumn* and *KRAS* mutations is not conclusive. Kosuk Mima *et al* have observed 1,069 CRC samples and found that the amount of *F. nucleatum* was not associated with *KRAS* mutation[1]. Ivan Borozan *et al* investigated 1,994 CRC cases and concluded that mutation status of *APC*, *PIK3CA*, *KRAS*, *BRAF*, *ERBB2*, and *SMAD4* were not associated with *F. nucleatum* prevalence[2]. However, the genomic DNA of *F nucleatum* was extracted from FFPE tissue sections of CRC in the two studies above. In another research, *KRAS* mutations were found to be more frequently observed in CRC samples infected with *F. nucleatum* and the genomic DNA of *F nucleatum* was extracted from 43 fresh-frozen CRC tissue samples and the matched adjacent normal tissues[3]. Consistent with this, we evaluated 24 fresh-frozen CRC tissue samples and the matched adjacent normal tissues and 239 CRC tumor tissues and verified that *F. nucleatum* abundance was associated with *KRAS* mutation. The potential differences in different researches maybe due to the tissue status (fresh frozen vs. FFPE) and we've revised the manuscript as the reviewer suggested and discussed this in the **Discussion (Line 394-408)**.

The initial mouse experiments compared only p.G12D vs. WT controls. The authors may need to test another mutation recombination – such as either one of *Villin-Cre Kras* p.G13D, *Villin-Cre Kras* p.G12V, *Apc* min/+, etc. model. We know *KRAS* p.G12D is a driver, so as *Fusobacterium nucleatum*. Thus, simply the authors showed driver effects of both. But they did not test how the effect (of *KRAS* p.G12D x *F. nucleatum*) differs from effect of another driver x *F. nucleatum*. (if this experiment is impossible to do in a short term, I would be fine without it).

Re: We really appreciate the reviewer's good question and also thank you for the

understanding. Actually, we try to establish another mutation of *KRAS* as you mentioned, such as conditional knockout of *KRAS* p.G13D in *Villin-Cre* mice to demonstrate the effect of another driver of *F. nucleatum*. Unfortunately, we failed to breed the *Villin-Cre/Kras*$^{G13D+/-}$ mice, which we couldn't explain the role of another driver of *F. nucleatum*. However, we verified the results in *KRAS* p.G12D and p.G13D mutation patients, as shown in **Figure 1D**. The tumor tissues with the p.G12D mutation, but not those with the p.G13D mutation, accommodated significantly higher *F. nucleatum* abundance than WT tumors. Additionally, *F. nucleatum* invaded more in the *KRAS* p.G12D cell line (on an average of 6.8/cell) than in *KRAS* p.G13D (on an average of 2.94/cell) or *KRAS* WT cell line (on an average of 0.9/cell) were also confirmed by confocal fluorescence microscopy showed in **Figure 5A and B**.

Of note, *BRAF* p.V600E mutation and microsatellite instability (MSI) have been associated with *Fusobacterium nucleatum* tissue abundance in many prior studies. I don't know why the authors did not examine *BRAF* or MSI. As mentioned, *BRAF* mutation can activate MAPK signaling. MSI is an important immunogenic driver that is associated with *F. nucleatum* due to *F. nucleatum*'s immunosuppressive effect.
Re: Thanks for the reviewer's question. The reviewer is right, *BRAF* p.V600E mutation and microsatellite instability (MSI) have been associated with *F. nucleatum* abundance in many previous studies. In fact, the four tested genes (*APC*, *TP53*, *KRAS* and *SMAD4*) in the manuscript are the four most frequently mutated genes in CRC patients, and their alterations often exhibit prognostic relevance according to The Cancer Genome Atlas (TCGA) database. In Chinese colorectal cancer patients, *KRAS* exon 2 mutations were occurred in 36.6% and *BRAF* (exon 15) mutations occurred in 4.0% of CRC patients[4]. As the reviewer mentioned above, mutation of *KRAS* and *BRAF* genes all lead to activation of MAPK signaling pathway to regulate cell growth, differentiation and apoptosis and patients with *KRAS* and *BRAF* mutations all showed poor response to anti-EGFR treatment. And the relationship and clinical significance of *F. nucleatum* with MSI have been reported in a lot of studies. The detailed molecular mechanism that links *F. nucleatum* and MSI is subjected to another study and is undertaken by another member of our research group.

Figure 3 D and E. The authors provided 2 *p* values, 1 each for D and E; but those *p* values did not test statistical significance of the difference between *KRAS* mut vs. WT. The authors should test statistical interaction using a logistic regression model having *P. distasonis* as an outcome with *KRAS*, *F. nucleatum* (Fn), and their interaction term (*KRAS* x Fn) as exposures. If needed, an expert statistician should be consulted.
Re: Thanks for the reviewer's very helpful comments. We have followed the reviewer's suggestion and consulted the statistical expert to test statistical interaction using a logistic regression model having *P. distasonis* as an outcome with *KRAS*, *F. nucleatum*, and their interaction term (*KRAS* x *F. nucleatum*) as exposures. As shown in **Supplementary Table 7**, *F. nucleatum* could be regarded as a protective factor for *P. distasonis* (OR=0.548), and the abundance of *P. distasonis* is negatively correlated

with *F. nucleatum* ($p < 0.05$). However, no significant difference was observed between the abundance of *P. distasonis* and *KRAS* mutation.

Supplementary Table 7. The data of logistic regression model that *P. distasonis* as an outcome with *KRAS*, *F. nucleatum*, and their interaction term (*KRAS* x *F. nucleatum*) as exposures

			Confidence limit (CI)	
	p value	OR	Lower limits	Upper limits
F. nucleatum	0.028	0.548	0.321	0.936
KRAS	0.542	0.849	0.502	1.437

Figure 3F. arguments of the authors are not well supported. Statistical significance is heavily affected by statistical power. I don't see much difference in *KRAS* mut vs. WT.
Re: Thank you for pointing it out and to avoid misleading, as you suggested, we went through the correlation analysis of *F. nucleatum* and *P. distasonis* status with clinical features including tumor differentiation, lymph node metasis and distance metasis in CRC patients by using Fisher's exact test (n = 239). Fisher's exact test is actually a statistical significance test when sample size are small, which is not applicable for our data analysis. We totally agree with your kind suggestion and we prefer to delete this data in the revised manuscript.

Whether *F. nucleatum* can invade human colonic cells remains controversial and no conclusion has been made to my knowledge. The authors' experiment is interesting but we should keep in mind that it is cell line experiment. This is not a proof of cell invasion by *F. nucleatum*.
Re: We thank the reviewer's kind suggestion, which is really a great question and is very helpful for us. As the reviewer suggested, we went through numerous literature and found that *F. nucleatum* was reported before to invade epithelial cells with high efficiency (up to 5% of input recovered) and may facilitate the colonization of epithelial cells by bacteria unable to invade directly[5]. Meanwhile, we performed TEM experiments on organoids derived from *KRAS* p.G12D mutation CRC patients and found *F. nucleatum* invaded inside the cytoplasm of the infected organoids **(Supplementary Figure 4B)**. The TEM data providing another proof for cell invasion of *F. nucleatum*.

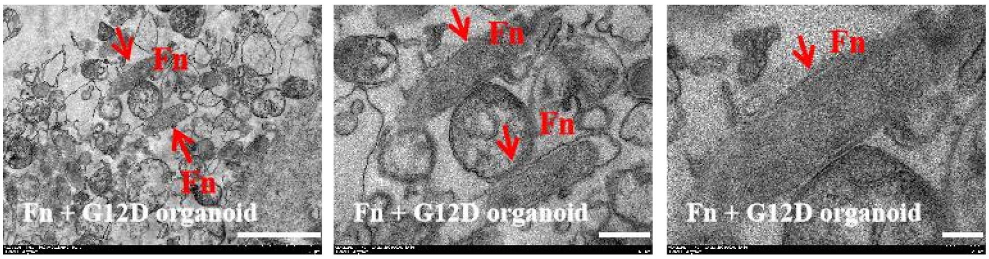

Human genes such as SLCO2A1 (not GPT or gpt), MYC (not c-myc), FOS (not c-fos), etc. need to be described by HUGO approved symbols – see www.genenames.org

Re: We thank the reviewer's advice, and have revised human genes such as c-myc as MYC, c-fos as FOS, Elk-1 as ELK1 as you suggested.

Discussion states "… Recent studies revealed that high *F. nucleatum* abundance in the 332 tumor tissues was significantly associated with *BRAF* mutations and that the 333 prevalence of *F. nucleatum* was dramatically elevated in CpG island methylator 334 phenotype (CIMP)-positive CRC patients, supporting the notion that gut microbiota 335 has links to the intratumor genetics and epigenetics of CRC[27, 28]." However, Ref 27 showed *Fusobecteria* species (not Fn) to be associated with CIMP. The authors should quote better references here such as those mentioned above, Eg, Mima et al. Gut 2016; Borozan et al. CEBP 2022.
Re: Thanks for the very helpful suggestions raised by the reviewer. We have followed the reviewer's suggestion and quoted the two meaningful studies (**Line 372-375**).

Reviewer #3 (Remarks to the Author): with expertise in gut microbiota, intestinal neoplastic transformation

The work of Zhu *et al* tackles an interesting topic, ie the role of microbial entities, including *Fusobacterium nucleatum*, in the progression of *KRAS* mutants colorectal cancer patients. This is particularly relevant, since genetic mutations in CRC patients leads to complex relationships within the tumor microenvironment that, in the end, results in poor response to ICI. The overall goal of the authors is to identify or characterize the host and gut microbial components with which *F. nucleatum* interact in the process of CRC development. Authors analysed a cohort of 24 CRC patients and 239 CRC tissues by exon sequencing and qPCR, identifying a correlation between *F. nucleatum* abundance and *KRAS* specific mutations (G12D). By implementing a novel murine model (*Villin-Cre/Kras*$^{G12D+/-}$) authors proceeded in the dissection of the functional interaction between *F. nucleatum* and the tumor microenvironment, taking advantage also of *ex vivo* human organoids.
Based on these analyses authors confirmed that *F. nucleatum* is enriched preferentially in *KRAS* G12D mutant CRC tumor tissues and contributes to colorectal tumorigenesis in *Villin-Cre/Kras*$^{G12D+/-}$ mice.
Authors then propose a role of *P. distasonis* in the competition of pro-tumorigenic functions with *F. nucleatum* in the G12D mouse model and human CRC tissues with the *KRAS* G12D mutation. Authors also suggest a potential mechanism by which *F. nucleatum* may contribute to tumorigenesis through the binding to DHX15, a protein of the RNA helicase family expressed on CRC tumor cells which is involved in the ERK/STAT3 signaling. Knock out *Dhx15* in *Villin-Cre/Kras*$^{G12D+/-}$ mice attenuated the CRC phenotype.
Data are of interest and can have impact on patients (more) personalized treatments. However, some technical and conceptual limitations reduce the strength of the manuscript. Here are some concerns:

Major:
1. Authors suggested a direct role of DHX15 in the pro-tumorigenic functions of

F.nucleatum in G12D mutants. Although authors showed a direct interaction between *F.nucleatum* and DHX15, most of the events (before/after) remain unexplained. For instance:

_how authors reconcile their sentences: "FN1859 from *F. nucleatum* might interact with a cognate receptor on *KRAS* G12D cell surface line 251" And then "DHX15 is an outstanding member of the DEAH-box RNA helicase family that is expressed in the nucleus line 257". Authors did not investigate how *F. nucleatum* enters in the cell before the interaction with DHX15 (Fap2?) and how entry and interaction with DHX15 occur temporally and influence CRC progression. Authors seem to imply that this interaction is required for *F. nucleatum*-mediated CRC progression, but it is not properly addressed. Authors also do not show any other surface protein on G12D mutants that may facilitate entry of *F. nucleatum*.

Re: We really appreciate this very helpful suggestions raised by the reviewer. To investigate the surface proteins on p.G12D mutant that may facilitate entry of *F. nucleatum*, we performed western blot to evaluate the expression of tight junction proteins. The reduction of tight junction proteins results in intestinal epithelial barrier dysfunction and bacterial translocation[6]. Results showed that *KRAS* p.G12D mutation cells expressed less ZO-1 and Claudin-1 proteins compared with *KRAS* p.G13D mutation and *KRAS* WT cells **(Supplementary Figure 4C)** which may facilitate entry of *F. nucleatum*.

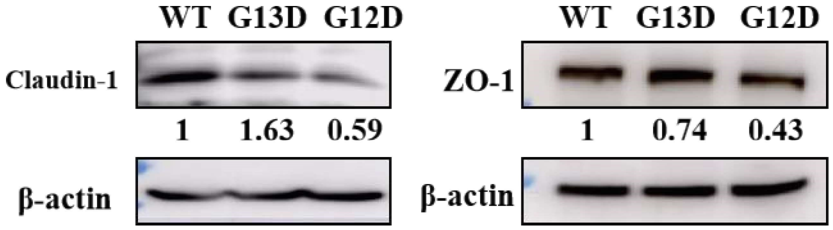

_authors do not show in mice how additional factors (immune system? Other microorganisms? fibroblasts? soluble factors?) contribute to the pro-tumorigenic activities of *F. nucleatum* in the presence or absence of DHX15.

Re: This is a very good suggestion. To investigate whether immune cells involved in the pro-tumorigenic activities of *F. nucleatum* in the presence or absence of DHX15, immunofluorescence (IF) and qPCR were performed to evaluate the infiltrated immune cells and inflammatory cytokine production in colonic tissues of *F. nucleatum*-treated *Villin-Cre/*$Kras^{G12D+/-}$ and *Villin-Cre/*$Kras^{G12D+/-}$*/*$Dhx15^{fl/fl}$ mice. We found that there were no significant differences in the number of infiltrated CD3 cells and CD11c dendritic cells in colonic tissues between the two groups **(Figure 7G and 7H)**. However, the expression of *Il-6*, *Il-17a* were elevated after *F. nucleatum* treatment in *Villin-Cre/*$Kras^{G12D+/-}$ mice when compared to *Villin-Cre/*$Kras^{G12D+/-}$*/*$Dhx15^{fl/fl}$ mice **(Figure 7I)** suggested the inflammatory cytokines may contribute to the pro-tumorigenic activities of *F. nucleatum*.

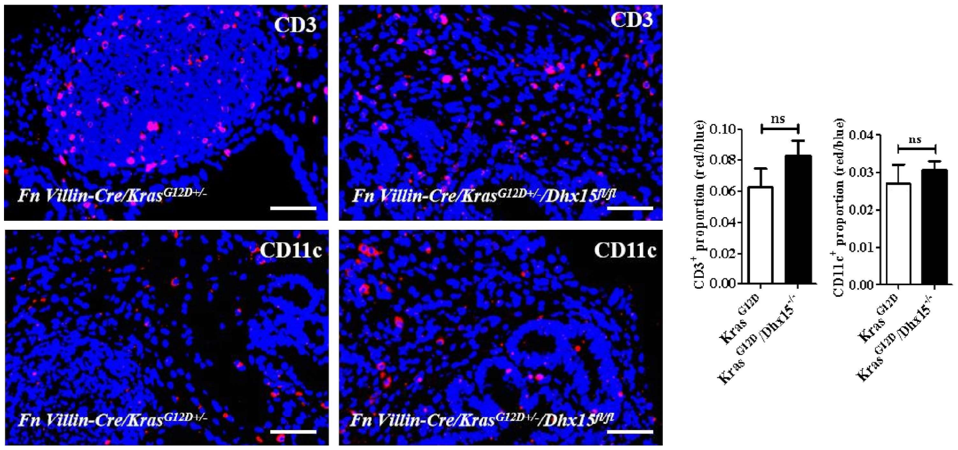

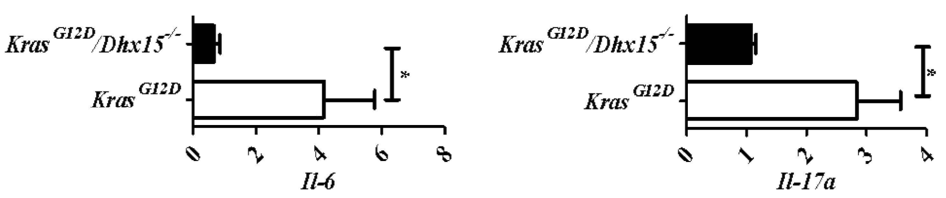

_authors do not show directly any staining showing the interaction of DHX15 and *F.nucleatum* in primary samples from patients (or in organoids or cell lines).

Re: Thanks for the reviewer's kind comments. According to the reviewer's suggestion, we have collected primary samples from CRC patients to support the interaction of DHX15 and *F.nucleatum*. We purified recombinant His-tagged FN1859 proteins from *Escherichia coli* and performed pull-down assays with primary samples from *KRAS* WT, *KRAS* p.G13D and *KRAS* G12D patients, shown in **Supplementary Figure 4D**. Results demonstrated that DHX15 exhibited specific coupling to FN1859 and that the protein-protein interaction was augmented in primary samples from *KRAS* p.G12D patients than that in the other two groups. In addition, the similar evidence was found in cell lines, as shown in **Figure 5G**, demonstrating the interaction of DHX15 and *F. nucleatum*. Furthermore, we have tried to stain DHX15 by IF and stain *F. nucleatum* by FISH on one slide. Unfortunately, for the limitation of technical strategies, we couldn't show the interaction of DHX15 and *F.nucleatum* on one slide.

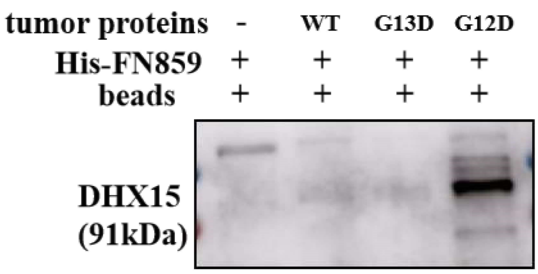

2. The possible inhibitory role of *P. diastonis* is not addressed and seems highly overlooked in this system. Giving the data presented, I suggest to undertone the role of *P. diastonis* in this context (ie also from the title of the paper). It is not clear how

the two microorganisms interact and, beside the data of the tumor burden reduction, it is not clear which is the mechanism. Are immune cells involved? Metabolites? Cross-competition for nutrients or for colonization niches?

Re: Thanks for the very helpful suggestions raised by the reviewer. The reviewer is right that we need to undertone the role of *P. diastonis* in the context. To investigate how the two microorganisms interact, we first assessed whether *P. distasonis* could invade tumor cells to impact the interaction between *F. nucleatum* and DHX15 by TEM. We incubated *P. distasonis* with *KRAS* p.G12D mutant cells and data showed that there was no *P. distasonis* was observed in the G12D cells (**Supplementary Figure 4E**). And we also purified recombinant His-tagged DHX15 proteins from *Escherichia coli* and performed pull-down assays with *P. distasonis* lysates. There was no protein of *P. distasonis* was pulled by DHX15 (**Supplementary Figure 4F**). Then to evaluate whether the invasive level of *F. nucleatum* could be inhibited by *P. distasonis*, we incubated G12D cells with *F. nucleatum*, *F. nucleatum* + *P. distasonis*, *F. nucleatum* + supernatant of *P. distasonis* and carried out the bacterial recovery assays. The results showed that the invasive level of *F. nucleatum* could be inhibited by *P. distasonis*, especially its supernatant implying that the metabolites of *P. distasonis* may involved in inhibiting *F. nucleatum* invasion (**Figure 5H**).

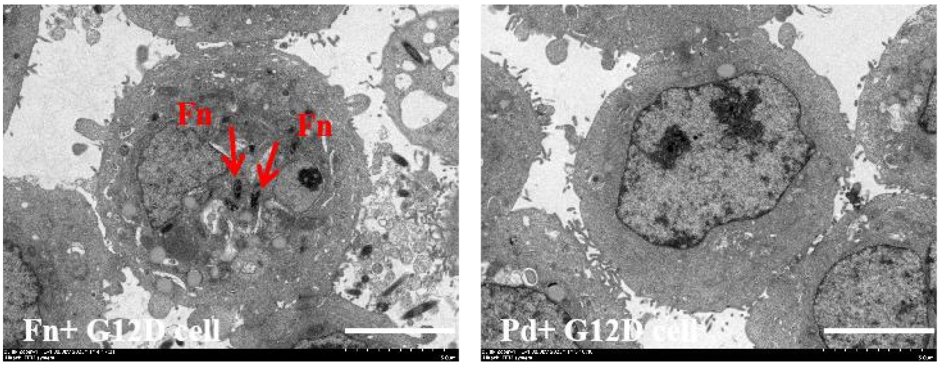

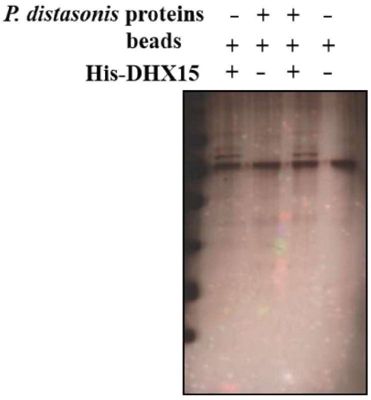

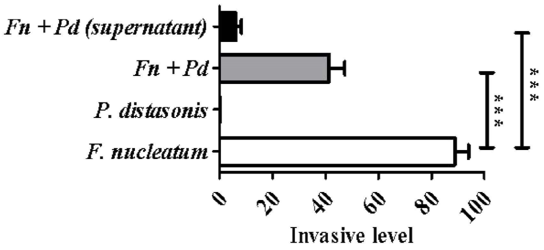

3. Given the data on the role of *F. nucleatum* in the downregulation of the immune system (10.1126/scisignal.aba9157, 10.1016/j.mucimm.2023.03.006 just to name few involving neutrophils and T cells) it is important to further evaluate the interaction of *F. nucleatum* in *KRAS* mutant patients and mice with the immune system (beside the two qPCR of cytokines which are really not enough to evaluate it)

Re: We thank the reviewer to bring up the question concerning the role of *F.*

nucleatum in regulation immune system. To evaluate the interaction of *F. nucleatum* with the immune system in *KRAS* p.G12D mutant mice and patients, IF and qPCR were performed to evaluate the infiltrated immune cells and inflammatory cytokine production in colonic tissues of PBS and *F. nucleatum*-treated *Villin-Cre/Kras*$^{G12D+/-}$ mice. We found that *F. nucleatum* led to less CD3$^+$ T cells infiltrating which is consisting with the study that *F. nucleatum* was inversely associated with tumor stromal CD3$^+$ lymphocytes **(Supplementary Figure 1D and 1E)**[7]. However, there was no significant differences in the number of infiltrated CD11c dendritic cells in colonic tissues between PBS and *F. nucleatum* treated *Villin-Cre/Kras*$^{G12D+/-}$ mice **(Supplementary Figure 1D and 1E)**. And the expression of *Il-17a*, *Il-6* were also elevated after *F. nucleatum* treatment in *Villin-Cre/Kras*$^{G12D+/-}$ mice **(Supplementary Figure 1F)**. Furthermore, we confirmed the similar results in tumor tissues from *KRAS* p.G12D mutant patients by IF **(Supplementary Figure 1G and H)**.

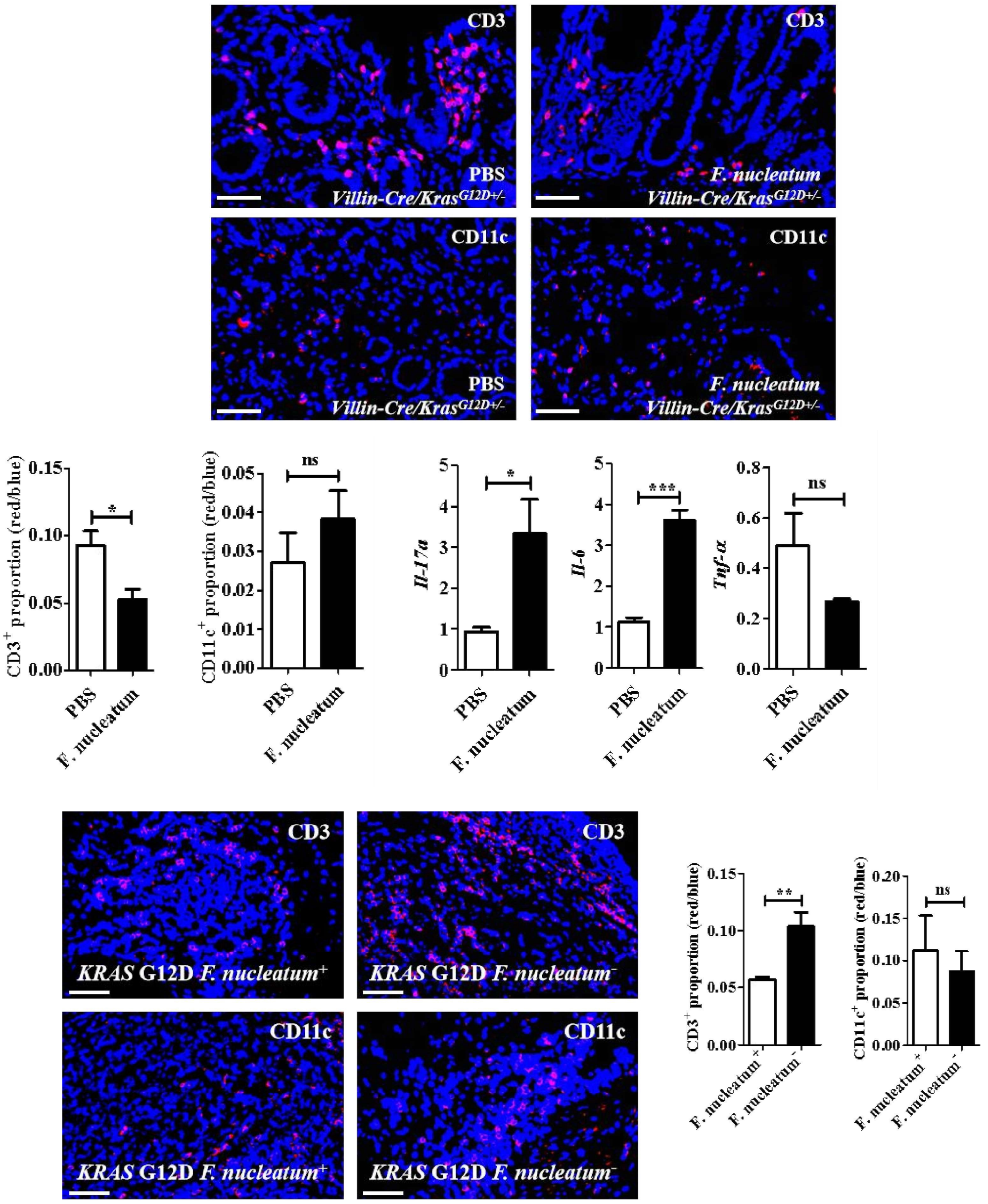

Minor

_It is not clear how many replicates experiments have been performed in mice. It seems by the data presented they have been performed only once.

Re: Thanks for the kind reminder from the reviewer. Data of mice experiments are representative of at least two independent experiments and we have revised the writings in the legend of **Figure 2, Figure 4, Figure 7**.

_It is not clear, and not openly explained in the M&M and in the text, why Figure 2, Figure 4 and Figure 7 use different protocols of CRC induction (only one DSS administration versus more than one). This also largely influence the number of tumors (very low in Figure 2, and higher in 4 and 7 both for PBS and *F. nucleatum*). In this context, *P. diastonis* in Figure 4 induced a higher number of tumors than *F. nucleatum* in Figure 2.

Re: The reviewer is right and in **Figure 2**, mice were administrated by DSS for one cycle to investigate whether *F. nucleatum* exacerbates colorectal tumorigenesis in *Villin-Cre/Kras*$^{G12D+/-}$ mice in early time. In **Figure 4** and **Figure 7**, to ensure successful tumor development and assess the treatment effect of *P. diastonis* and the role of DHX15 respectively, we administrated DSS for three cycles. We have followed the reviewer's suggestion and explained these in the revised manuscript, **Line 123-125, Line 205-207, Line 515-519.**

_Relative abundance of *F. nucleatum* is very different in the different protocols (see Figure 2 versus Figure 7) and may be dependent on the type of protocol, but like this is quite misleading.

Re: Thanks for the very helpful suggestions from the reviewer. The reviewer is right that the relative abundance of *F. nucleatum* is very different in **Figure 2** and **Figure 7.** In fact, these may be due to the different in control group chosen in the two figures above. In **Figure 2**, the control group are WT littermates and in **Figure 7**, the control group are *Villin-Cre/Kras*$^{G12D+/-}$*/Dhx15*$^{fl/fl}$ mice.

_*F. nucleatum* invasion of organoids is not really convincing, it seems that it is one scattered bacterium, and one may wonder how reproducible and consistent is the finding. Similar for *P. diastonis*.

Re: We appreciate the question raised by the reviewer and to verify the invasion of *F. nucleatum* in organoids, we performed TEM experiments on organoids derived from *KRAS* p.G12D mutation CRC patients and found that *F. nucleatum* invaded inside the cytoplasm of the infected *KRAS* p.G12D CRC organoids **(Supplementary Figure 4B).**

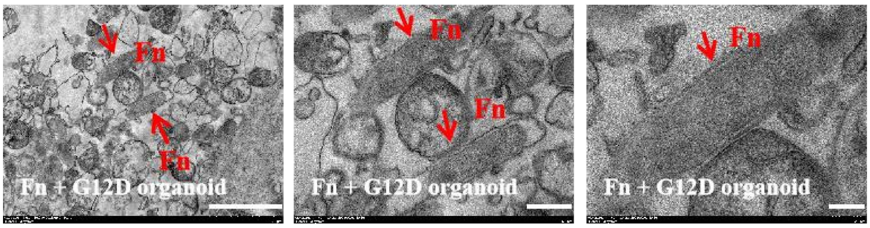

1. Mima K*, et al.* Fusobacterium nucleatum in colorectal carcinoma tissue and patient prognosis. *Gut* **65**, 1973-1980 (2016).

2. Borozan I*, et al.* Molecular and Pathology Features of Colorectal Tumors and Patient Outcomes Are Associated with Fusobacterium nucleatum and Its Subspecies animalis. *Cancer epidemiology, biomarkers & prevention : a publication of the American Association for Cancer Research, cosponsored by the American Society of Preventive Oncology* **31**, 210-220 (2022).

3. Proenca MA*, et al.* Relationship between Fusobacterium nucleatum, inflammatory mediators and microRNAs in colorectal carcinogenesis. *World journal of gastroenterology* **24**, 5351-5365 (2018).

4. Zhu X*, et al.* Correlation between clinicopathological features and KRAS, NRAS, and BRAF mutation status in Chinese colorectal cancer patients. *International journal of clinical and experimental pathology* **11**, 2839-2845 (2018).

5. Edwards AM, Grossman TJ, Rudney JD. Fusobacterium nucleatum transports noninvasive Streptococcus cristatus into human epithelial cells. *Infection and immunity* **74**, 654-662 (2006).

6. Drolia R, Tenguria S, Durkes AC, Turner JR, Bhunia AK. Listeria Adhesion Protein Induces Intestinal Epithelial Barrier Dysfunction for Bacterial Translocation. *Cell Host Microbe* **23**, 470-484 e477 (2018).

7. Borowsky J*, et al.* Association of Fusobacterium nucleatum with Specific T-cell Subsets in the Colorectal Carcinoma Microenvironment. *Clinical cancer research : an official journal of the American Association for Cancer Research* **27**, 2816-2826 (2021).

REVIEWERS' COMMENTS

Reviewer #1 (Remarks to the Author):

My concerns have been addressed satisfactorily.

Reviewer #2 (Remarks to the Author):

The authors successfully improved the paper, addressing the comments.

Reviewer #3 (Remarks to the Author):

Although some mechanisms remain not fully dissected, I do appreciate the effort made by authors to strengthen their data, not only by replying to my concerns but also to those of the other reviewers.

I have nothin else to add.

REVIEWER COMMENTS

Reviewer #1 (Remarks to the Author):

My concerns have been addressed satisfactorily.

Reviewer #2 (Remarks to the Author):

The authors successfully improved the paper, addressing the comments.

Reviewer #3 (Remarks to the Author):

Although some mechanisms remain not fully dissected, I do appreciate the effort made by authors to strengthen their data, not only by replying to my concerns but also to those of the other reviewers.
I have nothin else to add.

Re: We really appreciate all the reviewers for their efforts in improving our work.
